# 2-hydroxyglutarate mediates whitening of brown adipocytes coupled to nuclear softening upon mitochondrial dysfunction

**DOI:** 10.1038/s42255-025-01332-8

**Published:** 2025-08-01

**Authors:** Harshita Kaul, Lea Isermann, Katharina Senft, Milica Popovic, Theodoros Georgomanolis, Linda Baumann, Pujyanathan Sivanesan, Andromachi Pouikli, Hendrik Nolte, Bojana Lucic, Ximena Hildebrandt, Katrin Seidel, Thorsten Gnad, Felix Gaedke, Ulrike Göbel, Franziska Peters, Maksym Cherevatenko, Joo Hyun Park, Astrid Schauss, Nieves Peltzer, Jens Claus Brüning, Jan-Wilhelm Kornfeld, Alexander Pfeifer, Thomas Langer, Marina Lusic, Sara A. Wickström, Christian Frezza, Aleksandra Trifunovic

**Affiliations:** 1 Institute for Mitochondrial Diseases in Ageing, Faculty of Medicine, University of Cologne, and University Hospital Cologne, Cologne, Germany; 2 Cologne Excellence Cluster on Cellular Stress Responses in Ageing-Associated Diseases (CECAD), University of Cologne, Cologne, Germany; 3 Institute for Metabolomics in Ageing, Faculty of Medicine, University of Cologne, and University Hospital Cologne, Cologne, Germany; 4 Max Planck Institute for Biology of Ageing, Cologne, Germany; 5 Department of Infectious Diseases, Center for Integrative Infectious Disease Research (CIID), Integrative Virology, Heidelberg University, Heidelberg, Germany; 6 Department of Genome Editing, Institute of Biomedical Genetics (IBMG), University of Stuttgart, Stuttgart, Germany; 7 Centre for Molecular Medicine Cologne (CMMC), University of Cologne, Cologne, Germany; 8 Bonn University Hospital (AöR), Biomedical Center (BMZ), Bonn, Germany; 9 Federal Institute for Drugs and Medical Devices, Bonn, Germany; 10 Department of Cell and Tissue Dynamics, Max Planck Institute for Molecular Biomedicine, Münster, Germany; 11 Department of Neuronal Control of Metabolism, Max Planck Institute for Metabolism Research, and Center for Endocrinology, Diabetes and Preventive Medicine (CEDP), University Hospital Cologne, Cologne, Germany; 12 University of Southern Denmark, Odense, Denmark; 13 Stem Cells and Metabolism Research Program, Faculty of Medicine and Helsinki Institute of Life Science, Biomedicum Helsinki, University of Helsinki, Helsinki, Finland; 14 Institute of Genetics Faculty of Mathematics and Natural Sciences, University of Cologne, Cologne, Germany

**Keywords:** Energy metabolism, Stress signalling, Nuclear envelope, Metabolism

## Abstract

Mitochondria have a crucial role in regulating cellular homeostasis in response to intrinsic and extrinsic cues by changing cellular metabolism to meet these challenges. However, the molecular underpinnings of this regulation and the complete spectrum of these physiological outcomes remain largely unexplored. In this study, we elucidate the mechanisms driving the whitening phenotype in brown adipose tissue (BAT) deficient in the mitochondrial matrix protease CLPP. Here we show that CLPP-deficient BAT shows aberrant accumulation of lipid droplets, which occurs independently of defects in oxygen consumption and fatty acid oxidation. Our results indicate that mitochondrial dysfunction due to CLPP deficiency leads to the build-up of the oncometabolite d-2-hydroxyglutarate (d-2HG), which in turn promotes lipid droplet enlargement. We further demonstrate that d-2HG influences gene expression and decreases nuclear stiffness by modifying epigenetic signatures. We propose that lipid accumulation and altered nuclear stiffness regulated through 2HG are stress responses to mitochondrial dysfunction in BAT.

## Abstract (web summary)

CLPP-deficiency-driven mitochondrial dysfunction in brown adipose tissue leads to the accumulation of d-2-hydroxyglutarate, in turn promoting lipid-droplet enlargement by altering gene expression and epigenetically regulating nuclear stiffness.

## Main

Mitochondria have traditionally been regarded as distinct cellular entities primarily responsible for energy production^1^. However, increasing evidence shows that they are dynamic organelles that adapt to metabolic changes and cellular stress^2^. Consequently, mitochondria maintain cellular homeostasis through communication with the nucleus and other organelles^2^.

Given their fundamental role, it is not surprising that mitochondrial dysfunction, especially oxidative phosphorylation (OXPHOS) deficiency, is linked to ageing and many diseases^3^. Mitochondrial stress triggers nuclear responses that alter gene expression to counteract cellular damage^4^. In mammalian cells, the mitochondrial integrated stress response (mitoISR) is one of the earliest transcriptional programmes activated by OXPHOS disruption^5^. This leads to metabolic reprogramming that can support or hinder tissue homeostasis^6,7^.

The physiological consequences of mitochondrial stress remain incompletely understood, particularly in a tissue-specific context. White and brown adipose tissues (WAT and BAT) depend heavily on mitochondrial function and exhibit remarkable plasticity to meet physiological demands^8^. In WAT, mitochondria regulate energy storage and mobilization through triglyceride accumulation and lipolysis^9^. By contrast, BAT, rich in mitochondria, drives thermogenesis through uncoupling protein 1 (UCP1), which dissipates energy as heat, a process essential for neonatal and cold-induced thermoregulation^10^. Both tissues exhibit metabolic plasticity: WAT undergoes browning in response to cold, β-adrenergic stimulation, exercise or fasting, increasing mitochondrial content and uncoupling. Conversely, BAT whitening occurs under thermoneutrality, ageing or nutrient overload, leading to lipid accumulation and loss of thermogenic function^11^.

Chronic exposure to thermoneutrality (~30 °C) induces BAT whitening through PARKIN-mediated mitophagy and a ChREBP-driven shift toward fatty acid synthesis^12,13^. Factors such as obesity, high-fat diets and ageing similarly promote BAT whitening, characterized by lipid accumulation, reduced vascularization and impaired thermogenesis^14–16^. Genetic loss of lipolytic and oxidative enzymes (for example, ATGL and CPT2)^17,18^, or key transcriptional regulators (for example, PGC-1 and PRDM16)^19,20^, disrupts BAT differentiation and mitochondrial biogenesis, leading to whitening. Regardless of the underlying cause, a common hallmark is lipid-droplet expansion with reduced mitochondrial mass or function^21^. Consistently, loss of mitochondrial proteins essential for fusion (MFN2 and OPA1)^22,23^ or mitochondrial DNA (mtDNA) maintenance (TFAM)^24^ also triggers BAT whitening. These findings highlight the central role of OXPHOS integrity in BAT homeostasis, although the underlying mechanisms that connect mitochondrial function to the homeostasis in BAT remain incompletely understood.

We have recently shown that the loss of the mitochondrial matrix protease CLPP has contrasting effects in adipose tissue: it enhances oxidative phosphorylation (OXPHOS) in WAT but impairs BAT thermogenesis and promotes lipid accumulation, consistent with a whitening phenotype^25^. CLPP levels rise in BAT with cold exposure, unlike other mitochondrial proteases, but decline with ageing^26,27^. In a deep BAT proteomics screen involving a cohort of 163 genetically defined diversity outbred mice, CLPP correlated strongly with UCP1 (https://wren.hms.harvard.edu/opabat/)^28^. Furthermore, several of the identified UCP1 interactors are known CLPP substrates, supporting a strong link between CLPP and BAT function^25,28,29^.

Here, we show that CLPP deficiency in BAT drives lipid droplet (LD) expansion through respiratory-chain dysfunction, independent of changes in oxygen consumption. This phenotype is driven by accumulation of d-2HG, an oncometabolite that alters chromatin and nuclear architecture. We reveal that mitochondrial dysfunction modulates epigenetic programmes, reducing nuclear stiffness and promoting BAT whitening. Together, our results uncover a previously unrecognized connection between mitochondrial function, metabolic signalling and chromatin reorganization in BAT. These insights shed light on how CLPP deficiency contributes to BAT whitening and impaired thermogenic function, expanding our understanding of mitochondrial regulation in adipose tissue biology.

## Results

### CLPP loss in BAT causes mild dysfunction and whitening

**Fig. 1:**
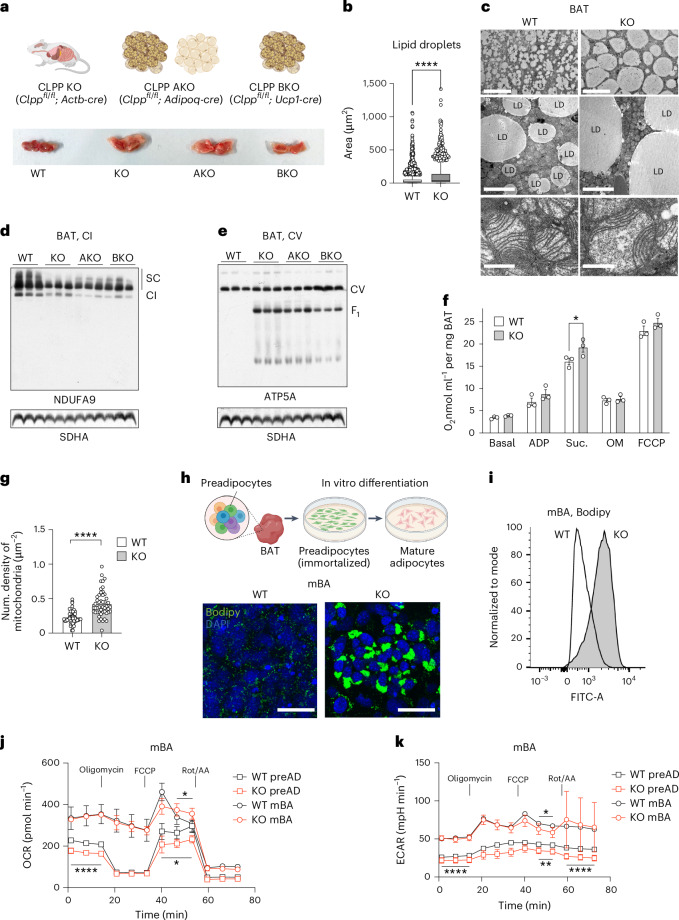
Loss of CLPP leads to cell-autonomous BAT whitening. **a**, Representative whole-BAT images from wild-type (WT), ubiquitously-CLPP-deficient mice (KO), adipose-specific CLPP-KO (AKO) mice or BAT-specific CLPP-KO (BKO) mice. **b**, LD size measurement from WT and CLPP-KO BAT. *n* = 2,289 LDs for WT and *n* = 724 LDs for KO. **c**, Representative transmission electron microscopy images of BAT tissue from WT and CLPP-KO mice. Scale bars, 50 μm, 5 μm and 0.5 μm (from top to bottom). **d**,**e**, Steady-state levels of OXPHOS supercomplexes (SC) in the BAT mitochondria of WT and CLPP-KO, CLPP-AKO and CLPP-BKO mice, analysed by blue native–PAGE followed by western blot for CI (NDUFA9) (**d**) and CV (ATP5A) (**e**) (*n* = 3). **f**, OCRs of whole BAT lysates from WT and CLPP-KO mice (*n* = 3). **g**, Numerical density of mitochondria in WT and CLPP-KO BAT (*n* = 48 fields per genotype obtained from three different mice from each genotype). **h**, Representative images of cultured and in-vitro-differentiated WT and CLPP-KO mBA cells, stained with DAPI (nuclei) and Bodipy (LDs). Scale bars, 100 μm. Images represent results obtained from four independent experiments. **i**, Flow cytometry analysis of lipid accumulation (Bodipy) in WT and CLPP-KO mBA cells. Data represent one out of four performed experiments. **j**,**k**, OCRs (**j**) and ECARs (**k**) of WT and CLPP-KO preADs and mBAs (*n* = 8). **b**, Data are presented using Tukey’s box plot with the middle line marking the median, and whiskers show variability within 1.5 × IQR. Anything beyond is an outlier presented as the individual value. **f**,**g**,**j**,**k**, Data are presented as mean ± s.d. *P < 0.05, **P < 0.01, ***P < 0.001, ****P < 0.0001, as determined by unpaired two-tailed Student’s *t*-test in **b**, **g**, **j** and **k**, and one-way analysis of variance (ANOVA) with multiple comparisons in **f**. In **j** and **k**, the statistical significance between WT and knockout cells of the same cell types were plotted either above (mBA cells) or below the graph lines (preAD). **a**,**h**, Schematics were created using Biorender.com. Source data

**Extended Data Fig. 1:**
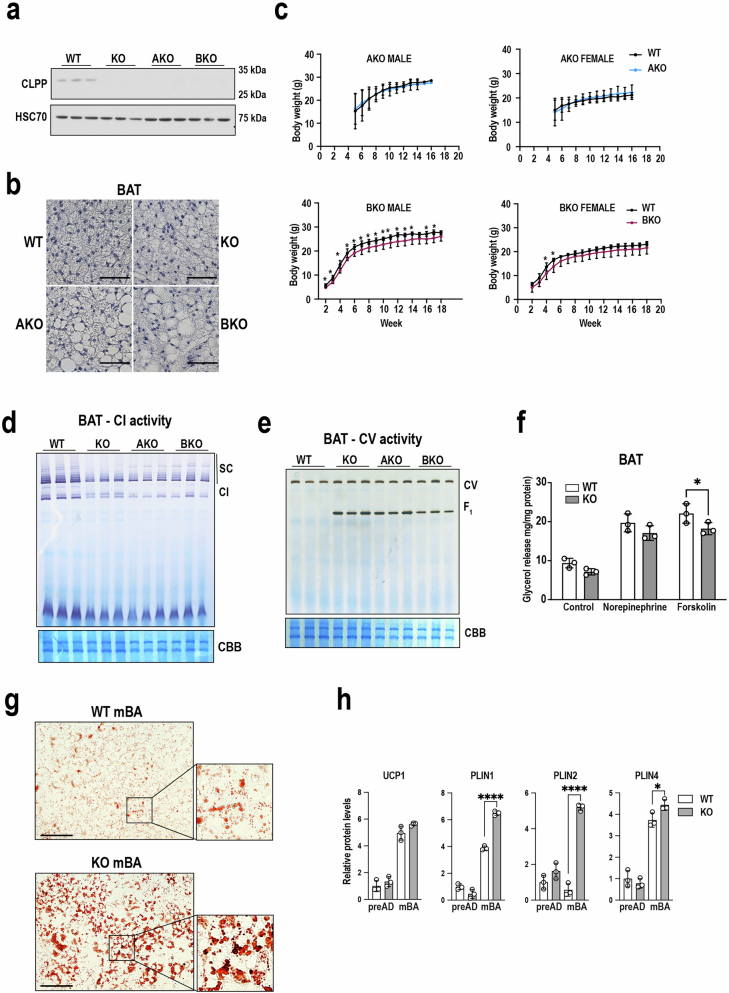
Effects of CLPP loss show similar changes in AKO and BKO. **(a)** Immunoblot of CLPP from wild type (WT), ubiquitously-deficient CLPP mice (KO), adipose-specific CLPP KO (AKO) or BAT specific CLPP KO (BKO) mice (n = 3, blots representative of four independent experiments); **(b)** H&E staining of BAT from WT, KO, AKO and BKO mice, scale bar = 200μm (images represent results from three animals per genotype); **(c)** Gain in body weight with age in AKO and BKO male and female mice, (n = 11 and 12 male of WT and AKO respectively, 10 and 6 female of WT and AKO respectively, n = 6 and 5 male of WT and BKO respectively, 6 and 7 female of WT and BKO respectively); **(d-e)** In-gel activity assay for **(d)** Complex I (CI) and **(e)** Complex V (CV) in WT, KO, AKO and BKO mice, (n = 3); **(f)** Quantification of lipolysis (glycerol release) in BAT of WT and KO mice under baseline (control) and stimulated conditions (n = 3); **(g)** Oil red O staining of WT and KO mBA cells (images represent results from three independent experiments). **(h)** Relative steady-state protein levels (log2) as obtained by label-free proteomics analysis of WT and KO preadipocytes and differentiated WT and KO mBA cells (n = 3); (**f** and **h**) Data are presented as mean ± SD. *p < 0.05, **p < 0.01, ***p < 0.001, ****p < 0.0001 as determined by One-way ANOVA with multiple comparisons. Source data

To study the role of mitochondria in the physiological remodelling of BAT, we used a mouse model with deletion of the mitochondrial protease CLPP (*Clpp*^−^^/^^−^, hereafter CLPP-KO), which is involved in OXPHOS complex synthesis and maintenance^25,29^. The loss of CLPP in BAT led to the accumulation of fewer but larger LDs, giving the tissue a pale appearance consistent with BAT whitening (Fig. 1a–c). This phenotype was intrinsic to BAT and not due to systemic CLPP deficiency, because similar whitening was observed in tissue-specific knockouts, including pan-adipose (CLPP-AKO: *Clpp*^*fl/fl*^; *Adipoq-cre*)^30^ and brown-fat-specific models (CLPP-BKO: *Clpp*^*fl/fl*^; *Ucp1-cre*)^31^ (Fig. 1a and Extended Data Fig. 1a,b). Although whole-body CLPP deletion resulted in significant weight loss, CLPP-AKO mice maintained normal body size, and CLPP-BKO mice exhibited only mild weight reductions that were more pronounced in males (Extended Data Fig. 1c). Although male and female mice exhibit naturally different weight trajectories as they age, our extensive experience with CLPP-deficient models suggests that these differences do not categorically impact study outcomes. Therefore, we combined data from both sexes in subsequent analyses, ensuring balanced representation of male and female mice across all experiments whenever feasible.

In addition to enlarged LDs, ultrastructural analysis revealed increased numbers of enlarged mitochondria in CLPP-KO BAT, some of which were visibly paler with fewer cristae, suggesting impaired OXPHOS function (Fig. 1b–c). Mitochondria isolated from CLPP-KO, CLPP-AKO and CLPP-BKO BAT exhibited reduced levels of respiratory complex I (CI) and CI-containing supercomplexes, accompanied by decreased CI activity (Fig. 1d and Extended Data Fig. 1d). Consistent with this, CLPP-KO BAT showed accumulation of F1 subassemblies of complex V (CV), which are essential for maintaining membrane potential when OXPHOS is compromised (Fig. 1e and Extended Data Fig. 1e).

Knockout tissue homogenates displayed reduced lipolysis, becoming significant after forskolin stimulation, suggesting that impaired lipolytic activation (glycerol release) could contribute to the whitening phenotype (Extended Data Fig. 1f). Despite these mitochondrial defects, oxygen-consumption rates (OCRs) in BAT lysates remained unchanged under both basal and stimulated conditions (Fig. 1f), probably owing to compensatory mitochondrial biogenesis (Fig. 1g). By contrast, thermoneutrality-induced whitening involves mitophagy^15^, whereas high-fat-diet-driven whitening causes LD accumulation without changes in mitochondrial content^16^. These findings point to distinct mitochondrial responses in different BAT whitening contexts and LD expansions.

To explore the underlying mechanisms, we established immortalized preadipocyte (preAD) cultures from CLPP wild-type and knockout BAT (Fig. 1h). Upon differentiation, these cells exhibited mature brown adipocyte (mBA) features, including multilocular LDs and oligomycin resistance, indicative of mitochondrial uncoupling (Fig. 1h–k and Extended Data Fig. 1g). Both wild-type and CLPP-KO mBAs strongly upregulated UCP1 (Extended Data Fig. 1h). Transcript levels of LD-associated proteins were markedly higher in CLPP-KO mBAs, including the ubiquitous marker PLIN2 (also known as ADRP) and the adipocyte-specific proteins PLIN1 and PLIN4 (Extended Data Fig. 1h).

OCR and extracellular acidification rate (ECAR) measurements in mBAs revealed minimal, if any, difference between wild-type and CLPP-KO cells (Fig. 1j,k). However, both basal OCR and ECAR were higher than those in preADs, reflecting increased metabolic activity upon differentiation. These results confirm that mBAs faithfully model adult brown adipocyte function in both wild-type and CLPP-deficient conditions.

### Multimodal analyses reveal tissue-autonomous BAT remodelling

**Fig. 2:**
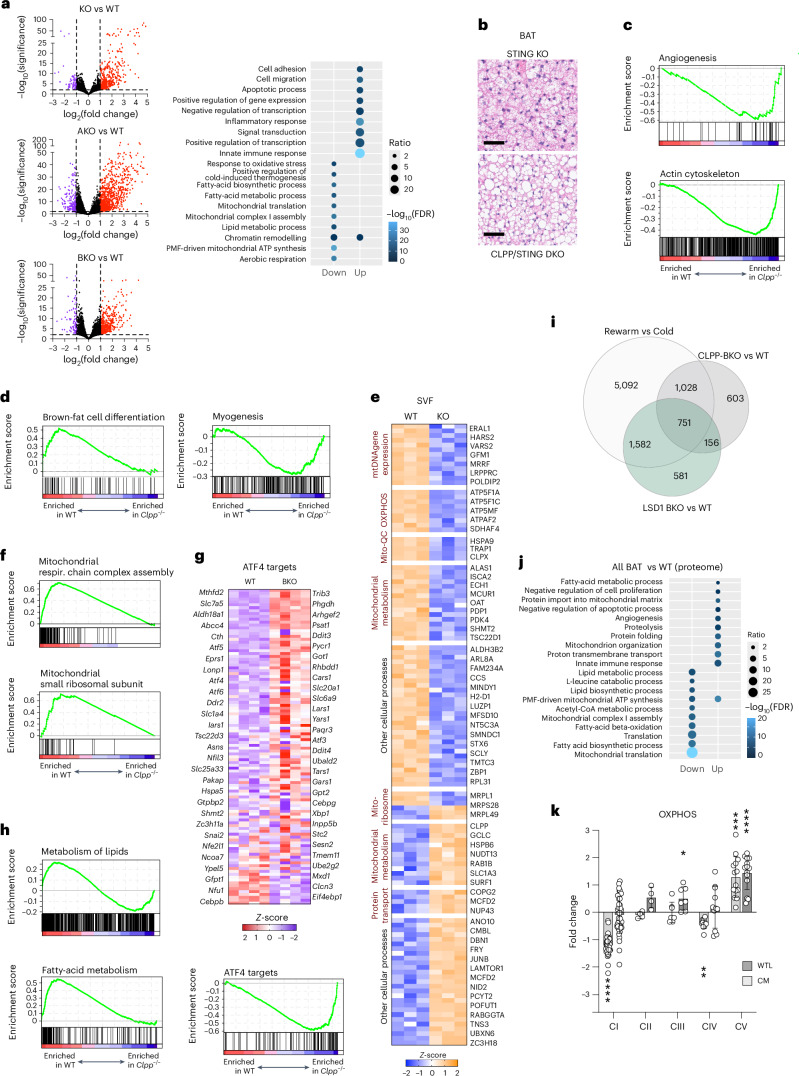
BAT is remodelled at multiple levels following CLPP loss. **a**, Left, volcano plots of differentially expressed genes in BAT isolated from CLPP-KO, CLPP-AKO or CLPP-BKO mice, compared with the WT (*n* = 4). Coloured dots show significantly changed transcripts (*P* ≤ 0,01, two-fold-change). Right, Gene Ontology: Biological Process (GO:BP) analysis of shared significantly changed transcripts (in RNA-seq analysis) in BAT from CLPP-KO, CLPP-AKO and CLPP-BKO mice (Supplementary Table 3). **b**, Representative H&E staining of BAT from STING-deficient (STING-KO) or STING- and CLPP-deficient (STING/CLPP DKO) mice. Scale bars, 200 μm (results obtained from three mice per genotype). **c**,**d**, GSEA enrichment plots for gene signatures related to angiogenesis and actin cytoskeleton (**c**) and brown-fat differentiation and myogenesis (**d**). **e**, Heat map with relative *Z*-scores of significantly changed proteins (*P* ≤ 0.05) in SVF from WT and CLPP-KO mice. **f**, GSEA enrichment plots for gene signatures related to mitochondrial function. **g**, Top, heat map showing relative *Z*-scores of ATF4-target genes in the transcriptome dataset from BAT tissue of CLPP-BKO mice compared with those in WT mice. Bottom, GSEA enrichment plots for ATF4-target gene signatures. **h**, GSEA enrichment plots for gene signatures related to lipid metabolism. **i**, Venn diagram depicting overlapping and unique changes in significantly changed transcripts (*P* ≤ 0.05) in CLPP-BKO BAT compared with changes in mice housed at 4 °C and those initially housed at 4 °C for 1 week and subsequently moved to thermoneutrality (30 °C) for 4 weeks (rewarm versus cold)^35^ and LSD1-KO mice^36^ (Supplementary Table 2). **j**, GO:BP analysis of common significantly changed BAT proteins (*P* ≤ 0.05) from CLPP-KO, CLPP-AKO and CLPP-BKO mice (Supplementary Table 3). **k**, Average changes of individual OXPHOS complexes isolated from CLPP-KO cytoplasmic mitochondria (CM) or the whole BAT tissue lysate (WTL). Each data point represents average fold change value (CLPP-KO/WT, *n* = 4) of individual OXPHOS subunits obtained from proteomics analyses (Supplementary Tables 3 and 4). The number of individual OXPHOS complex subunits identified in proteomics determines *n* (*n* = 40 (CI); *n* = 4 (CII); *n* = 7 (CIII); *n* = 11 (CIV); *n* = 14 (CV)). Data are presented as mean ± s.d. *P < 0.05, **P < 0.01, ***P < 0.001, ****P < 0.0001 as determined by paired two-tailed Student’s *t*-test. **a**,**j**, The size of the dots represents the number of genes and the colour of the dots represent the adjusted *P* values.

**Extended Data Fig. 2:**
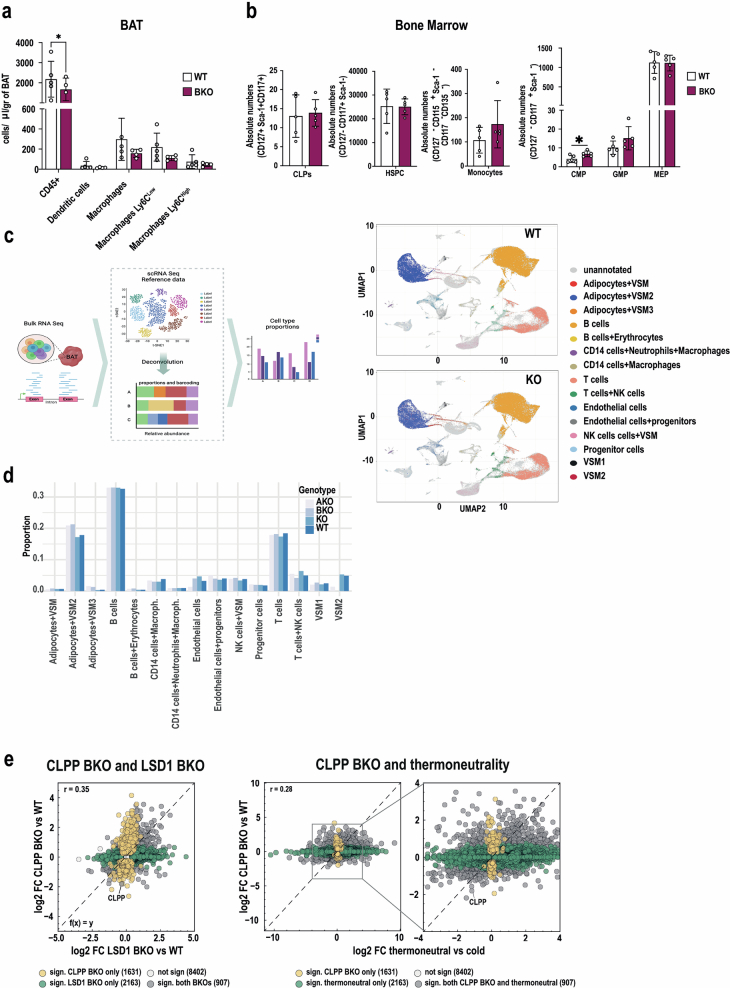
CLPP loss in BAT shows no immune or developmental changes. **(a)** Flow cytometry analysis of immune cells profile in the BAT from BAT specific CLPP KO (BKO) mice (n = 5); **(b)** Flow cytometry analysis of immune cells profile from bone marrow of BKO mice, graphs showing quantification of **(left** to **right)** lymphoid cells, hematopoietic progenitor cells, monocytes and mentioned progenitor cells (n = 5); **(c) (left)** Representative pipeline for deconvolution analysis followed; **(right)** Deconvoluted bulk RNA Seq data from BAT of ubiquitously-deficient CLPP mice (KO) mice, represented as UMAPs, with cell populations marked in different colours; **(d)** Relative proportions of designated cell types from deconvolution, reveals no quantitative differences in muscle cell population; **(e)** Comparison of BAT transcriptome from CLPP BKO to gene expression changes observed in BAT whitening models induced either by (left) BAT specific loss of LSD1 or (right) by thermoneutral temperatures. (**a** and **b**) Data are presented as mean ± SEM *p < 0.05, as determined by One-way ANOVA with multiple comparisons in **a** and **b**. Schematic created using Biorender.com.

To investigate the global impact of CLPP loss in BAT, we first analysed gene expression across all CLPP-deficient models (Fig. 2a). Gene Ontology (GO) enrichment using DAVID^32^ identified 707 commonly upregulated and 639 downregulated transcripts shared across genotypes (Supplementary Table 1). Inflammatory and immune pathways were among the most enriched (Fig. 2a and Supplementary Table 1), consistent with prior reports linking CLPP deficiency to cGAS–STING-mediated interferon-stimulated gene activation through mtDNA release^33^. However, STING deletion in CLPP-KO mice did not alter the BAT whitening phenotype (Fig. 2b), and we observed neither immune infiltration in CLPP-BKO BAT nor immune progenitor expansion in bone marrow (Extended Data Fig. 2a,b). Cell-type deconvolution based on single-cell transcriptomic references^34^ confirmed no major changes in cell composition, including immune populations (Extended Data Fig. 2c,d).

Beyond inflammation, CLPP-deficient BAT showed upregulation of pathways related to transcriptional regulation, signal transduction, adhesion, migration, cell death and lipid metabolism (Fig. 2a and Supplementary Table 1). Gene set enrichment analysis (GSEA) revealed enrichment of BAT-whitening-associated pathways such as actin remodelling and angiogenesis^13,14^ (Fig. 2a,c), whereas signatures of brown-adipocyte differentiation were negatively enriched and myogenesis appeared dysregulated (Fig. 2d), suggesting a possible impairment in BAT identity. Given the shared developmental origin of BAT and skeletal muscle, we tested whether differentiation was compromised by isolating the stromal vascular fraction (SVF) from wild-type and CLPP-KO BAT. Whole-proteome analysis of CLPP-BKO SVF revealed only minor changes, primarily in mitochondrial metabolism, mtDNA expression and OXPHOS proteins, with no alterations in markers of brown adipocytes or muscle differentiation (Fig. 2e), suggesting that early BAT development is intact.

Downregulated transcripts prominently included mitochondrial OXPHOS components and subunits of mitochondrial ribosomes (Fig. 2a,f), indicating organelle dysfunction. In agreement, the mitochondrial integrated stress response (mitoISR) was activated across models, as shown by GSEA and increased expression of ATF4 targets in CLPP-BKO BAT (Fig. 2g). Levels of transcripts involved in lipid metabolism, both catabolic (fatty acid oxidation) and anabolic (fatty acid and cholesterol synthesis), were also significantly reduced (Fig. 2a and Supplementary Table 1). Although GSEA confirmed overall suppression of lipid metabolic pathways, a subset of lipid-related genes was positively enriched, reflecting a dynamic remodelling process (Fig. 2h). This metabolic adaptation likely underlies LD accumulation, given that the OCR remained largely normal in knockout mBA cells (Fig. 1j), ruling out low OXPHOS activity as the cause.

We next compared CLPP-BKO transcriptomes with two BAT whitening models: thermoneutral exposure^35^ and lysine-specific demethylase 1 (LSD1) BAT-specific knockout (LSD1-BKO) mice^36^. Exposure to thermoneutrality more robustly altered BAT gene expression than did the loss of either CLPP or LSD1; in both of the latter models, less than quarter of alterations were unique (Fig. 2i). About 30% of the differentially expressed genes in CLPP-BKO mice overlapped with both whitening models (Fig. 2i and Extended Data Fig. 2e). GO terms shared across the three models matched those enriched in CLPP-deficient BAT (Supplementary Tables 1 and 2). Notably, *Clpp* expression was reduced in all three models, underscoring its link to BAT homeostasis (Extended Data Fig. 2e).

**Extended Data Fig. 3:**
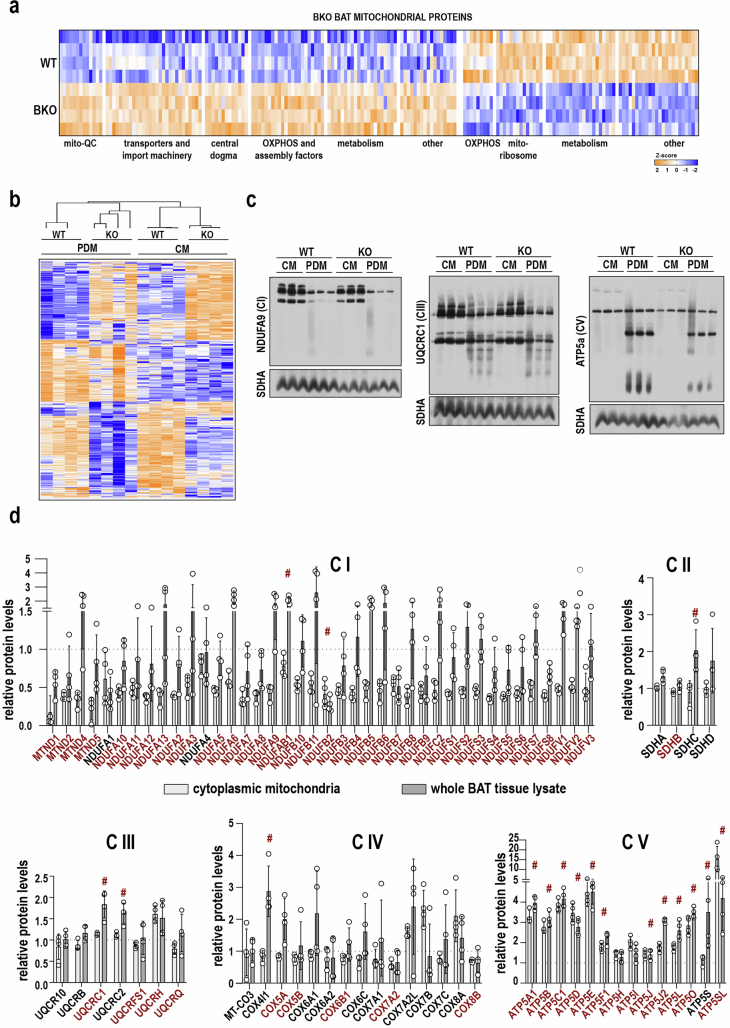
Isolated mitochondria from KO BAT have OXPHOS deficiency. **(a)** Heatmap of significantly changed protein abundances of mitochondrial proteins from BAT tissue of wild type (WT) and brown adipose specific CLPP deficient (BKO) mice. **(b)** Heat map of significantly changed protein abundances of mitochondrial proteins from isolated cytoplasmic and peri lipid droplet mitochondria of WT and ubiquitously-deficient CLPP mice (KO) mice, full table can be seen in Supplementary Table 4; **(c)** Steady-state levels of OXPHOS supercomplexes (SC) in BAT cytoplasmic (CM) or peri lipid droplet (PDM) mitochondria of WT and CLPP KO mice, analysed by BN-PAGE followed by western blot for **(left)** CI (NDUFA9) and **(middle)** CIII (UQCRC1) and **(right)** CV (ATP5A) (n = 3); **(d)** Relative fold changes of subunits of OXPHOS complexes in isolated CM from BAT and whole BAT tissue lysates, (n = 3 for CM and n = 4 for WTL). **(d)** Data are presented as mean ± SD. Red text colour represents complexes which were significantly changed in isolated cytoplasmic mitochondria and red text with # represents complexes significantly changed compared to whole BAT tissue lysates, as determined by paired two-tailed Student’s t-test in **d**. Source data

Although proteomic changes were less extensive than transcriptomic ones, with 116 upregulated and 66 downregulated proteins shared across CLPP-KO, CLPP-AKO and CLPP-BKO BAT, they largely mirrored transcript-level trends (Supplementary Table 3). GO analysis showed enrichment for mitochondrial proteins in both directions (Fig. 2j and Extended Data Fig. 3a). OXPHOS changes were modest, except for upregulated ATP synthase (complex V), consistent with a shift toward ATP production and reduced uncoupling, as seen in whitening BAT (Fig. 2k). Downregulated proteins clustered around fatty-acid-metabolism pathways, consistent with transcriptomic data (Fig. 2j).

To refine our analysis, we examined the proteomes of cytoplasmic mitochondria and peri-lipid droplet mitochondria (PDMs) from BAT^37^. These mitochondrial populations had distinct yet overlapping profiles, and both showed clear OXPHOS alterations under CLPP deficiency (Extended Data Fig. 3b,c). Notably, both cytoplasmic mitochondria and PDMs displayed pronounced complex I and milder complex IV deficiencies, similar to heart mitochondria in CLPP-KO mice (Fig. 2k and Extended Data Fig. 3d). These functional impairments were not apparent at the tissue level, likely owing to increased mitochondrial biogenesis (Fig. 2k).

Collectively, these findings suggest that BAT whitening in CLPP-deficient mice is not driven by impaired differentiation or adipocyte-to-myocyte conversion, nor is it a direct consequence of OXPHOS loss. Instead, a transcriptional signature of suppressed lipid metabolism likely underlies the expansion of lipid droplets. The broad reshaping of the BAT transcriptome, particularly enrichment of GO terms related to transcriptional control and chromatin regulation, points to an adaptive stress response to CLPP loss (Fig. 2a and Supplementary Table 1).

### CLPP loss causes d-2HG accumulation that dictates whitening

**Fig. 3:**
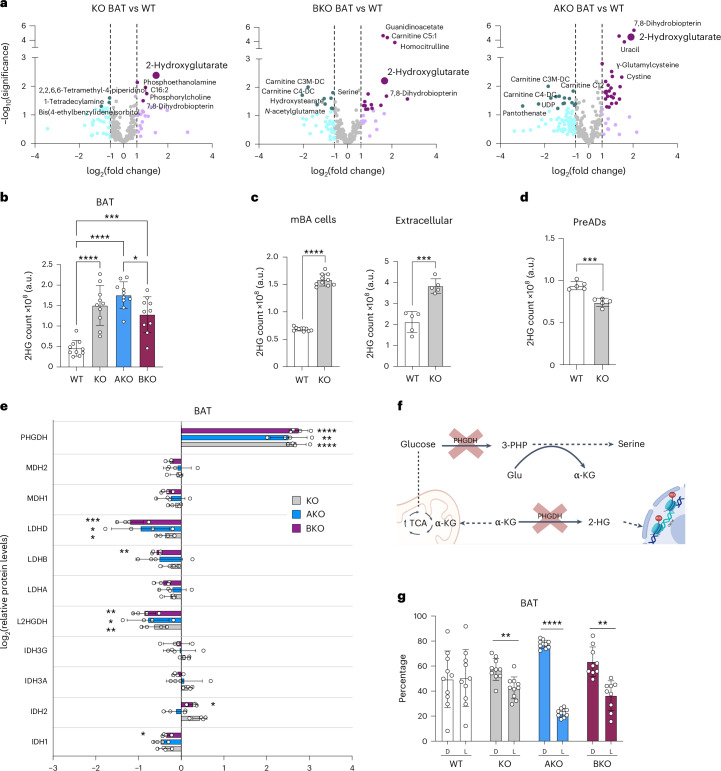
d-2HG accumulates in CLPP-deficient adipocytes. **a**, Volcano plots of metabolites with significantly changed levels in BAT isolated from ubiquitously-deficient (KO), adipose-specific (AKO) or BAT-specific (BKO) CLPP-KO mice, compared with the WT (*n* = 5); dark colored dots present significantly changed metabolites (*P* ≤ 0,01, two-fold-change) (as seen in Supplementary Table 5). **b**–**d**, 2HG levels in BAT tissue from WT and CLPP-KO, CLPP-AKO and CLPP-BKO mice (*n* = 10) (**b**); in WT and CLPP-KO mBA cells (left) and released in media by cultured cells (right), (*n* = 5) (**c**); and in WT and CLPP-KO preADs (*n* = 5). a.u., arbitrary units. **e**, Relative changes of proteins implicated in production of 2HG, measured in BAT tissue proteomics from CLPP-KO, CLPP-AKO and CLPP-BKO mice compared with levels in WT mice (*n* = 4). **f**, Schematic depicting specific roles of PHGDH and the inhibition by NCT503. TCA, tricarboxylic acid cycle. **g**, Relative percentages of the d and l isoforms of 2HG in WT, and CLPP-KO, CLPP-AKO and CLPP-BKO BAT tissue, as assessed by the derivatization of 2HG using TSCP (*n* = 5). **a**–**g**, Each dataset represents either a single mouse or single cell culture plate. **b**–**e**,**g**, Data are presented as mean ± s.d. *P < 0.05, **P < 0.01, ***P < 0.001, ****P < 0.0001, as determined by unpaired two-tailed Student’s *t*-test in **c**–**e** and **g** and one-way ANOVA with multiple comparisons in **b**. The schematic in **f** was created using Biorender.com. Source data

**Extended Data Fig. 4:**
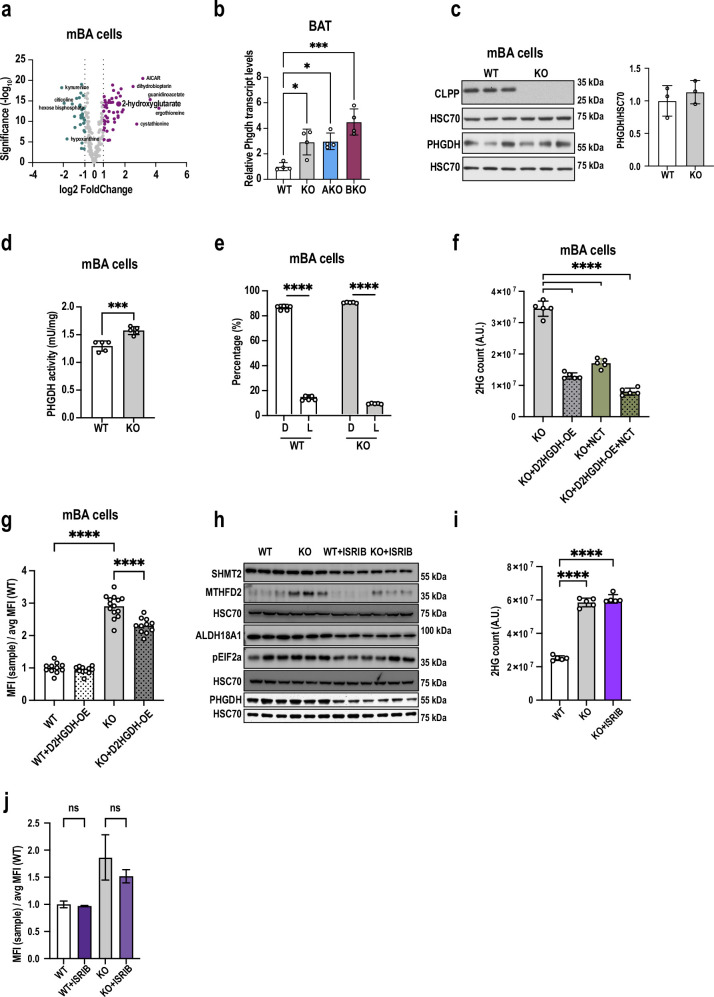
2-HG accumulates in BAT and is potentially produced by PHGDH. **(a)** Volcano plot of significantly changed metabolites in CLPP deficient (KO) mBA cells compared to wild type (WT) cells (n = 5), dark colored dots present significantly changed metabolites (p≤0,01, two-fold-change); **(b)** Relative transcript abundances of PHGDH in BAT from ubiquitously-deficient (KO), adipose-specific (AKO) or BAT specific (BKO) CLPP KO mice, compared to the wild type wild type (WT) (n = 4); **(c)** Immunoblot and quantification of steady state levels of PHGDH from WT and KO mBA cells (n = 3, western blot analysis was repeated with additional three independent samples/group); **(d)** Quantification of PHGDH enzymatic activity (n = 5) **(e)** Relative percentages of 2HG D and L isoforms in WT and CLPP KO mBA cells, as assessed by the derivatization of 2HG using TSCP (n = 5); **(f)** Quantification of 2HG by targeted metabolomics in KO, D2HGDH overexpressing KO (KO + D2HGDH), NCT503 treated KO (KO + NCT) and NCT503 treated KO + D2HGDH (KO + D2HGDH + NCT) mBA cells, (n = 5); **(g)** Lipid levels in wild type (WT), D2HGDH overexpressing WT (WT + D2HGDH), KO and KO + D2HGDH mBA cells, normalized to the average of the MFI value of the WT cells (n = 11,11,11,14 respectively); **(h)** Immunoblot of steady state levels of ISR proteins from wild type (WT), ISRIB treated WT (WT + ISRIB), KO and ISRIB-treated KO (KO + ISRIB) mBA cells (n = 3) (blots representative of three independent experiments); **(i)** Quantification of 2HG by targeted metabolomics from WT, KO and KO + ISRIB mBA cells (n = 5); **(j)** Lipid levels in WT, WT + ISRIB, KO and KO + ISRIB mBA cells, normalized to the average of the MFI value of the WT cells (n = 4). **(b-g, i-j)** Data are presented as mean ± SD. *p < 0.05, ***p < 0.001, ****p < 0.0001, as determined by One-way ANOVA with multiple comparisons in **b, e, f, h** and **i** and Students paired two tailed t-test in **d**; ^##^p < 0.001 represent significant difference in the ratio between D- and L-forms between WT and KO, as determined by Students paired two tailed t-test. Source data

Transcriptome and proteome analyses of CLPP-deficient BAT revealed extensive metabolic adaptations. To further examine this, we performed semi-targeted metabolomics across CLPP-deficient models. Despite there being relatively few metabolites with significantly altered levels, a consistent overlap was observed between models (Fig. 3a and Supplementary Table 5). In line with the larger lipid droplets, levels of long-chain fatty acids were elevated, and levels of carnitines derived from branched-chain amino acids were dysregulated (Supplementary Table 5). Among all changed metabolites, 2-hydroxyglutarate (2HG) was the most strongly upregulated, showing a 2.7- to 3.7-fold increase across all three models (Fig. 3a,b). CLPP-deficient mBA cells also accumulated and secreted 2HG, suggesting a potential paracrine role (Fig. 3c and Extended Data Fig. 4a), whereas KO preADs did not, indicating that 2HG accumulation is specific to mature brown adipocytes (Fig. 3d).

2HG, a structural analogue of α-ketoglutarate (α-KG), exists as two enantiomers, d- and l-2HG. It can be produced at low levels by malate dehydrogenase (MDH)^38^, lactate dehydrogenase A (LDHA)^39^, phosphoglycerate dehydrogenase (PHGDH)^40^ or mutant forms of isocitrate dehydrogenase (IDH1 and IDH2)^41^. To identify the source of 2HG, we analysed the expression of all known enzymes involved in its production^38–41^. PHGDH was the only one that was consistently upregulated, with more than fivefold increases across all CLPP-deficient models (Fig. 3e and Extended Data Fig. 4b). PHGDH catalyses the first step of the serine biosynthesis pathway, frequently induced as part of mitoISR in response to OXPHOS dysfunction (Fig. 3f)^6^. Although PHGDH protein was not increased in CLPP-KO mBA cells, its enzymatic activity was significantly increased (Extended Data Fig. 4c,d). Enantiomer-specific analysis confirmed that d-2HG is the predominant form in all CLPP-KO BAT samples, as well as in isolated mature CLPP-KO brown adipocytes (Fig. 3g and Extended Data Fig. 4e), aligning with known PHGDH promiscuity toward d-2HG production^40^. The difference in 2HG enantiomer proportions between wild-type BAT and wild-type mBA cells is likely due to the heterogeneous nature of BAT, which consists of a mix of diverse cell types^34^.

**Fig. 4:**
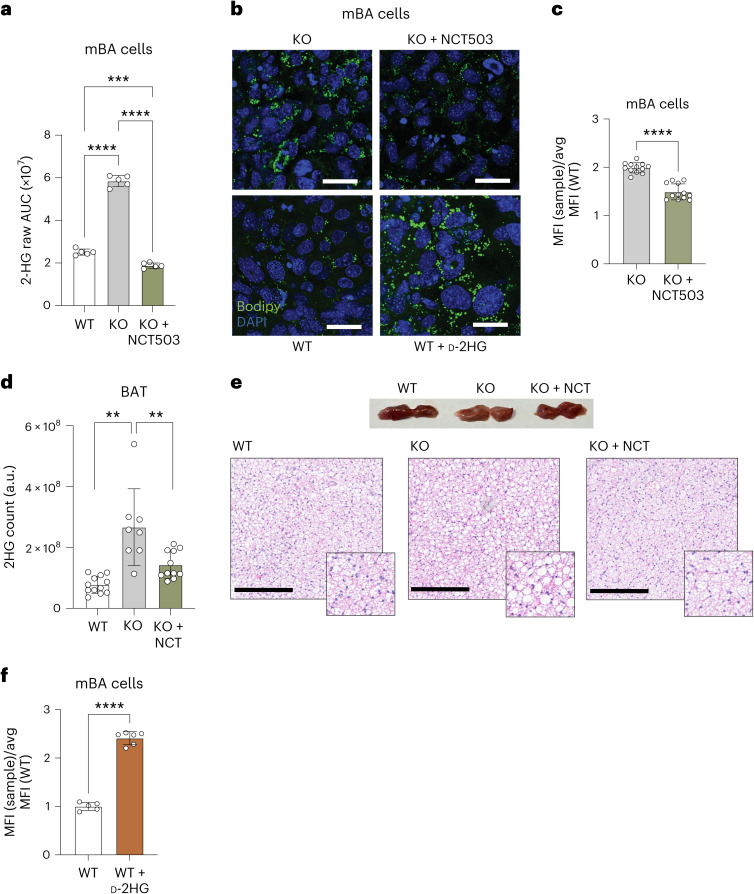
d-2HG accumulation in adipocytes upon CLPP loss causes whitening. **a**, 2HG levels as determined by targeted metabolomics in WT, CLPP-KO and NCT503-treated KO mBA cells (*n* = 5). **b**, Representative images of WT, CLPP-KO, d-2HG-treated WT and NCT503-treated CLPP-KO mBA cells, stained with DAPI (nucleus) and Bodipy (LDs). Scale bar, 100 μm (images represent results from four independent experiments). **c**, Lipid levels in NCT503-treated and untreated CLPP-KO mBA cells, normalized to the average MFI value of WT cells (*n* = 12). **d**, 2HG levels in WT and CLPP-KO BAT after 14 days of NCT503 treatment in vivo (*n* = 12 for WT, *n* = 7 for KO and *n* = 11 for KO + NCT). **e**, Top, representative images of whole BAT. Bottom, H&E staining of tissue from WT, CLPP-KO and NCT503-treated KO (KO + NCT) mice. Scale bars, 200 μm. Images represent results from four mice per condition. **f**, Lipid levels in d-2HG-treated and untreated WT mBA cells, normalized to the average MFI value of WT cells (*n* = 12). MFI quantification of Bodipy-stained 2HG-treated and untreated WT mBA cells, normalized to the average of the MFI value of the WT cells (*n* = 5 per condition). Individual data points represent either a single mouse or a single cell culture plate. Data are presented as mean ± s.d. **P < 0.01, ***P < 0.001, ****P < 0.0001, as determined by unpaired two-tailed Student’s *t*-test in **c** and **f** and one-way ANOVA with multiple comparisons in **a** and **d**.

To directly test the putative role of PHGDH in d-2HG production, we inhibited it using NCT503, a selective inhibitor that blocks serine synthesis^42^. NCT503 treatment abolished 2HG accumulation in knockout mBA cells (Fig. 4a and Supplementary Table 6) and broadly altered metabolic pathways, including one-carbon metabolism, the SAM cycle and purine biosynthesis (Supplementary Table 6). Notably, PHGDH inhibition also reversed the whitening phenotype: lipid content was reduced, and smaller LDs formed in KO mBA cells, confirmed by fluorescence-activated cell sorting (Fig. 4b,c). Furthermore, two weeks of in vivo treatment with NCT503 normalized 2HG levels and reversed BAT whitening in CLPP-KO mice (Fig. 4d,e). To further probe the role of d-2HG, we overexpressed d-2HG dehydrogenase (d-2HGDH), the enzyme responsible for its degradation, in CLPP-deficient mBA cells. This significantly reduced d-2HG levels and partially reversed the whitening phenotype (Extended Data Fig. 4f,g). By contrast, inhibition of the ISR using ISRIB suppressed some ATF4 targets but had no effect on d-2HG accumulation or lipid storage (Extended Data Fig. 4h–j), suggesting that mitoISR is not the main driver of whitening phenotype following CLPP deficiency. Finally, we treated wild-type mBA cells with cell-permeable d-2HG (octyl d-2HG). This was sufficient to induce LD enlargement (Fig. 4b,f), directly linking d-2HG accumulation with BAT whitening.

In summary, our data demonstrate that CLPP loss activates PHGDH, leading to d-2HG accumulation in mature brown adipocytes. This metabolite contributes to lipid-droplet expansion and BAT whitening, revealing a critical role for aberrant serine metabolism and d-2HG in mediating the phenotypic consequences of mitochondrial stress.

### CLPP loss and d-2HG treatment alter histone methylation

**Extended Data Fig. 5:**
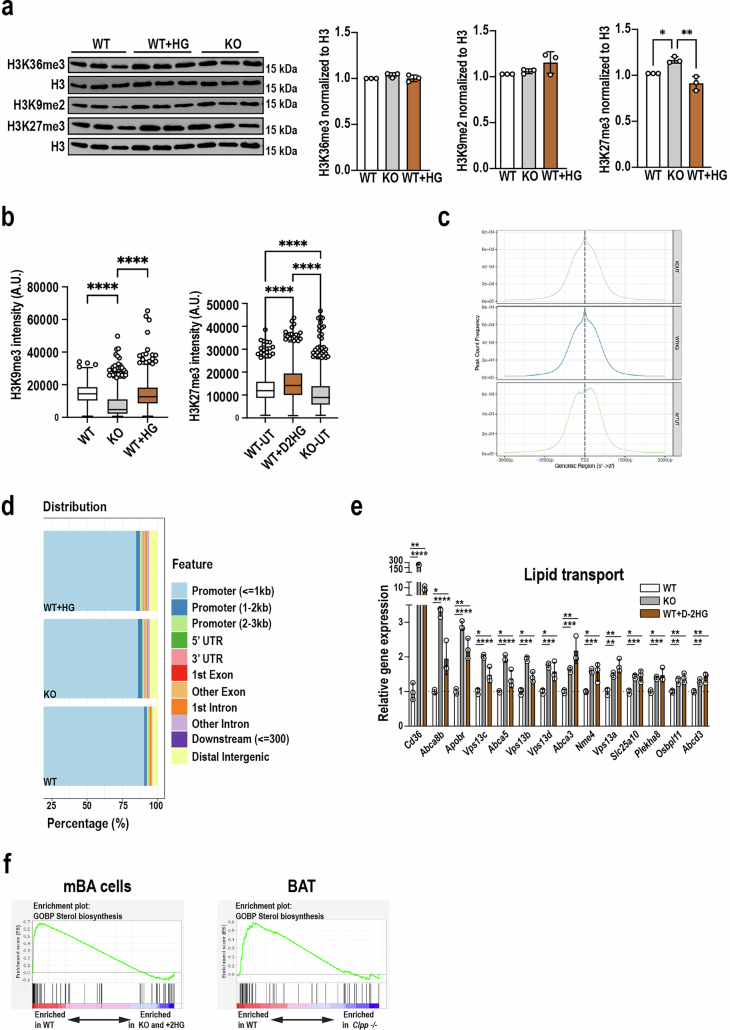
Omics analysis shows remodelling of mBA cells upon treatments. **(a)** Immunoblot of steady state levels of indicated histone modifications in wild type (WT), D-2HG treated WT (WT + HG) and CLPP deficient (KO) mBA cells, (right) quantification of levels of the histone marks normalized to H3 from the immunoblots, (n = 3); **(b)** Single nuclei intensity quantitation of histone marks in untreated WT, WT + HG and KO mBA (n = 798 for WT, n = 825 for WT + HG and n = 1088 for KO, collectively obtained from three individual cell culture plates); **(c)** Peak count frequency profile depicting enrichment of H3K4me3 peaks from CHIP Seq of WT, KO and WT + HG mBA cells relative to the TSS; **(d)** Feature distribution bar plot depicting relative proportions of genomic regions enriched in H3K4me3 in WT, KO and WT + HG mBA cells; **(e)** Relative mRNA levels of genes involved in lipid transport in CLPP KO cells. Untreated WT cells, and cells treated with D-2HG are used as controls (n = 3); **(f)** GSEA enrichment plots for gene signatures related to sterol biosynthesis in mBA cells and BAT tissue of KO mice compared to WT. **(a, e)** Data are presented as mean ± SD. **(b)** Data are presented using Tukey’s box plot with middle line marking the median, and whiskers show variability within 1.5×IQR. Anything beyond is an outlier presented as individual value. *p < 0.05, **p < 0.01, ***p < 0.001, ****p < 0.0001, as determined by One-way ANOVA with multiple comparisons in **a, b** and **e**. Source data

Elevated 2HG levels in cancer inhibit α-KG-dependent dioxygenases, affecting histone and DNA demethylation and leading to broad epigenetic reprogramming^43^. Initial western blot analyses of CLPP-deficient cells and wild-type cells treated with d-2HG did not reveal consistent changes in key histone modifications (histone H3 dimethylated at Lys9 (H3K9me2), H3 trimethylated at Lys36 (H3K36me3), H3K4me3, H3K27me3) (Extended Data Fig. 5a). Given the limited sensitivity of this method, subtle epigenetic shifts might have been missed. Immunofluorescence assays showed modest, inconsistent changes in H3K9me2 and H3K27me3) (Extended Data Fig. 5b), prompting a more focused analysis of H3K4me3, a mark of active and poised promoters known to be influenced by 2HG^43^.

**Fig. 5:**
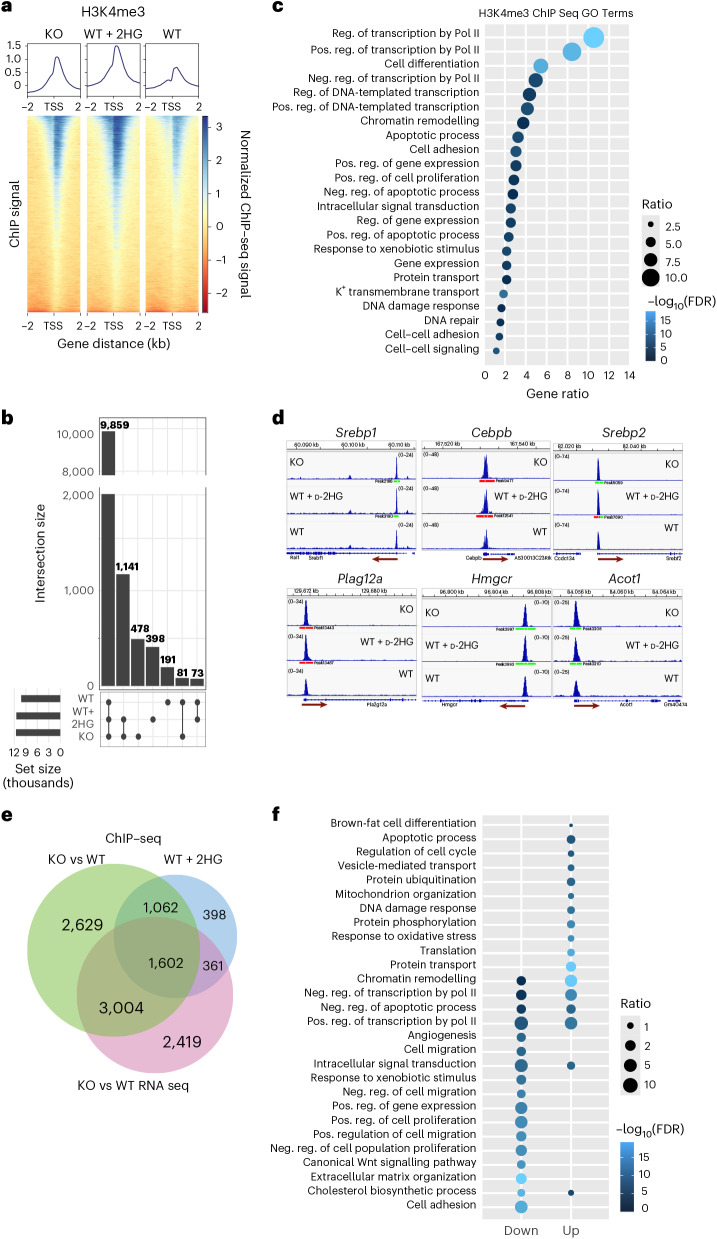
CLPP loss mirrors d-2HG-induced histone methylation. **a**, Heatmap and coverage plots of H3K4me3 ChIP–seq in WT, CLPP-KO and 2HG-treated WT mBA cells, ±2 kb around the transcription start site (TSS) (*n* = 4). **b**, Upset plot depicting the shared and unique H3K4me3 signatures between WT, CLPP-KO and d-2HG treated WT mBA cells (WT + 2HG). **c**, GO:BP analysis of H3K4me3 chromatin marks shared between KO and d-2HG-treated WT mBA cells (Supplementary Table 7). The size of the dots represent the number of genes, and the colour of the dots represent the adjusted *P* values (*n* = 4). Neg. reg., negative regulation; pos. reg., positive regulation; Pol II, RNA polymerase II. **d**, Integrative Genomics Viewer browser views showing H3K4me3 read density on the promoters of selected genes (*n* = 4). **e**, Venn diagram depicting the number of unique and overlapping changes between transcripts differentially expressed in CLPP-KO mice, compared with distinct H3K4me3 signatures found in KO and d-2HG-treated WT mBA cells. **f**, GO:BP analysis of common significantly changed transcripts between CLPP-KO and d-2HG-treated WT mBA cells (Supplementary Table 8). The size of the dots represent the number of genes and the colour of the dots represent the adjusted *P* values (*n* = 4). Source data

Chromatin immunoprecipitation followed by high-throughput sequencing (ChIP–seq) revealed a global increase in H3K4me3 in both CLPP-deficient and d-2HG-treated mBA cells, with most enrichment occurring at promoter regions (Fig. 5a and Extended Data Fig. 5c,d). Notably, 1,141 out of 2,017 unique H3K4me3 peaks in CLPP-deficient cells overlapped with those in d-2HG-treated cells, suggesting a shared mechanism (Fig. 5b and Supplementary Table 7). GO analysis of these enriched loci shared between CLPP-deficient and d-2HG-treated mBA cells revealed biological processes associated with transcriptional regulation, differentiation, chromatin reorganization, lipid metabolism, apoptosis and DNA repair (Fig. 5c and Supplementary Table 7). Many of these categories also emerged in transcriptomic analyses of CLPP-deficient BAT (Supplementary Table 1), supporting their functional relevance.

A closer examination of individual genes identified increased H3K4me3 at promoters of key adipogenic regulators, including SREBP1 and SREBP2 (cholesterol and fatty acid metabolism), and C/EBPβ (white adipocyte differentiation)^44,45^ (Fig. 5d). These changes suggest that d-2HG-induced chromatin remodelling could promote transcriptional programmes linked to adipogenesis and metabolism. To further explore this, we compared transcriptomes of CLPP-KO and d-2HG-treated mBA cells. More than 50% of significantly altered transcripts in CLPP-KO cells exhibited corresponding changes in H3K4me3 levels (Fig. 5e and Supplementary Tables 7 and 8). Additionally, more than one-third of these were shared with d-2HG-treated cells, implicating 2HG as a major driver of transcriptional changes in CLPP deficiency.

GO analysis revealed enrichment of both up- and downregulated transcripts in pathways related to chromatin remodelling, transcriptional and translational regulation and apoptosis, indicating broad alterations in these processes (Fig. 5f and Supplementary Table 8). Distinct trends were also evident: oxidative stress and DNA-damage-response terms were enriched among upregulated genes, whereas cell adhesion and extracellular matrix organization terms were downregulated in both models (Fig. 5f and Supplementary Table 8).

**Fig. 6:**
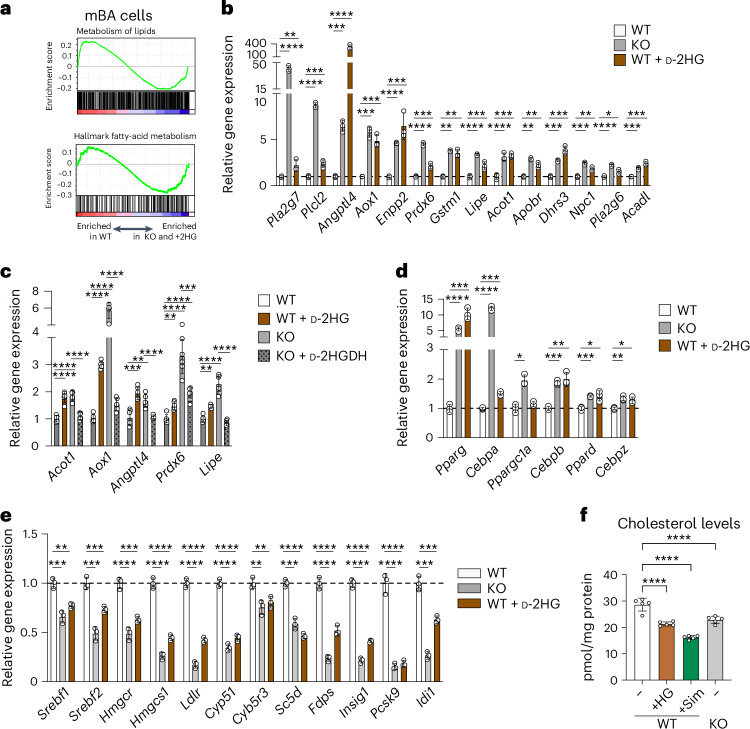
CLPP loss and d-2HG reshape lipid metabolic pathways. **a**, GSEA enrichment plots for gene signatures related to lipid metabolism shared between CLPP-KO cells and WT mBA cells treated with d-2HG. **b**, Relative mRNA levels of genes under GO:BP analysis term ‘Lipid metabolism’ enriched in CLPP-KO cells and WT cells treated with d-2HG, obtained from RNA sequencing (RNA-seq) analysis (*n* = 4) (Supplementary Table 8). **c**, Relative mRNA levels of genes involved in lipid metabolism in CLPP-KO cells upon expression of d-2HGDH, as measured by quantitative real-time PCR. Untreated WT cells, and cells treated with d-2HG, were used as controls (*n* = 6). **d**, Relative mRNA levels of major regulators of lipogenesis genes enriched in CLPP-KO cells and WT cells treated with d-2HG obtained from RNA-seq analysis (*n* = 4) (Supplementary Table 8). **e**, Relative mRNA levels of genes under GO:BP analysis term ‘Cholesterol metabolism’ enriched in CLPP-KO cells and WT cells treated with d-2HG, obtained from RNA-seq analysis (*n* = 4) (Supplementary Table 8). **f**, Cholesterol levels in untreated WT cells, CLPP-KO mBA cells or WT cells treated with either d-2HG or simvastatin (Sim) (*n* = 5). **b**–**f**, Data are presented as mean ± s.d. *P < 0.05, **P < 0.01, ***P < 0.001, ****P < 0.0001, as determined by one-way ANOVA with multiple comparisons.

GSEA analysis identified substantial changes of lipid and fatty acid metabolism, mirroring alterations observed in CLPP-deficient tissues (Figs. 2h and 6a). Among the transcripts upregulated in both CLPP-KO mBA cells and cells treated with d-2HG, we identified multiple enzymes involved in lipid metabolism, along with a variety of lipid transporters, including the highly upregulated CD36, a major regulator of fatty acid uptake in adipocytes and a key factor in LD growth^46^ (Fig. 6b and Extended Data Fig. 5e). Many of these transcripts were suppressed in cells overexpressing d-2HGDH (Fig. 6c). In parallel, transcripts encoding adipogenic regulators, such as *Pparg* and *Cebpb*, were upregulated in both CLPP-KO mBA cells and cells treated with d-2HG. By contrast, *Ppargc1a*, encoding PGC1α, was specifically induced in CLPP-deficient cells, consistent with underlying mitochondrial OXPHOS dysfunction (Fig. 6d). Remarkably, *Cebpa* expression appeared to be similarly influenced by mitochondrial dysfunction rather than d-2HG treatment (Fig. 6d). Conversely, cholesterol-biosynthesis genes were negatively enriched in both CLPP-deficient and d-2HG-treated cells; this change was also observed in BAT from all three CLPP-KO models (Extended Data Fig. 5f), highlighting a strong similarity between cells and tissues. Transcripts encoding SREBP1 and 2 (*Srebf1*, *Srebf2*) and their targets, including *Hmgcr* and *Hmgcs1*, which encode key catalysts of the initial rate-limiting steps of cholesterol biosynthesis (Fig. 6e). These changes coincided with reduced cellular cholesterol levels in both CLPP-deficient and d-2HG-treated cells, similar to the effect of simvastatin, an inhibitor of cholesterol biosynthesis (Fig. 6f).

Together, these findings demonstrate that CLPP deficiency and d-2HG treatment induce similar changes in transcriptome and epigenome, that converge on chromatin regulation, adipogenic signalling and cholesterol metabolism, linking mitochondrial dysfunction to coordinated metabolic reprogramming.

### d-2HG mediates nuclear softening in CLPP-KO brown adipocytes

**Extended Data Fig. 6:**
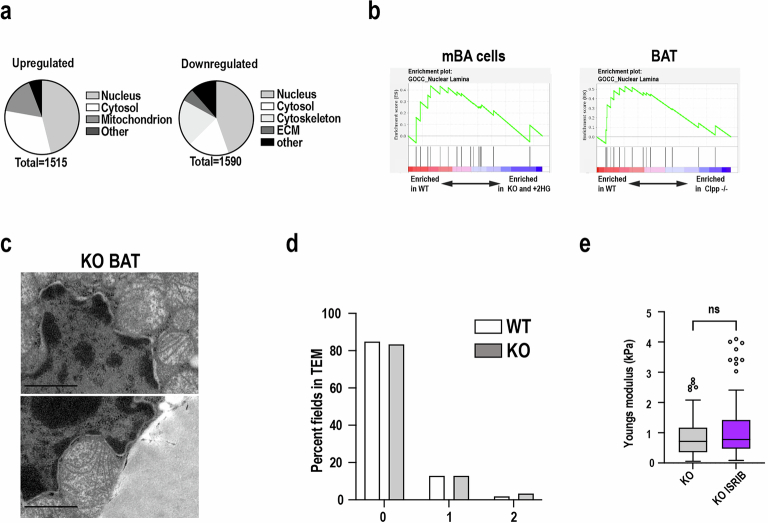
Nuclear architecture changes upon CLPP loss in BAT. **(a)** Venn diagram representing GO:CC analysis of common differentially expressed genes in CLPP deficient (KO) and 2HG treated WT (WT + HG) cells compared to untreated wild type (WT) mBA cells; **(b)** GSEA enrichment plots for gene signatures related to nuclear lamina in both KO mBA cells and KO BAT tissue compared to WT; **(c)** Representative TEM images showing mitochondria impinging on the nucleus and juxtaposed to nuclear pores (images represent results observed in 19 TEM sections from KO BAT of three animals); **(d)** Quantitation of the number of ER sites spotted in TEM images from WT and KO mice, represented as percentage of the total number of fields assessed; **(e)** Quantification of Young’s modulus in KO and ISRIB-treated KO mBA nuclei (n = 110 for KO, n = 120 for KO + ISRIB and n = 1088 for KO, collectively obtained from three individual cell culture plates). **(e)** Data are presented using Tukey’s box plot with middle line marking the median, and whiskers show variability within 1.5×IQR. Anything beyond is an outlier presented as individual value. No significant differences were observed as assessed by paired two-tailed Student’s t-test.

GO enrichment analysis of transcriptional and H3K4me3 ChIP–seq changes in CLPP-deficient tissues and cells consistently identified terms related to cell adhesion, migration, actin cytoskeleton organization and transcriptional regulation (Figs. 2a and 5c,f and Supplementary Tables 1, 7 and 8). A major number of enriched transcripts mapped to the nucleus, nucleoplasm and perinuclear cytoplasm. This aligns with emerging evidence linking nuclear architecture to cellular stress responses. The overlap of nucleus-related transcripts between CLPP-KO and d-2HG-treated cells supports this connection (Extended Data Fig. 6a). GSEA further revealed strong negative enrichment of nuclear envelope-associated transcripts in CLPP-deficient BAT, closely matching the transcriptomic signature of d-2HG-treated cells (Extended Data Fig. 6b), suggesting that metabolic perturbations could influence nuclear and cellular morphology.

**Fig. 7:**
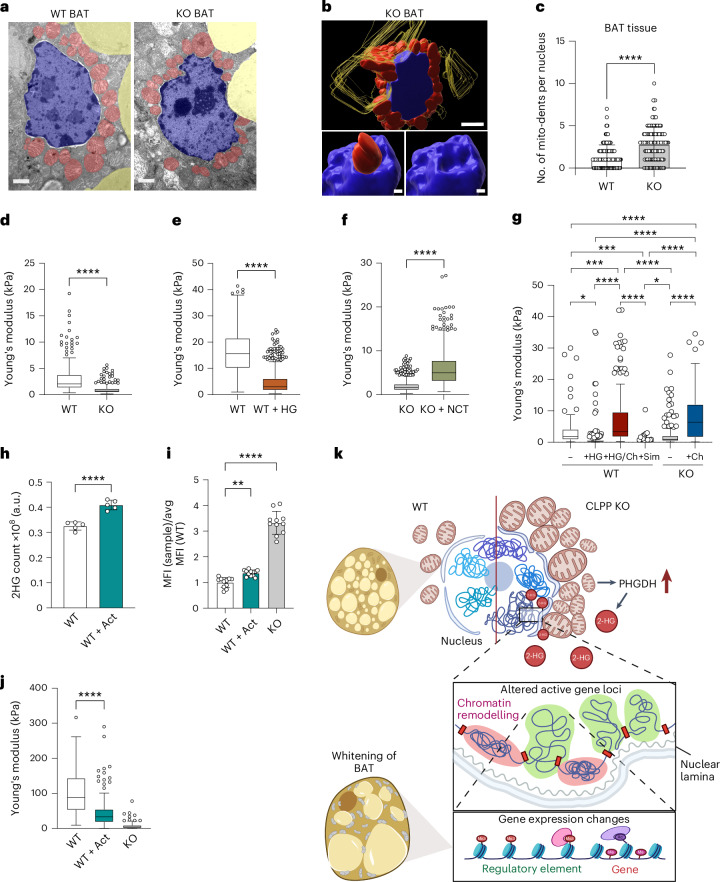
2HG mediates nuclear softening in CLPP-deficient adipocytes. **a**, Representative TEM image from BAT of WT and CLPP-KO mice. LDs (yellow), nucleus (blue) and mitochondria (red) (*n* = 3 mice). Scale bars, 2 μm. **b**, 3D reconstruction of serial TEM images from KO BAT tissue (Supplementary Video 1). Scale bars, 1 μm. **c**, Quantification of nuclear dents in mitochondria (*n* = 176 fields for WT and *n* = 150 for KO, from two mice per group). **d**–**g**, Quantification of Young’s modulus from AFM-mediated force spectroscopy of mBA nuclei from: WT and CLPP-KO (*n* = 269 for WT and *n* = 260 for KO, each from four individual cell culture plates) (**d**); untreated WT or d-2HG-treated WT (WT + HG) (*n* = 772 for WT *n* = 642 for WT + HG, each from four cell culture plates) (**e**); CLPP-KO, either untreated (KO) or treated with NCT503 (KO + NCT) (*n* = 1,006 for KO, n = 735 for KO + NCT, each from four cell culture plates) (**f**); WT, CLPP-KO and WT treated with d-2HG, supplemented with cholesterol (Ch) (**g**). Untreated WT simvastatin (Sim)-treated cells were used as controls (*n* = 80 for WT, *n* = 169 for WT + HG, *n* = 143 for WT + HG+chl n = 151 for sim, *n* = 177 for KO and *n* = 171 for KO+chl, from three cell culture plates). **h**, 2HG levels as determined by targeted metabolomics in WT and actinonin-treated (WT + Act) mBA cells (*n* = 5). **i**, Lipid levels in WT, actinonin-treated WT cells and CLPP-KO mBAs, normalized to the average MFI value of WT cells (*n* = 11). **j**, Quantification of Young’s modulus of nucleus of WT mBAs, CLPP-KO and WT cells treated with actinonin (WT + Act) (*n* = 107 for WT, *n* = 158 for WT + Act and *n* = 115 for KO, from three cell culture plates). **k**, Schematic summarizing the major findings of the study. **c**,**h**,**i**, Data are presented as mean ± s.d. **d**–**g**,**j**, Data are presented using Tukey’s box plot with middle line marking the median, and whiskers show variability within 1.5×IQR. Anything beyond is an outlier presented as individual value. *P < 0.05, **P < 0.01, ***P < 0.001, ****P < 0.0001, as determined by unpaired two-tailed Student’s *t*-test in **c**,**h** and **i** and Kolmogorov–Smirnov test in **d**–**g** and **j**. The schematic in **k** was created using Biorender.com.

We next explored the link between d-2HG, epigenetic changes and nuclear phenotype. Ultrastructural analyses revealed pronounced nuclear deformation in CLPP-deficient brown adipocytes, characterized by mitochondria clustering near and indenting the nuclear envelope (Fig. 7a). The extent of mito-nuclear interactions in CLPP-deficient BAT could be further observed in a high-resolution three-dimensional (3D) reconstituted model using serial section array tomography (Fig. 7b and Supplementary Video 1). Although direct contact sites with shared membranes were not detected, the two membranes were closely positioned with very little intercalated ER (Extended Data Fig. 6c,d and Supplementary Video 2).

Quantification of mito-nuclear indentations revealed an increase from ~1.3 per nucleus in wild-type to ~2.9 in CLPP-deficient cells, with some nuclei displaying up to 10 invaginations (Fig. 7c), indicating that nuclear mechanics were altered. Using atomic force microscopy (AFM), we detected a significant reduction in nuclear stiffness in CLPP-deficient adipocytes, mimicked by d-2HG treatment of wild-type cells (Fig. 7d,e). Inhibition of PHGDH with NCT503, which lowers d-2HG levels, restored nuclear stiffness in CLPP-deficient cells (Fig. 7f). By contrast, ISRIB treatment did not significantly change d-2HG levels and had no effect on nuclear envelope stiffness (Extended Data Fig. 6e). These findings establish a functional link between altered metabolism and nuclear morphology, regulated by d-2HG, but independent of the classical ISR.

Cholesterol increases membrane stiffness, especially in saturated lipid environments, with a weaker effect in unsaturated lipid-rich membranes^47^. Although the nuclear envelope is mainly composed of unsaturated phospholipids, increased cholesterol synthesis has been linked to nuclear envelope stiffening and fragility^48^. Because CLPP-deficient and d-2HG-treated cells showed reduced cholesterol levels, we tested whether supplementation could restore nuclear stiffness. Cholesterol treatment significantly increased nuclear stiffness in both models, exceeding levels observed in wild-type cells (Fig. 7g). Conversely, treatment with simvastatin, a cholesterol-synthesis inhibitor, further decreased nuclear stiffness (Fig. 7g), highlighting cholesterol as a critical regulator of nuclear envelope mechanics.

To test whether these effects extend beyond CLPP deficiency, we treated wild-type mBA cells with actinonin, an inhibitor of OXPHOS biogenesis^49^. Short-term actinonin treatment significantly increased 2HG levels in wild-type mBA cells and modestly elevated lipid accumulation (Fig. 7h,i), suggesting that metabolic reprogramming occurs rapidly upon mitochondrial dysfunction. This was accompanied by a more than twofold reduction in nuclear stiffness (Fig. 7j), suggesting that OXPHOS impairment broadly influences nuclear mechanics through rapid metabolic rewiring.

Collectively, our findings reveal that mitochondrial dysfunction drives metabolic reprogramming that reshapes nuclear architecture and mechanics. We propose that PHGDH activation and subsequent d-2HG accumulation induce nuclear softening, which is reversible and cholesterol-sensitive (Fig. 7k). Although our study centres on BAT whitening and CLPP loss, similar metabolic–nuclear coupling is likely to occur in other contexts of OXPHOS dysfunction and lipid accumulation, warranting further investigation.

## Discussion

This study identifies a mechanism by which CLPP loss and resulting OXPHOS dysfunction drive LD expansion, a key feature of BAT whitening, through metabolic and epigenetic adaptation. LD enlargement and BAT whitening are observed in various models of mitochondrial dysfunction and are often linked to respiratory-chain deficiencies^22–24^. Although impaired fatty acid oxidation (FAO) is frequently proposed as a cause, direct mechanistic evidence is limited, as seen in models like adipocyte-specific TFAM deletion^24^. Other studies, such as the one conducted on *Mfn2*-deficient adipocytes, suggest that reduced lipolysis, rather than increased lipogenesis, underlies LD accumulation^23^. However, not all OXPHOS deficiencies result in larger LDs. For instance, mtDNA mutator mice exhibit BAT dysfunction without LD expansion, likely due to systemic metabolic constraints and a catabolic state that limits lipid storage^50^.

Interestingly, BAT-specific OPA1 loss leads to LD accumulation and reduced oxygen consumption but paradoxically enhances thermogenesis through FGF21 induction^22^. Conversely, OPA1 overexpression protects against diet-induced obesity by preventing BAT whitening and stimulating WAT browning, potentially through fumarate-driven activation of KDM3A^51^. These findings underscore the complexity of mitochondrial stress responses and their influence on adipocyte fate through metabolic–epigenetic cross-talk.

Our data suggest that LD expansion and BAT whitening in CLPP-deficient models are a stress response to mitochondrial dysfunction, rather than a direct consequence of respiratory failure. This phenotype was consistent across in vivo models and terminally differentiated CLPP-KO brown adipocytes. Although LDs are typically considered energy reservoirs, they also serve protective roles, buffering oxidative stress, mitigating endoplasmic-reticulum stress and sequestering harmful lipid species^52^. LD expansion can limit polyunsaturated fatty acid oxidation and reduce lipotoxicity, which is particularly important under mitochondrial dysfunction, during which acylcarnitines, ceramides and oxidized lipids can accumulate^53^. Thus, we propose that LD biogenesis acts as an adaptive mechanism to maintain redox balance and prevent metabolic toxicity^52,53^.

We further show that LD expansion is driven by increased d-2HG levels, produced through promiscuous activity of PHGDH^40^ in CLPP-deficient adipocytes. Exogenous d-2HG recapitulates the phenotype in wild-type cells, and transcriptomic analysis reveals shared signatures affecting lipid and cholesterol metabolism. PHGDH, a key enzyme in serine biosynthesis and one-carbon metabolism, is frequently upregulated in mitochondrial disease models^5–7,54^. Given its central role in the mitoISR, inhibiting PHGDH in mitochondrial disease models, such as Deletor mice, is detrimental^54^. Our findings suggest that PHGDH-derived d-2HG has an additional role in modulating lipid metabolism and chromatin architecture.

Although d-2HG is best known for its role in cancer, it also functions in hypoxia, immune-cell differentiation and metabolic disorders^55^. In tumours, it inhibits α-KG-dependent dioxygenases, such as TET2 and Jumonji-C demethylases, thereby altering DNA and histone methylation^55^. These changes can shift cell fate and are associated with increased repressive marks, such as H3K9me3 and H3K27me3, without widespread changes in DNA methylation^56^. d-2HG also exerts non-cell-autonomous effects; for example, tumour-derived d-2HG suppresses CD8⁺ T cell metabolism and function^57^. In accordance with this, we observed 2HG secretion from mature brown adipocytes, raising the possibility of systemic effects in CLPP-deficient animals.

Our findings reveal new functions for d-2HG, demonstrating its role in regulating nuclear mechanics in terminally differentiated cells. Both CLPP deficiency and d-2HG treatment led to nuclear softening, associated with increased H3K4me3 occupancy at promoter regions. This included lipid metabolic regulators involved in lipogenesis, lipolysis and transport, consistent with LD expansion. Notably, although H3K4me3 was enriched at the promoters of cholesterol-biosynthesis genes, their expression and total cholesterol levels were reduced. This indicates that there are additional regulatory layers influenced by d-2HG.

Cholesterol is essential for membrane rigidity, including at the nuclear envelope. We show that cholesterol depletion causes nuclear envelope softening, whereas supplementation restores nuclear stiffness in CLPP-deficient and d-2HG-treated cells. This aligns with prior work showing that staurosporine-induced mitochondrial dysfunction alters mitochondria–nucleus interactions and cholesterol distribution^58^. Similarly, in yeast, oxygen deprivation enhances the impact of saturated lipids, leading to nuclear envelope rigidification and rupture, while revealing a protective role for LDs in buffering membrane composition and preserving nuclear envelope integrity^59^. Our data suggest that, in brown adipocytes, LD expansion might serve a similar protective role for the nuclear envelope.

Recent studies have shown that, under mechanical confinement, mitochondria migrate toward the nucleus, resulting in increased nuclear ATP to support chromatin reorganization and DNA repair^60^. Nuclear stiffness depends on both the nuclear lamina and heterochromatin, which provides structural support and mechanical resistance^61^. Cells sense deformation through nuclear envelope stretching, which triggers membrane phospholipid restructuring and actomyosin cytoskeleton changes^61^. Loss of heterochromatin has been linked to nuclear envelope softening and nuclear wrinkling^61,62^. In CLPP-deficient models and upon d-2HG treatment, we consistently observed changes in transcripts and proteins related to actomyosin cytoskeletal organization and cell adhesion and migration, consistent with responses seen under mechanical confinement^60,62^.

In conclusion, our study links mitochondrial dysfunction to nuclear reshaping, showing how CLPP loss and d-2HG-driven metabolic rewiring affect gene expression and nuclear structure to promote lipid-droplet accumulation. We identified d-2HG as a key mediator in this process, accumulating in CLPP-deficient BAT owing to increased PHGDH activity. This metabolite reshapes the epigenetic landscape by enriching H3K4me3 histone marks on promoters of lipid-metabolism regulators, orchestrating a shift toward LD accumulation, likely as a protective sink for potentially lipotoxic intermediates. Additionally, our findings highlight an unexpected interplay between cholesterol metabolism, cytoskeletal organization and nuclear mechanics, showing that changes in cholesterol availability contribute to nuclear softening. Although our focus is on brown adipocytes, these findings could extend broadly to tissues undergoing mitochondrial stress, with relevance to ageing, metabolic disease and cancer. By connecting mitochondrial dysfunction, chromatin reorganization, lipid metabolism and nuclear mechanics, our study provides a framework for understanding adaptive tissue remodelling in mitochondrial disorders.

## Methods

### Animal care and breeding

All mice analysed in this study were bred and housed at the CECAD Research Center animal facility, University of Cologne. All experiments were conducted in compliance with National Institutes of Health guidelines and approved by local government authorities (Landesamt für Natur, Umwelt und Verbraucherschutz Nordrhein-Westfalen; LANUV). Mice were housed in individually ventilated cages at 22–24 °C under a 12-h light–dark cycle, with lights on at 06:00. They had ad libitum access to water and a standard chow diet (ssniff rat/mouse low-phytoestrogen diet). For breeding, one male was paired with one to two females of similar age, with mating initiated at a minimum age of 8 weeks. Body weight was recorded weekly for individual mice from 4 to 16 weeks of age.

The mice used in this study were derived from a line with a conditional *Clpp* gene targeting, as previously described^29^. To generate whole-body CLPP-KO mice (*Clpp*^−*/*−^), *Clpp*^*fl/fl*^ mice were crossed with transgenic mice ubiquitously expressing Cre recombinase under the control of the β-actin promoter. Mice with adipose-tissue-specific knockout were generated by crossing *Clpp*^*fl/fl*^ mice with transgenic mice expressing Cre recombinase under the control of the *Adipoq* promoter (*Adipoq-cre* mice)^30^ or the *Ucp1* promoter (*Ucp1-cre* mice)^31^.

### In vivo NCT injections in mice

Each day for 15 days, 14-month-old male mice were intraperitoneally injected daily with vehicle (ethanol (5%), PEG400 (35%) and 30% cyclodextrin (60%)), in which NCT503 was dissolved. NCT-503 was freshly prepared prior to the injections. For the injection solution, 20 μM stock was dissolved in the vehicle and was administered to the mice at serum-stable concentrations of 40 mg kg^−1^ body weight^42^.

### Tissue histology and haematoxylin and eosin staining

After dissection, the entire adipose tissues were placed in tissue cassettes (Roth) and fixed in 5% (wt/vol) paraformaldehyde overnight at 4 °C. Tissues were then stored in PBS until they sank to the bottom. The next day, they were dehydrated in increasing ethanol concentrations (30%, 50%, 70%, 96% and twice in 100%) for 2 h at each step, followed by two 2-h incubations in xylol. Finally, tissues were embedded in paraffin blocks. The fixed BAT and inguinal WAT (iWAT) were sectioned at 5-μm thickness using a microtome (Leica) and mounted on poly-l-lysine-coated glass slides (VWR). Paraffin-embedded BAT sections were deparaffinized in xylol and rehydrated through decreasing ethanol concentrations, followed by a wash in tap water. Sections were stained with Meyer’s haematoxylin, rinsed and then counterstained with eosin. Sections were then dehydrated in increasing ethanol concentrations, cleared in xylol and mounted with entellan.

### Electron microscopy and 3D reconstruction of adipose tissue

#### Sample preparation and embedding

Adipose tissue samples were immersion-fixed in 2% formaldehyde and 2% glutaraldehyde in 0.1 M sodium cacodylate buffer (Applichem) and washed four times in the same buffer. Post-fixation was performed with 2% OsO₄ (Science Services) in 0.1 M cacodylate buffer at 4 °C, followed by additional washes. Samples were dehydrated in an ascending ethanol series (50%, 70%, 90%, 3×100%), treated with ethanol and propylene oxide and infiltrated with epon and propylenoxide at gradually increasing concentrations of epon. Samples were embedded in PELCO 21-cavity molds (Plano) and cured at 60 °C.

#### Ultrathin sectioning and transmission electron microscopy

Ultrathin sections (70 nm) were cut using an ultramicrotome (Leica UC6) with a 45° diamond knife (Diatome). Sections were stained with uranyl acetate and lead citrate and imaged using a JEM-2100 Plus TEM (JEOL) operating at 80 kV, equipped with a OneView 4 K camera (Gatan).

#### Serial sectioning and 3D reconstruction

Ribbons of five consecutive ultrathin sections were collected on Pioloform-coated slot grids (Science Services). A total of 60 sections were imaged for 3D reconstruction. Images were aligned using FIJI (TrakEM2 plugin), and nucleus, mitochondria and lipid droplets were manually segmented in Microscope Imaging Browser (MIB, v2.82). The final model was processed in IMARIS (v9.9.1) for 3D reconstruction.

### Quantification of mitochondrial density

Transmission electron micrographs (TEMs) at ×2,500 magnification were used to quantify mitochondria in wild-type and knockout conditions. Forty-eight images per condition were captured from random sample positions. Mitochondrial quantification was performed using unbiased counting frames in ImageJ (Fiji). For each image, a 3×3 grid of counting frames covering 25% of the image was randomly placed. Mitochondria fully enclosed or intersecting permissive lines were counted; those intersecting restrictive lines were excluded. The multi-point tool in Fiji was used for tracking. The numerical density (*N*) of mitochondria was calculated as: *N* = (number of mitochondria / number of counting frames) × area per frame (µm²).

### Quantification of lipid droplet density and area

Low-magnification TEM micrographs (×80–150 magnification) were acquired, including 20 images from knockout and 12 from wild-type samples. The largest possible rectangular sample regions were extracted using Fiji, excluding grid and void areas. For 12 images per condition, 512×512 pixel regions were cropped and segmented using the Cellpose cyto3 model. Segmentation results were saved as ImageJ ROI files, manually corrected in QuPath and re-applied to all extracted regions using a refined Cellpose model. Lipid-droplet number and size were measured using a FIJI macro. A guard zone (15% of image diameter) was applied to exclude lipid droplets cut off by image borders. To avoid biased exclusion, the lower and left boundaries were restrictive (excluding intersecting droplets), while the upper and right boundaries were permissive (including intersecting droplets).

### Primary brown adipose tissue mesenchymal stem cells cultures and their differentiation to mature brown adipocytes

#### Isolation of brown adipose tissue mesenchymal stem cells cultures

A six-well plate was prepared with BAT collagenase digestion buffer (123 mM NaCl, 5 mM KCl, 1.3 mM CaCl₂, 5 mM glucose and 100 mM HEPES, pH 7.4) on ice. Sterile equipment (Falcon tubes, reaction tubes, syringes, cannulas and a dish) was disinfected with 70% ethanol. The two brown fat pads were dissected from newborn mice, placed in collagenase digestion buffer, minced with scissors and transferred to a Falcon tube on ice. A tail tip was collected in a reaction tube for genotyping. For collagenase digestion, Falcon tubes were incubated in a water bath with gentle shaking until complete tissue dissociation. The suspension was drawn into syringes using 0.9 × 70 mm (20 G × 2) cannulas, then filtered through a 100-μm nylon mesh into a fresh Falcon tube. The middle phase was carefully collected, avoiding the upper (mature adipocytes) and lower (tissue remnants) phases. The suspension was then filtered through a 30-μm nylon mesh and centrifuged. The cell pellet was resuspended in brown adipose tissue mesenchymal stem cell culture medium and transferred to a six-well plate for further analyses.

#### Immortalization of brown adipose tissue mesenchymal stem cells

Primary brown adipose tissue mesenchymal stem cells (passage 0) were immortalized 24 h post-isolation through lentiviral transduction with a virus expressing SV40 large T-Antigen (L-TAg) under the PGK promoter. Immortalized brown preADs (mBA) were expanded in mBA growth medium at 37 °C, 5% CO₂. The virus solution was thawed and centrifuged at 16,000*g*, and 200 ng viral reverse transcriptase (per well of a 6-well plate) was mixed with 800 μl brown adipocyte (BA) growth medium (high-glucose DMEM without pyruvate). After removing the culture medium, cells were exposed to viral medium, followed by PBS washes and replacement with fresh BA growth medium.

#### Cultivation and storage of brown adipose cells

Cells were maintained in growth medium at 37 °C, 5% CO₂, ensuring cultures did not reach full confluence. For passaging, cells were detached through trypsinization, resuspended in growth medium, and centrifuged at 200*g*. Pellets were resuspended in growth medium and mixed 1:1 with freezing medium to achieve a final DMSO concentration of 10% at a cell density of 1 million cells ml^−1^. Cell suspensions were transferred to cryogenic vials (1 ml per vial) and stored at −80 °C, followed by transfer to liquid nitrogen (−196 °C) for long-term storage.

#### Differentiation of mature brown adipocytes

In each well of a six-well plate, 1.8 × 10⁵ cells were seeded in growth medium (day –4). Once confluent, the medium was replaced with differentiation medium (growth medium + 0.5 µg ml^−1^ insulin + 1 nM triiodothyronine) (day –2). Adipogenic differentiation was induced by switching to freshly prepared induction medium (growth medium + 5 µM dexamethasone + 250 µM IBMX (3-Isobutyl-1-methylxanthine) + 0.5 µg ml^−1^ insulin + 1 nM triiodothyronine (day 0). After induction, cells were maintained in BA differentiation medium, which was replenished every second day. By day 8, cells were fully differentiated into mature brown adipocytes.

### Cell culture treatments

Treatments were initiated from day 3 upon switching to differentiation medium: actinonin (150 μM, DMSO; applied for 96 h, with replacement every second day); NCT-503 (20 μM, DMSO; applied for 72 h, with replacement every 24 h); octyl d-2HG (500 μM, DMSO; applied for 72 h, with replacement every 24 h); and ISRIB (1 μM, DMSO; applied for 72 h, with daily renewal).

### Lipid content analysis

#### BODIPY staining and FACS

Cells were washed with PBS and stained with 1× BODIPY solution in PBS in the dark. After staining, cells were trypsinized, and trypsinization was stopped with FACS buffer (PBS + 2% BSA). The cell suspension was filtered through a 60-μm strainer and transferred to fresh Eppendorf tubes for analysis. The gating strategy used for flow cytometry analysis is provided in Supplementary Figure 1a.

#### Oil red O staining

Cells were washed with PBS and fixed in 10% formalin for 30 min at room temperature. After fixation, cells were washed twice with water, followed by a 5-min wash with 60% isopropanol at room temperature. The cells were then completely dried. A working solution of Oil red O (ORO) was added to the wells and incubated for 10 min at room temperature. After incubation, cells were washed four times with water, and images were acquired for further analysis. The ORO stock solution was prepared as 0.35% ORO in isopropanol, and the working solution was prepared with 60% of ORO stock in water.

### Immunofluorescence staining

Cells were seeded and differentiated on coverslips before direct fixation with 4% paraformaldehyde in PBS (pH 7.4) at room temperature, followed by washes with PBS. Blocking was performed with 5% BSA and 0.3% Triton X-100 in PBS. Coverslips were incubated with primary antibodies diluted in 1% BSA and 0.3% Triton X-100 in PBS at 4 °C, followed by PBS washes. Secondary antibodies were applied in 0.3% Triton X-100 in PBS in the dark, followed by final PBS washes. Coverslips were dipped in water, mounted on elvanol and imaged using an SP8 confocal microscope (Inverse, DMi 8 CS, Leica Microsystems).

### Ex vivo adipocyte lipolysis assay

To assess lipolytic activity in BAT from CLPP-KO and control mice, mice were fasted for 6 h, and BAT pads were excised under sterile conditions. Tissues were digested with collagenase type 2 (Worthington Biomedical) in Krebs-Ringer Solution (KRH) + 1% BSA. For each condition, BAT adipocytes from 4–5 mice were pooled, and 150,000–200,000 isolated adipocytes were incubated in KRH buffer + 4% BSA with either 15 nM isoproterenol (ISO) or 10 μM forskolin. Lipolysis was assessed by measuring free glycerol release in the supernatant using Free Glycerol Reagent (Sigma-Aldrich), with absorbance recorded on a Synergy H1 plate reader (BioTEK). Glycerol content was normalized to total protein content, determined by the Pierce Bradford Assay Kit (Thermo Scientific).

### Ex vivo oxygen consumption rate in BAT lysates

Oxygen consumption in BAT tissue lysates was measured using an OROBOROS Oxygraph-2K electrode at 37 °C with magnetic stirring. Samples were placed in 2 ml incubation medium (containing EGTA, MgCl₂, K-lactobionate, taurine, KH₂PO₄, HEPES, sucrose and BSA; pH 7.1). In vitro respiration levels were recorded at steady state, followed by sequential addition of the following substrates: endogenous (state 1), ADP (state 2), succinate (state 3), oligomycin (state 4) and FCCP (uncoupled respiration). Respiration rates were normalized to total protein content.

### Respiration analysis using Seahorse XFe-96 Analyzer

Mitochondrial OCR and ECAR were measured using the Seahorse Bioscience XFe-96 Analyzer (Agilent Technologies) following the standard mitochondrial stress test protocol. Differentiated mBA cells (day 8) were assayed. Cells were washed with unbuffered assay medium (DMEM without phenol red, supplemented with glucose, sodium pyruvate and glutamine) and incubated in a CO₂-free incubator at 37 °C. The sensor cartridge was hydrated and injection ports were loaded with oligomycin (1 μM), FCCP (1.5 μM) and rotenone-antimycin A (1 μM each) before calibration. The assay was then performed, and post-measurement normalization was conducted using BioTek Cytation 5 (Agilent Technologies) with Hoechst 33342 staining for cell counting. Data analysis was performed using Seahorse XFe Wave Software (Agilent). Basal respiration, leak respiration, maximal respiration and acidification were calculated on the basis of OCR and ECAR values before and after specific injections, with non-mitochondrial oxygen consumption corrected using rotenone and antimycin A.

### Atomic force microscopy measurements on cultured cell nuclei

AFM measurements were performed on cell monolayers plated on silicon elastomers using the JPK NanoWizard 4 XP (Bruker Nano) atomic force microscope mounted on Zeiss AxioObserver inverted fluorescent microscope and operated through JPK SPM Control Software v.5. Triangular non-conductive silicon nitride cantilevers (MLCT, Bruker Daltonics) with a nominal spring constant of 0.01 N m^−1^ were used for the nanoindentation experiments of the apical surface of cells and the nucleus. For all indentation experiments, forces of up to 3 nN were applied, and the velocities of the cantilever approach and retraction were kept constant at 2 μm s^−1^, ensuring an indentation depth of 500 nm. All analyses were performed with JPK Data Processing Software (Bruker Nano). Before fitting the Hertz model corrected by the tip geometry to obtain Young’s modulus (Poisson’s ratio of 0.5), the offset was removed from the baseline, the contact point was identified, and cantilever bending was subtracted from all force curves.

### Lentivirus production and stable cell line generation

To overexpress d-2HGDH in wild-type and CLPP-KO mBA cells, a lentiviral transfer plasmid encoding d-2HGDH–hemagglutinin (HA) (VectorBuilder) was used. Lentivirus was produced in HEK293FT cells via cotransfection of the d-2HGDH–HA plasmid with psPAX2 (Addgene plasmid no. 12260) and pMD2.G (Addgene plasmid no. 12259) using Lipofectamine 2000 (Invitrogen). Viral supernatants were collected, pooled, clarified by centrifugation and concentrated with Lenti-X Concentrator (Takara Bio). The viral pellet was resuspended in PBS, aliquoted and stored at −80 °C.

#### Transduction of mBA cells

Wild-type and CLPP-KO mBA cells were seeded in 6-well plates and transduced with 10 μl concentrated lentivirus in 6 μg ml^−1^ polybrene. After 24 h, the medium was replaced, and cells were expanded before puromycin selection (2 μg ml^−1^, 48 h).

### PHGDH activity assay

The PHGDH activity assay was performed using the ab273328 kit (Abcam), following the manufacturer’s instructions. In brief, cells in a 6-well plate were washed with PBS, homogenized on-plate in PHGDH Assay Buffer and centrifuged at 10,000*g*, 4 °C. The supernatant was collected, and total protein concentration was determined using a BCA assay. Then, 30 μg of protein was loaded into a 96-well plate, with the final volume adjusted to 50 μl using PHGDH assay buffer. A NADH standard curve was generated using dilutions of 1.25 mM NADH standard, and a PHGDH positive control was prepared. A reaction mix of assay buffer and developer was added to all wells. Absorbance at 450 nm was measured in kinetic mode at 37 °C using an EnSpire plate reader.

### RNA isolation from mouse tissues and primary adipocytes

Total RNA was isolated from mouse BAT and primary adipocytes using TRIzol (Life Technologies) or TRI reagent (Sigma), followed by purification with RPE buffer (Qiagen). Tissues were homogenized in 1 ml TRIzol using ceramic beads (Mobio, Dianova) in a FastPrep homogenizer (MP Biomedicals) at 5,500 r.p.m. After incubation at room temperature, 200 μl chloroform was added, mixed and centrifuged at 12,000*g*, 4 °C. The aqueous RNA-containing phase was collected and precipitated with 500 μl isopropanol, followed by washing with 75% ethanol and resuspension in ultrapure H_2_O. RNA was incubated at 60 °C before a concentration and purity assessment using a NanoDrop ND-1000 UV-Vis spectrophotometer (Peqlab). For DNase treatment, 10 μg RNA was digested with DNase I (New England Biolabs) in 10× digestion buffer. DNase was inactivated at 75 °C, and the RNA concentration was reassessed before storage at −80 °C. The RNA quality was validated using the Agilent RNA 6000 Nano Kit on an Agilent 2100 Bioanalyzer (Agilent Technologies).

### Quantitative PCR

Isolated RNA was treated with DNase I (New England Biolabs) to digest the residual DNA, following the manufacturer’s protocol. The concentration of the samples was adjusted to 200 ng µl^−1^. Next, complementary DNA (cDNA) was synthesized from the digested samples using the High-Capacity Reverse Transcription kit (Applied Biosystems). Quantitative PCR (qPCR) reactions were performed in 384-well plates using the Brilliant III Ultra-Fast SYBR Green qPCR Master Mix (Agilent Technologies), according to the manufacturer’s instructions; 50 ng of cDNA was added to each reaction. The standard programme was run at the Applied Biosystems QuantStudio 7 Flex Real-Time PCR System (Fisher Scientific). The qPCR data were collected using the QuantStudio 12 K Flex Software v1.6 (Applied Biosystems). Relative expression of the target gene was calculated by normalization to endogenous control gene (*Hprt*) and was quantified using the 2^−ΔΔCt^ method.

### Isolation of cytoplasmic and peri-lipid-droplet mitochondria from BAT

BAT was collected, rinsed in PBS, weighed and minced with fine scissors. The minced tissue was suspended in SHE-BSA buffer (50 mM sucrose, 5 mM HEPES, 2 mM EGTA, pH 7.2, 2% FFA BSA) at 1 ml per 100 mg tissue. Homogenization was performed using a glass-Teflon electric homogenizer (7 strokes) followed by a glass-glass Dounce homogenizer (13 strokes). The homogenate was transferred to a 50-ml Falcon tube, and residual tissue was washed out with SHE-BSA buffer. Centrifugation at 900*g* (4 °C) separated the fractions. The supernatant, containing cytoplasmic mitochondria, was carefully collected using a syringe, avoiding disruption of the floating fat layer or debris pellet. The fat layer, containing lipid-associated PDMs, was scraped into a new tube, resuspended in SHE-BSA buffer, and vigorously shaken to emulsify the fat and release PDMs. Both fractions (cytoplasmic mitochondria and PDMs) were centrifuged at 9,000*g* (4 °C), and pellets were washed in SHE-BSA buffer, transferred to 1.5-ml Eppendorf tubes and centrifuged again. The final pellets were resuspended in SHE buffer without BSA for immediate analysis or snap-frozen in liquid nitrogen for long-term storage. BAT was collected and rinsed twice in PBS.

### Protein isolation

#### Tissue lysis

BAT pads were homogenized in 700–800 μl cold organ lysis buffer (50 mM HEPES pH 7.4, 50 mM NaCl, 1% Triton X-100, 100 mM NaF, 10 mM EDTA, 0.1% SDS, 10 mM Na-orthovanadate, 2 mM PMSF, 1× protease inhibitor cocktail (Sigma-Aldrich), 1× PhosphoSTOP phosphatase inhibitor cocktail (Roche)) using 1.4 mm ceramic beads (Omni International) in a Precellys tissue homogenizer (Bertin Technologies) at 5,500 r.p.m. (2 × 20 s, 30 s pause). Lysates were cleared by centrifugation (20,000*g*, 4 °C, 45 min) and transferred to fresh tubes.

#### Cell lysis

Cells were washed with PBS and lysed on-plate using RIPA buffer (50 mM Tris-HCl pH 7.4, 1% Triton X-100, 0.5% sodium deoxycholate, 0.1% SDS, 150 mM NaCl, 2 mM EDTA, 50 mM NaF, 1× protease inhibitor cocktail). Lysates were incubated on ice for 30 min with occasional vortexing, followed by 10 cycles of sonication (30-s intervals, 4 °C) using the Bioruptor Pico (Diagenode). Cell debris was pelleted (14,000*g*, 4 °C, 15 min), and the supernatant was collected.

In both cases, the protein concentration was measured using the Bradford assay (Sigma-Aldrich), following the manufacturer’s instructions. Lysates were stored at −80 °C.

### SDS–polyacrylamide gel electrophoresis and western blot

Proteins were separated by SDS–polyacrylamide gel electrophoresis (SDS–PAGE) using the Mini-PROTEAN Tetra Cell (BioRad). Fifty micrograms of protein lysate was mixed with Laemmli buffer (50 mM Tris-HCl, 2% SDS, 10% glycerol, 1% 2-mercaptoethanol, 12.5 mM EDTA, 0.02% bromophenol blue, pH 6.8) and incubated at 95 °C for 5 min. Samples were loaded onto an 8–12% polyacrylamide gel alongside a PageRuler Prestained Protein Ladder (Thermo Fisher Scientific). Electrophoresis was performed at 90 V until the loading dye reached the separating gel, followed by 150 V until completion in running buffer (25 mM Tris-HCl, 250 mM glycine, 0.1% SDS, pH 8.3).

Proteins were transferred to a nitrocellulose membrane using the Criterion Blotter (BioRad) through wet transfer at 400 mA in transfer buffer (30 mM Tris-HCl, 240 mM glycine, 0.037% SDS, 20% methanol) at 4 °C. Transfer efficiency was assessed with Ponceau-S staining (Sigma-Aldrich). Membranes were blocked in 5% milk-PBST, 3% BSA-TBST or 2% fish skin gelatin-TBST, depending on antibody requirements.

Membranes were incubated overnight at 4 °C with primary antibodies, followed by three 5-min washes in PBST or TBST. Secondary antibody incubation was performed at room temperature, followed by additional washes. Detection was performed using ECL solution (GE Healthcare), and membranes were exposed to Super RX films (Fujifilm), developed using an automatic film processor (Kodak) and scanned with a V800 Transparency Scanner (Epson) at 600 dpi, 16-bit grayscale.

### Blue native polyacrylamide electrophoresis

Blue native (BN)–PAGE was performed using the NativePAGE Novex Bis-Tris Gel System (Life Technologies), according to the manufacturer’s instructions. Ten micrograms of mitochondria were lysed in 4% digitonin, and lysates were cleared by centrifugation (20,000*g*, 4 °C). Supernatants were mixed with loading dye (50% glycerol, 5% Coomassie) and loaded onto 4–16% native acrylamide gels (Life Technologies). Electrophoresis was conducted at 4 °C, 150 mV. Antibodies used for BN–PAGE were as follows: ATP5A1 (Abcam ab14748) 1:1,000; NDUFA9 (Molecular Probes 459100) 1:1,000; NDUFV1 (Proteintech 11238-1-AP) 1:1,000; SDHA (Molecular Probes 459200) 1:10,000; UQCRC1 (Molecular Probe 459140) 1:1,000.

### In-gel activity assay for complex I and V

After BN–PAGE, the gel was washed and incubated in NADH-nitrotetrazolium blue solution (0.1 mg ml^−1^ NADH, 2.5 mg ml^−1^ nitrotetrazolium blue, 5 mM Tris-HCl, pH 7.4) at 37 °C for complex I activity. Gels were then destained in 50% methanol, 10% acetic acid and rehydrated in double-distilled H₂O. For complex V activity, the gel was incubated in assay buffer (35 mM Tris-HCl, 270 mM glycine, 14 mM magnesium sulfate, 0.075% lead nitrate, 0.8 mM ATP, pH 7.8, 20% methanol). ATP hydrolysis was visualized as a white band of precipitated lead phosphate. The reaction was stopped with 50% methanol, and the gel was rinsed with deionized water. The gel was documented using a V800 Transparency Scanner (Epson).

### Proteomics: label-free quantification of tissue proteomes

The entire piece of frozen adipose tissue was ground to powder in liquid nitrogen and after the liquid nitrogen evaporated, the powder was resuspended in 100 μl of the urea lysis buffer (8 M urea; 50 mM triethylammonium bicarbonate (TEAB); 1× protease inhibitor). The sample solution was subjected to sonication with the Bioruptor Pico sonication machine (Diagenode) with a cycle of 30 s on and 30 s off for 10 min. Afterwards, the sample was centrifuged at 20,000*g* for 15 min at 4 °C to pellet the debris, which was discarded after centrifugation. The protein concentration of the samples was determined using the Bradford reagent (Sigma-Aldrich), according to the manual, and 55 μg of each sample was transferred to a new tube. The sample was treated with dithiothreitol (DTT, final concentration 5 mM) and incubated at 25 °C for 1 h. Then, chloroacetamide was added final concentration 40 mM) and incubated for 30 min in the dark. This was followed by treatment with lysyl endopeptidase (Lys-C), at a protease to sample protein ratio of 1/75 and then incubated for 4 h at 25 °C. The sample was next diluted with 50 mM TEAB buffer to reach a final concentration of ≤2 M urea. Then, trypsin was added at a 1:75 ratio, and trypsinisation was performed overnight at 25 °C with mild agitation. The following day, the sample was acidified with formic acid (final concentration 1%) to stop the digestion. Next, lysates were loaded on SDB RP StageTips supplied by the CECAD proteomics core facility and submitted to the facility for mass spectrometry (MS) analysis, following the standard procedures of the facility. The raw data were analysed with the MaxQuant proteomics software. The samples were analysed by CECAD proteomics facility.

### mRNA sequencing and differential expression analysis

mRNA sequencing was performed in-house using 1 μg total RNA for library preparation with the NEBNext RNA Prep Kit, followed by paired-end (2×50 bp) sequencing on an Illumina NovaSeq 6000. Sequencing reads were aligned to the mouse reference genome (Gencode vM25) using STAR aligner (v2.7.9a). Gene features were counted using featureCounts (v2.0.3), and genes with low expression levels were filtered out using edgeR (v4.0.16) with the filterByExpr function. For the Embryo Inject dataset, a more rigorous filtering approach was applied (with a minimum count of 40). Differential expression analysis was conducted using edgeR’s voomLmFit function with sample quality weights (sample.weights = TRUE) in a linear model (0 + group), where ‘group’ represented the experimental conditions. Genes with an adjusted *P* < 0.05 were considered differentially expressed.

### Bulk deconvolution

To assess whether genotype-specific differences in bulk gene expression were influenced by cell-type composition, the BisqueRNA R package was used to estimate cell-type proportions from bulk RNA-seq read counts. The Shamsi et al. dataset was selected as a single-cell reference owing to its similar tissue origin^34^. Because only marker genes for UMAP clusters were available, the original UMAP projection was reconstructed and clustered using the Seurat FindClusters function (resolution, 1.4). However, later analyses suggested that a resolution of 0.4 might have been a better choice. For consistency with the original procedure, Seurat v3.0 and R v3.6.2 were used.

#### Cell-type assignment

Cell-type scoring was performed using UCell (v2.0.1), with the ten strongest positive and negative marker genes per cluster. Because UCell scores alone do not always provide unique assignments, two approaches were tested. The first was clustering the ucell score matrix: the ComplexHeatmap package (v2.12.1) was used to cluster cells by their UCell scores, revealing groups of related cell types. However, owing to memory constraints, clustering was performed on 200 random sub-matrices, and thresholds for defining clusters remained arbitrary. The second was identifying high-scoring cell types in UMAP clusters: a one-sided *t*-test compared each cell type’s UCell score distribution within a cluster to its global distribution. If only one cell type showed significant enrichment, the cluster was uniquely assigned. If multiple cell types were significant, a coarse classification was applied. For bulk deconvolution, only samples collected at room temperature were used, restricted to genes present in both datasets (18,953 genes, 22,219 cells)^34^. Cell-type assignments were based on resolution-0.4 clustering, with a stringent inclusion threshold (*P* ≤ 2.225074 × 10^−308^). This resulted in 15 cell types, of which B_cells and VSM_1_cells were not composite. After removing 738 unassigned cells, 21,481 cells remained. BisqueRNA was applied to the salmon.merged.gene_counts_length_scaled.tsv bulk dataset, using only BAT samples, analyzing one genotype at a time, and restricting genes to those also found in the single-cell dataset.

#### Genotype-specific cell-type proportions

To determine whether BAT genotypes differed in cell-type composition, the predicted coarse cell-type proportions were examined. However, high variability in genotype replicates made it difficult to draw statistical conclusions. Additionally, proportion data are compositional, meaning changes in one cell type’s frequency inherently affect the others. Instead of statistical analysis, a visual representation was developed: Seurat UMAP coordinates were extracted. Ggplot2 was used to colour cells by their assigned coarse cell type, with predicted proportions coded by transparency in the bulk dataset. This approach revealed that resolution-0.4 clustering corresponded more clearly to high-level UMAP features than did resolution-1.4 clustering, suggesting that 0.4 might be the better choice of resolution.

### ChIP–seq of H3K4me3 in mature brown adipocyte cells

ChIP–seq libraries were prepared from four biological replicates per condition (WT, KO, WT + 2HG). First, 1 × 10⁷ cells per condition were cross-linked with 1% formaldehyde, quenched with glycine (0.125 M), washed in PBS and pelleted by centrifugation. Cells were lysed in lysis buffer (50 mM HEPES pH 7.9, 140 mM NaCl, 1 mM EDTA, 10% glycerol, 0.5% NP-40, 0.25% TritonX-100) with protease inhibitors and homogenized in a pre-chilled dounce homogenizer. Nuclei were pelleted, washed with wash buffer (10 mM Tris pH 8.0, 200 mM NaCl, 1 mM EDTA, 0.5 mM EGTA) and resuspended in shearing buffer (0.1% SDS, 1 mM EDTA, 10 mM Tris pH 8.0). Chromatin was sonicated using a Bioruptor Pico (Diagenode) for 70 cycles. For chromatin immunoprecipitation (ChIP), 30 μg of DNA was incubated with 5 μl H3K4me3 antibody (Active Motif, 39159) in 1% Triton X-100 and 150 mM NaCl. Protein G Dynabeads (Invitrogen) were prewashed with IP buffer, added to the immunoprecipitated chromatin and washed sequentially with TSE-150, TSE-500, LiCl and TE buffers. DNA was eluted in PK digestion buffer (20 mM Hepes pH 7.5, 1 mM EDTA, 0.5% SDS) with RNAse A (Thermo Fisher Scientific) and proteinase K, followed by reverse cross-linking with NaCl (0.3 M). DNA purification was performed using the Nucleospin Gel and PCR Clean-up kit (Macherey-Nagel). For library preparation, up to 100 ng ChIP DNA underwent end repair, A-tailing and adapter ligation, followed by PCR amplification (12–15 cycles). Libraries were validated (TapeStation, Agilent Technologies), quantified (Qubit, Invitrogen), pooled and sequenced on an Illumina NovaSeq 6000 (paired-end 2×100 bp protocol).

### Analysis of ChIP–seq data

Raw FASTQ files were processed using the SnakePipes pipeline^63^. In brief, fastq files were trimmed with fastp using the following parameters: -–trim_poly_g–-trim_poly_x -Q -L–-correction. Trimmed reads were mapped to the GRCm39 mouse reference genome with BWA–MEM, and peaks were called using Genrich, which integrates replicate data by computing *P* values per replicate and combining them using Fisher’s method to generate *q* values for peak calling. Coverage plots and heatmaps generated using deeptools. Peak count frequency at the TSS sites and feature distribution of all identified peaks performed using ChIPseeker. Kyoto Encyclopedia of Genes and Genomes and GO analyses were conducted as described previously, focusing on differentially enriched H3K4me3 peaks within ±2 kbp of promoter regions. Data visualization was conducted using the Integrative Genomics Viewer (https://igv.org) and custom plots.

GSEA analysis was performed using GSEA v4.3.2 (Windows application, build 13) with default settings^64^. Data visualization was conducted using the Integrative Genomics Viewer (web-based version)^65^ and custom plots.

### Single-cell immunophenotyping from tissue

For single-cell suspensions, bone marrow was extracted from the femur of adult mice by flushing with 10 ml of FACS buffer using a 27-G needle and syringe, then passed through a 40-µm strainer. BAT was dissected and immediately transferred to ice-cold digestion solution. Tissues were cut in small pieces with scissors and incubated in digestion solution containing collagenase IV (1 mg ml^−1^), and DNase I (0.01 mg ml^−1^) in RPMI for 40 min at 37 °C in the shaker. Digestion was stopped by adding 10% FBS on ice. Tissue homogenates where smashed and washed with FACS buffer against a 70-µm strainer using the back of a syringe. Pellets were resuspended in 1 ml red blood cell lysis buffer (Roche) and incubated for 10 min at room temperature for lysis of erythrocytes.

Subsequently, 10 ml FACS buffer (5% FCS in PBS) was added and cells were centrifuged at 300*g* for 5 min at 4 °C. The cells were pre-incubated with fix viability dye in PBS (1:1,000) for 10 min at room temperature; for BAT, a mix of anti-mouse-FcγIII/II-receptor (CD16/CD32) blocking antibodies (1:500) was added. After being washed with FACS buffer, cells were stained with the fluorochrome-conjugated antibodies (1:100 0.25–1 μg; listed below). Only BAT cells were fixed and permeabilized using the FoxP3 kit, following the manufacturer’s instructions, and were then incubated with antibodies to iNOS and Arg-1 for 20 min at room temperature for intracellular staining. After washing and filtering, cells were resuspended in 500 µl (BM) or 250 µl (BAT) FACS buffer, and 25 µl of counting beads were added to the samples (at a concentration of 1,000 beads per µl) to obtain absolute numbers. For absolute quantification, the following equation was used: absolute count (cells µl^−1^) = (cell count × counting beads volume) / (counting bead count × sample volume) × counting bead concentration.

Each population was expressed in absolute numbers (cells µl^−1^), and BAT results were further normalized to the initial tissue weight (g) for comparison across samples. Cells were acquired on a FACS Symphony A3 flow cytometer (BD) using Diva software (BD) and further analysed using FlowJo analytical software (FlowJo version 10.0.8). Owing to inadequate result quality, data collected for iNOS and Arg-1 were excluded from further analysis. Compensation beads were use to generate compensation panel and calculated by diva software before sample acquisition. Background fluorescence levels of iNOS, Arg-1, CD80 and CD80 were determined using Fluorescence Minus One (FMO), although data from these populations were excluded owing to low cell counts. The buffers used were as follows: FACS buffer (5 mg BSA + 5 ml EDTA 0.4 M + 500 ml of PBS); digestion buffer (250 µl DNase I (Merck, 200,000 U µl^−1^), 0.2 mg ml^−1^ Liberase (Roche) and 1 mg ml^−1^ collagenase IV in RPMI medium). The details of the antibodies used can be found in the reporting summary. The gating strategy used can be visualized in Supplementary Figure 1b,c.

The antibodies used are provided in the table.

**Table:** 

Antibody	Conjugates	Laser name in BD	Clone
CD45	FITC	BB515	30-F11
B220	Allophycocyanin (APC), Cyanine7	APC-H7	RA3-6B2
CD115	Brilliant Violet 605	VV605	AFS98
CD117	Brilliant Violet 510	BV480/510	ack2
CD11b	BUV661	BUV661	M1/70
CD11c	BUV395	BUV395	HL3
CD127 (IL-7Rα)	Peridinin-chlorophyll protein (PerCP), Cyanine5.5	BB700	A7R34
CD135	Phycoerythrin (PE)	BY6584	A2F10
CD16/32	Brilliant Violet 421	BV421	93
CD172a (SIRPα)	PE, Dazzle 594	PE-CF594	P84
CD19	APC, Cyanine7	APC-H7	6D5
CD34	PE, Cyanine7	BYG790	MEC14.7
CD3E	APC, Cyanine7	APC-H7	145-2C11
CD45	Alexa Fluor 700	APC-R700	30-F11
CD49b	APC, Cyanine7	APC-H7	DX5
F4, F80	BUV395	BUV563	T45-2342
Ly-6A, Ly-6E (Sca-1)	PE,Dazzle 594	BB515	D7
MHCII	PerCP, Cyanine5.5	BB700	M5-114.15.2
NK1.1	APC, Cyanine7	APC-H7	PK136
Ter119	APC, Cyanine7	APC-H7	TER119
Ly-6A, Ly-6E (Sca-1)	PE, Dazzle 594	PE-CF594	D7
TruStain FcX (anti-mouse-CD16/CD32)	None	None	93

### Metabolite extraction from tissues

Metabolite extraction solution (50% methanol, 30% acetonitrile (ACN), 20% water, 5 µM valine-d8 as an internal standard) was added to 10–20-mg frozen BAT or iWAT tissue samples at an extraction ratio of 25 µl mg^−1^ on dry ice. Samples were then homogenized using a Precellys 24 tissue homogenizer (Bertin Technologies). The resulting sample suspension was vortexed, mixed at 4 °C in a Thermomixer for 15 min at 1,500 r.p.m. and then centrifuged at 16,000*g* for 20 min at 4 °C. The supernatant was collected for liquid chromatography–MS (LC–MS) analysis.

### Metabolite extraction from cells

The cells were counted at seeding to estimate the amount of extraction solution to use. Using 500 μl extraction buffer per 106 cells is the recommended starting point, although the ratio might need to be optimized for certain cell types in a separate pilot experiment. The medium was removed from wells, and cells were washed quickly (less than 10 s) with PBS twice at room temperature. Extra PBS was carefully removed by inverting the plate over a piece of tissue paper. After the last wash, each well was aspirated quickly to remove all residual PBS. The plate was placed on dry ice, and 500 μl of extraction buffer was added per 1,000,000 cells. The plate was gently swirled such that cells were covered by the extraction buffer. The plate was incubated for 20 min on a dry ice–methanol bath to break cell membranes. The cells were scraped off the plate and the entire suspension was transferred into prechilled Eppendorf tubes. The cell-extract suspension was shaken for 15 min at 4 °C in a Thermomixer at maximum speed (Thermomixer set at 4 °C and placed in a cold room for this step). The tubes were then centrifuged for 20 min at 4 °C at maximum speed (13,000 r.p.m. or higher). Only the top 80% of the supernatant was collected and put into prelabelled autosampler vials, taking care not to disturb the solid debris. A pooled sample was prepared by taking 10 μl of each sample from the same matrix. Samples were stored at –80 °C until further analysis.

### Metabolite extraction from cell culture medium

Eighty microliters of cell culture medium was taken and centrifuged for 5 min (4 °C) at maximum speed to eliminate dead cells. Fifty microliters of the supernatant was taken and added to a prelabelled Eppendorf tube containing 350 μl of extraction solution on dry ice. The samples were mixed for 15 min in a Thermomixer at 4 °C at maximum speed, followed by centrifugation for 20 min at 4 °C at maximum speed (13,000 r.p.m. or higher). The top 80% of the supernatant was carefully transferred into an autosampler vial, taking care not to disturb the insoluble debris. A pooled sample was prepared by mixing equal volumes of each sample extract in a single vial. Samples were stored at –80 °C until further analysis.

### Liquid chromatography coupled to mass spectrometry

Hydrophilic interaction chromatographic (HILIC) separation of the targeted metabolite (2HG) was achieved using a Millipore Sequant ZIC-pHILIC analytical column (5 μm, 2.1×150 mm) equipped with a 2.1×20-mm guard column (both 5-mm particle size) with a binary solvent system. Solvent A was 20 mM ammonium carbonate, 0.05% ammonium hydroxide; Solvent B was ACN. The column oven and autosampler tray were held at 40 °C and 4 °C, respectively. The chromatographic gradient was run at a flow rate of 0.200 ml min^−1^ as follows: 0–2 min, 80% B; 2–17 min, linear gradient from 80% B to 20% B; 17–17.1 min, linear gradient from 20% B to 80% B; 17.1–23 min, hold at 80% B. Samples were randomized, and the injection volume was 5 μl. A pooled quality control sample was generated from an equal mixture of all individual samples and analysed interspersed at regular intervals. Metabolites were measured with Vanquish Horizon UHPLC coupled to an Orbitrap Exploris 240 mass spectrometer (both Thermo Fisher Scientific) using a heated electrospray ionization source. The spray voltages were set to –2.8 kV, the RF lens value was set at 70, the heated capillary was held at 320 °C and the auxiliary gas heater was held at 280 °C. The flow rate for sheath gas, aux gas and sweep gas were set to 40, 15 and 0, respectively. For MS1 scans, the mass range was set to *m/z* = 100–600, AGC target set to standard and maximum injection time set to auto. Data acquisition for experimental samples used full scan and selected ion monitoring (SIM) modes (targeting 2HG *m/z* = 147.0299) at an Orbitrap resolution of 120,000 in negative mode.

### Targeted metabolomics for 2HG quantification

The identity of metabolite 2HG was verified based on two criteria: (1) the precursor ion *m/z* corresponded to within 3 p.p.m. of the expected mass derived from its chemical structure, and (2) the retention duration was within a 5% range compared with that of a purified reference sample analysed using the identical chromatography technique. The examination of chromatograms and integration of peak areas was carried out using the Tracefinder software (v5.1, Thermo Fisher Scientific). The peak area of each identified metabolite was adjusted on the basis of the total ion count of the respective sample to compensate for any discrepancies arising from sample preparation or instrument assessment. These measurements were done in collaboration with the Frezza lab at the CECAD Research Center.

### Chiral derivatization of 2HG

TSPC derivatization was performed for LC–electrospray ionization–tandem MS (LC–ESI–MS/MS) analysis, under optimized conditions. Tissue supernatant was dried under nitrogen gas at 37 °C, resuspended in TSPC (2.5 mM in ACN) and pyridine and derivatized. The mixture was dried again, redissolved in 50% aqueous ACN containing phthalic acid, and 10 μl was injected into the LC–ESI–MS/MS system.

#### Liquid chromatography–electrospray ionization–tandem mass spectrometry analysis

Quantification of TSPC-labeled d-2HG and l-2HG was performed on an AB 3200 QTRAP mass spectrometer (Applied Biosystems) coupled to a Shimadzu LC-20AD HPLC. HPLC separation was conducted using an Inertsil ODS-3 column (250 × 2.0 mm, 5 μm) at 35 °C, with a formic acid–water (0.1%) and ACN–methanol (50:50) gradient at 0.2 ml min^−1^. Detection was performed with multiple reaction monitoring in negative-ion mode with optimized transitions for 2HG and phthalic acid. For comparison, the derivatization of d-2HG and l-2HG with diacetyl-l-tartaric anhydride (DATAN) was also performed, and the detailed procedure has been previously published^66^. Additional HR–MS experiments were conducted on a MicrOTOF-Q mass spectrometer (Bruker Daltonics) with an ESI source, using optimized parameters for maximal detection sensitivity.

### Cholesterol quantification in mature brown adipocytes through lLC–ESI–MS/MS

Cholesterol levels in mBAs were determined using LC–ESI–MS/MS. Adipose tissue was homogenized in Milli-Q water using a Precellys 24 Homogenizer, and protein content was measured using the Bradford assay. Lipids were extracted using the one-step extraction method^67^. For extraction, homogenized samples were mixed with chloroform, methanol, hydrochloric acid and cholesterol-d7 (internal standard). Lipid extracts were dried under nitrogen, resuspended in ammonium acetate in methanol, sonicated and centrifuged. Supernatants were analysed via LC–ESI–MS/MS using a Core-Shell Kinetex C18 column on a QTRAP 6500 mass spectrometer (AB SCIEX). UHPLC was performed isocratically with a 5 mM ammonium acetate–methanol mobile phase at 300 μl min^−1^. Cholesterol and cholesterol-d7 were detected in positive-ion mode using multiple reaction monitoring transitions. Endogenous cholesterol was quantified using an external calibration curve, normalized to protein content, and analysed with MultiQuant 3.0.3 software.

### Statistical analysis

Statistical significance was evaluated using tests appropriate for the distribution and design of each experiment. Depending on the context, paired two-tailed Student’s *t*-tests, one-way ANOVA or Kolmogorov–Smirnov tests were applied, as specified in the individual figure legends. Error bars indicate the s.d. unless otherwise noted. Statistical significance is reported as *P* values, denoted by asterisks (*P* < 0.05, *P < 0.01, **P < 0.001, ***P < 0.0001) or symbols (for example, ^##^*P* < 0.001), as defined in the figure legends. Unless stated otherwise, experiments were independently repeated at least three times to ensure reproducibility. Sample numbers indicated in figure legends refer to the number of replicates shown, not the total number of biological replicates in the study, with the exception of cell culture experiments. For all the cell culture experiments, *n* in the figure legends indicates the number of cells or nuclei analysed, from five independent wells for each experiment. No statistical methods were used to predetermine sample sizes, but sample sizes are consistent with those used in comparable published studies.

### Data collection and randomization

Mice or cell culture samples or dishes were randomly assigned to experimental groups. Data collection and outcome assessments were performed in a randomized manner, when applicable. Experimental conditions and sample processing were organized to minimize bias, with randomization applied during sample allocation and data acquisition. The data distribution was assumed to be normal but this was not formally tested. Researchers performing data collection and analysis were not blinded to experimental conditions.

### Reporting summary

Further information on research design is available in the Nature Portfolio Reporting Summary linked to this article.

## Supplementary information

Supplementary Information Supplementary Figure 1 and Supplementary Methods

Reporting Summary

Supplementary Tables 1–8

Supplementary Videos 1

Supplementary Videos 2

## Source data

Source Data Fig. 1 Uncropped blot scans, low mag TEM image and tissue images

Source Data Fig. 3 Uncropped tissue images

Source Data Fig. 5 Uncropped TEM image

Source Data Extended Data Fig./Table 1 Uncropped blots and gel scans

Source Data Extended Data Fig./Table 3 Uncropped blot scans

Source Data Extended Data Fig./Table 4 Uncropped blot scans

Source Data Extended Data Fig./Table 5 Uncropped blot scans

## Data Availability

Raw data for BAT tissue transcriptomics are publicly available from the Gene Expression Omnibus (GEO) under the accession number GSE271207; cell transcriptomics data are publicly available under the accession number GSE271358. All raw data related to proteomics experiments on BAT tissue and mBA cells are publicly available under the accession number PXD064288. All raw data for metabolomics from BAT tissue and cells are publicly available in the Zenodo repository (10.5281/zenodo.15357452)^68^. Raw data from the ChIP–seq experiment using mBA cells is publicly available under the accession number GSE296541. The following reference genomes were used in the manuscript: GRCm39 mouse reference genome and Gencode vM25. Source data are provided with this paper.

## Code availability

All analyses were performed using standard software packages as described in the Methods. The custom codes used in this study are available from the corresponding author upon request.
