# Supplementary material for: 2-hydroxyglutarate mediates whitening of brown adipocytes coupled to nuclear softening upon mitochondrial dysfunction
Source: Nat Metab. 2025 Aug 1;7(8):1593–613. doi: 10.1038/s42255-025-01332-8 (PMC12373511; doi:10.1038/s42255-025-01332-8)
Supplement: Supplementary file 1 — Supplementary Figure 1 and Supplementary Methods [file 42255_2025_1332_MOESM1_ESM.pdf]

nature metabolism

Supplementary information https://doi.org/10.1038/s42255-025-01332-8

2-hydroxyglutarate mediates whitening of brown adipocytes coupled to nuclear softening upon mitochondrial dysfunction

In the format provided by the authors and unedited

Supplementary figure 1

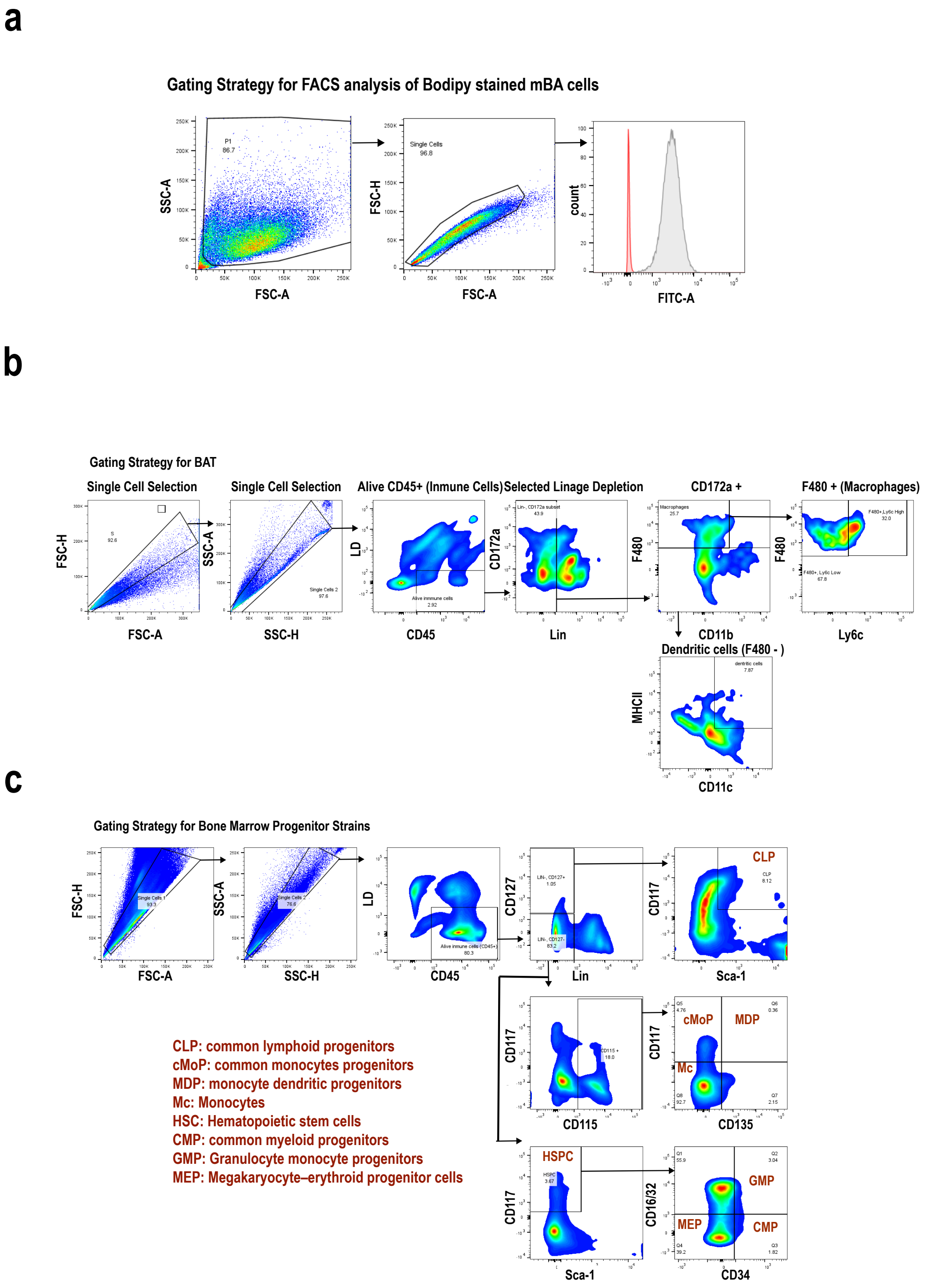

Supplementary Figure 1. Flow cytometry gating strategy for BAT and Bone Marrow progenitors

(a) Gating strategy for mBA cells, for data presented in Fig 1i, 4c, 4f, 7i and ED Fig 4f and 4i; **(b)** Gating strategy for BAT, for data presented in ED Fig 2a **(left-right)** Selection of single cells (doublet exclusion), Identification of alive immune cells using

the leukocyte marker and Fixable Viability Dye ($CD45^+$, LD^-), Selection of myeloid cells and lineage depletion of lymphoid populations ($CD172^+$, Lin^-), Subpopulation segmentation with lineage-specific markers:Macrophages ($F4/80^+$, $CD11b^+$), (E) Dendritic cells ($F4/80^-$, $MHCII^+$, $CD11c^+$), Macrophage subsets based on Ly6C surface marker expression: pro-inflammatory ($F4/80^+$, $Ly6C^{Hi}$) and anti-inflammatory ($F4/80^+$, $Ly6C^{low}$); **(c)** Gating strategy for bone marrow, for data presented in ED Fig 2b **(left-right)** Selection of single cells (doublet exclusion), Identification of alive immune cells ($CD45^+$, LD^-), Subpopulation segmentation with lineage-specific markers: Lineage depletion of mature lymphoid populations to identify progenitors that have not yet committed to a specific lineage ($CD127^+$, Lin^- for lymphoid progenitors; $CD127^-$, Lin^- for myeloid progenitors), Common lymphoid progenitors (CLP: $CD127^+$, Lin^-, CD117^int/low, Sca-1^+), Monocyte/dendritic cell progenitors (MDP: $CD127^-$, Lin^-, $CD115^+$, $CD117^+$, $CD135^+$), Common monocyte progenitors (cMoP: $CD127^-$, Lin^-, $CD115^+$, $CD117^+$, $CD135^-$) and monocytes (Mo: $CD127^-$, Lin^-, $CD115^+$, $CD117^-$, $CD135^-$), Hematopoietic stem and progenitor cells (HSPC: $CD127^-$, Lin^-, $CD117^+$, Sca-1^+), HSPC subsets: granulocyte-monocyte progenitors (GMP: $CD16/32^+$, $CD34^+$), megakaryocyte-erythroid progenitors (MEP: $CD16/32^-$, $CD34^-$), and common myeloid progenitors (CMP: $CD16/32^-$, $CD34^+$). Lineage (Lin) markers: Ter119 (erythrocytes), CD19 (B cells), CD49b (natural killer [NK] cells and some T cell subsets), NK1.1 (NK cells), CD3ε (T cells), B220 (B cells);
