# Supplementary material for: 2-hydroxyglutarate mediates whitening of brown adipocytes coupled to nuclear softening upon mitochondrial dysfunction
Source: Nat Metab. 2025 Aug 1;7(8):1593–613. doi: 10.1038/s42255-025-01332-8 (PMC12373511; doi:10.1038/s42255-025-01332-8)
Supplement: Supplementary file 2 — Reporting Summary [file 42255_2025_1332_MOESM2_ESM.pdf]

nature portfolio

Corresponding author(s): Aleksandra Trifunovic

Last updated by author(s): Mar 19, 2025

Reporting Summary

Nature Portfolio wishes to improve the reproducibility of the work that we publish. This form provides structure for consistency and transparency in reporting. For further information on Nature Portfolio policies, see our Editorial Policies and the Editorial Policy Checklist.

Statistics

For all statistical analyses, confirm that the following items are present in the figure legend, table legend, main text, or Methods section.

n/a	Confirmed	
☐	☒	The exact sample size (*n*) for each experimental group/condition, given as a discrete number and unit of measurement
☐	☒	A statement on whether measurements were taken from distinct samples or whether the same sample was measured repeatedly
☐	☒	The statistical test(s) used AND whether they are one- or two-sided<br>*Only common tests should be described solely by name; describe more complex techniques in the Methods section.*
☒	☐	A description of all covariates tested
☐	☒	A description of any assumptions or corrections, such as tests of normality and adjustment for multiple comparisons
☐	☒	A full description of the statistical parameters including central tendency (e.g. means) or other basic estimates (e.g. regression coefficient) AND variation (e.g. standard deviation) or associated estimates of uncertainty (e.g. confidence intervals)
☐	☒	For null hypothesis testing, the test statistic (e.g. *F*, *t*, *r*) with confidence intervals, effect sizes, degrees of freedom and *P* value noted<br>*Give P values as exact values whenever suitable.*
☒	☐	For Bayesian analysis, information on the choice of priors and Markov chain Monte Carlo settings
☒	☐	For hierarchical and complex designs, identification of the appropriate level for tests and full reporting of outcomes
☒	☐	Estimates of effect sizes (e.g. Cohen's *d*, Pearson's *r*), indicating how they were calculated

Our web collection on statistics for biologists contains articles on many of the points above.

Software and code

Policy information about availability of computer code

Data collection

Histological images were collected by using a slide scanner S360, Hamamatsu Slidescanner with Autoloader.
Immunofluorescence images were obtained using LSM 980 with Airyscan 2 and multiplex, Carl Zeiss Microscopy, TCS SP8, Leica Microsystems, and LSM Meta 710, Carl Zeiss Technology.
Electron microscopy images were captured by a transmission electron microscope (JOEL JEM2100 Plus) at an acceleration voltage of 80 kV, using a 4K-CCD camera, OneView (GATAN).
The rt-PCR data was collected by QuantStudio 12K Flex Software v1.6 (Applied Biosystems)
Metabolomics data was collected by Thermo Scientific Q Exactive Hybrid Quadrupole-Orbitrap Mass spectrometer (HRMS) coupled to a Dionex Ultimate 3000 UHPLC, Orbitrap Eclipse Tribrid Mass Spectrometer (Thermo Fischer Scientific).
Proteomics data was collected by liquid chromatography tandem mass spectrometry on an Orbitrap Eclipse Tribrid Mass Spectrometer (Thermo Fischer) with FAIMS Pro device.
PHGDH activity assay OD were measured in EnSpire plate reader.
For ChIP seq, libraries were prepared using KAPA Library Quantification kit (Peqlab) and the 7900HT Sequence Detection System (Applied Biosystems) The pools were sequenced on an Illumina NovaSeq6000 sequencing instrument

Data analysis

GraphPad Prism 10 for MacOS
ImageJ2 2.9.0/1.53t
Microsoft Excel for Mac 16.75.2
FlowJo Software version 10.0.8
Seahorse Wave Desktop 2.6 software
Seurat version 3.0 with R version 3.6.2

R package ComplexHeatmap version 2.12.1
GSEA windows application v4.3.2 [build 13]
UCell package version 2.0.1
Diva software (BD)
FlowJo version 10.0.8
snakePipes version 2.8.1
deeptools version 3.5.4
samtools version 1.19.2
fastqc version 0.12.1
fastp version 0.23.4
genrich version 0.6.1
R version 4.4.2
bedtools version 2.31.0

For manuscripts utilizing custom algorithms or software that are central to the research but not yet described in published literature, software must be made available to editors and reviewers. We strongly encourage code deposition in a community repository (e.g. GitHub). See the Nature Portfolio guidelines for submitting code & software for further information.

Data

Policy information about availability of data

All manuscripts must include a data availability statement. This statement should provide the following information, where applicable:

- Accession codes, unique identifiers, or web links for publicly available datasets
- A description of any restrictions on data availability
- For clinical datasets or third party data, please ensure that the statement adheres to our policy

The following reference genomes were used in the manuscript - GRCm39 mouse reference genome (https://www.ncbi.nlm.nih.gov/datasets/genome/GCF_000001635.27/) and Gencode vM25 (https://www.gencodegenes.org/mouse/release_M25.html).
Raw data for BAT tissue transcriptomics is publicly available with the accession number - GSE271207, cell transcriptomics is publicly available with the accession number - GSE271358. All raw data related to proteomics experiments on BAT tissue and mBA cells are publicly available with the accession number PXD064288. All raw data for metabolomics from BAT tissue and cells is publicly available with the doi:10.5281/zenodo.15357452 . Raw data from ChIP Seq experiment from mBA cells is publicly available with the accession number GSE296541.

Research involving human participants, their data, or biological material

Policy information about studies with human participants or human data. See also policy information about sex, gender (identity/presentation), and sexual orientation and race, ethnicity and racism.

Reporting on sex and gender	n/a
Reporting on race, ethnicity, or other socially relevant groupings	n/a
Population characteristics	n/a
Recruitment	n/a
Ethics oversight	n/a

Note that full information on the approval of the study protocol must also be provided in the manuscript.

Field-specific reporting

Please select the one below that is the best fit for your research. If you are not sure, read the appropriate sections before making your selection.

☒ Life sciences ☐ Behavioural & social sciences ☐ Ecological, evolutionary & environmental sciences

For a reference copy of the document with all sections, see nature.com/documents/nr-reporting-summary-flat.pdf

Life sciences study design

All studies must disclose on these points even when the disclosure is negative.

Sample size	Sample size was determined empirically and was based on our previous mouse work. We aimed for a number of at least 4 animals per group to allow basic statistical analysis while using justifiable number of mutant mice. All sample sizes were annotated within the respective Figure legends. Based on previous experience from similar studies, in vitro experiments with cultured cells were performed at least 3 times (3 biological replicates) to confirm reproducibility. For all experiments, biological replicates were used and each biological replicate is defined as an independent culture of cells.

Data exclusions	No animal data were excluded from the analyses.
Replication	For in vivo studies, we analyzed at least 6 mice per genotype to ensure the reproducibility of the results. For in vitro studies, we independently replicated the experiments atleast 3 times.
Randomization	No specific method of randomization was used to select animals. We compared groups of mice with different genotypes to assess the effect of specific genetic mutations in the phenotype. Group allocation was thus determined by the genotype of the mice.
Blinding	Histological evaluation of BAT sections was performed blindly.<br>Immunofluorescence imaging on cells was performed in a blinded manner.<br>Metabolomic samples were analyzed with LC-MS in a blinded manner.<br>For all other experiments, blinding was not relevant during the group generation as the group allocation was determined by the genotype of the mice.

Reporting for specific materials, systems and methods

We require information from authors about some types of materials, experimental systems and methods used in many studies. Here, indicate whether each material, system or method listed is relevant to your study. If you are not sure if a list item applies to your research, read the appropriate section before selecting a response.

Materials & experimental systems

n/a	Involved in the study	
☐	☒	Antibodies
☐	☒	Eukaryotic cell lines
☒	☐	Palaeontology and archaeology
☐	☒	Animals and other organisms
☒	☐	Clinical data
☒	☐	Dual use research of concern
☒	☐	Plants

Methods

n/a	Involved in the study	
☐	☒	ChIP-seq
☐	☒	Flow cytometry
☒	☐	MRI-based neuroimaging

Antibodies

Antibodies used

Primary antibodies-

1) Monoclonal Anti-CLPP antibody (clone 300) produced in mouse, Cat No. WH0008192M1, Sigma Aldrich
2) HSPA8/HSC70 antibody (B-6), Cat No. sc-7298, Santa Cruz
3) NDUFA9 Monoclonal Antibody (20C11B11B11), Cat No 459100, ThermoFischer Scientific
4) SDHA Monoclonal Antibody (2E3GC12FB2AE2), Cat No 459200, ThermoFischer Scientific
5) ATP5A antibody [15H4C4] - Mitochondrial Marker
6) PHGDH Polyclonal antibody, Cat no : 14719-1-AP, Proteintech
7) Anti-Histone H3 (tri methyl K4) antibody - ChIP Grade, Cat no: ab8580
8) Anti-Histone H3 (tri methyl K36) antibody - ChIP Grade, Cat no: ab9050
9) Anti-Histone H3 (tri methyl K9) antibody - ChIP Grade, Cat no: ab8898
10) Tri-Methyl-Histone H3 (Lys27) (C36B11) Rabbit mAb, Cat no: 9733
11) Histone H3 trimethyl Lys4 antibody (pAb) - ChIP Grade, Cat no: 39159, Active Motif,
12) CD45 antibody (clone 30-F11), Cat No. 103107, BioLegend
13) B220 antibody (clone RA3-6B2), Cat No. 103223, BioLegend
14) CD115 antibody (clone AFS98), Cat No. 135517, BioLegend
15) CD117 antibody (clone ack2), Cat No. 135119, BioLegend
16) CD11b antibody (clone M1/70), Cat No. 612977, BD
17) CD11c antibody (clone HL3), Cat No. 564080, BD
18) CD127 (IL-7Rα) antibody (clone A7R34), Cat No. 135021, BioLegend
19) CD135 (clone A2F10) antibody, Cat No. 135305, BioLegend
20) CD16/32 (clone 93) antibody, Cat No. 101331, BioLegend
21) CD172a (SIRPα) (clone P84) antibody, Cat No. 144015, BioLegend
22) CD19 (clone 6D5) antibody, Cat No. 115529, BioLegend
23) CD34 (clone MEC14.7) antibody, Cat No. 119325, BioLegend
24) CD3E (clone 145-2C11) antibody, Cat No. 100329, BioLegend
25) CD45 (clone 30-F11) antibody, Cat No. 103127, BioLegend
26) CD49b (clone DX5) antibody, Cat No. 108919, BioLegend
27) F4/80 (clone T45-2342) antibody, Cat No. 749284, BD
28) Ly-6A/E (Sca-1) (clone D7) antibody, Cat No. 108137, BioLegend
29) MHCII (clone M5-114.15.2) antibody, Cat No. 107625, BioLegend
30) NK1.1 (clone PK136) antibody, Cat No. 108723, BioLegend
31) Ter119 (clone TER119) antibody, Cat No. 116223, BioLegend
32) TruStain FcX™ (anti-mouse CD16/32) (clone 93) antibody, Cat No. 101319, BioLegend

Secondary antibodies-

1) anti-mouse IgG peroxidase, Sigma Aldrich, order number: A4416
2) anti-rabbit IgG peroxidase, Sigma Aldrich, order number: A6154

Validation

The primary antibodies used in this study were tested by the manufacturer
1) anti-CLPP antibody can be found in 7 citations. The manufacturer also provides antibody testing data: https://www.sigmaaldrich.com/DE/de/product/sigma/wh0008192m1
2) anti-HSPA8/HSC70 antibody (B-6) antibody can be found in 7 citations. The manufacturer also provides antibody testing data: https://www.scbt.com/p/hsc-70-antibody-b-6
3) anti-NDUFA9 antibody can be found in 67 citations. The manufacturer also provides antibody testing data: https://www.thermofisher.com/antibody/product/NDUFA9-Antibody-clone-20C11B11B11-Monoclonal/459100
4) anti-SDHA antibody can be found in 94 citations. The manufacturer also provides antibody testing data:https://www.thermofisher.com/antibody/product/SDHA-Antibody-clone-2E3GC12FB2AE2-Monoclonal/459200
5) anti-ATP5A can be found in 376 citations. The manufacturer also provides antibody testing data: https://www.abcam.com/en-de/products/primary-antibodies/atp5a-antibody-15h4c4-mitochondrial-marker-ab14748
6) anti-PHGDH antibody can be found in 49 citations. The manufacturer also provides antibody testing data:https://www.ptglab.com/products/PHGDH-Antibody-14719-1-AP.htm
7) Anti-H3K4me3 antibody can be found in 2132 citations. The manufacturer also provides antibody testing data: https://www.abcam.com/en-us/products/primary-antibodies/histone-h3-tri-methyl-k4-antibody-chip-grade-ab8580?srsltid=AfmBOopn5dDbKHXVF_ncgG3Evsq0Je46EGo-kr1CmVgoy6-hB4DrdOGO
8) Anti-H3K36me3 antibody can be found in 11 citations, The manufacturer also provides antibody testing data: https://www.abcam.com/en-us/products/primary-antibodies/histone-h3-tri-methyl-k36-antibody-epr23525-232-chip-grade-ab282572?srsltid=AfmBOooiPi416tajU24h15yOcsVVVdIIKu01_W5KwHnkIPj_B6hhntE0
9) Anti H3K9me3 antibody can be found in 1735 citations, The manufacturer also provides antibody testing data- https://www.abcam.com/en-us/products/primary-antibodies/histone-h3-tri-methyl-k9-antibody-chip-grade-ab8898?srsltid=AfmBOor0sp0zz7BiTk65d2LHNAZ0dG2Y3sbtx4xGBZfaBoOQzBikvt_v
10) Anti H3K27me3 antibody can be found in 1545 citations, The manufacturer also provides antibody testing data- https://www.cellsignal.com/products/primary-antibodies/tri-methyl-histone-h3-lys27-c36b11-rabbit-mab/9733?srsltid=AfmBOooAUjv3GmXTBgeT_vSr-DlSH0Hxle5RRhEUEPdwgwgNdhQ5ok7z
11) Anti H3K4me3 antibody used for CHIP can be found in 112 citations, The manufacturer also provides antibody testing data- https://www.activemotif.com/catalog/details/39159/histone-h3-trimethyl-lys4-antibody-pab
12) Anti CD45 antibody used in FACS can be found in 290 citations, The manufacturer provides antibody testing data- https://www.biolegend.com/en-gb/products/fitc-anti-mouse-cd45-antibody-99?GroupID=BLG1932
13) Anti B220 antibody can be found in 129 citations, The manufacturer provides antibody testing data- https://www.biolegend.com/fr-lu/products/apc-cyanine7-anti-mouse-human-cd45r-b220-antibody-1938?GroupID=GROUP658
14) Anti CD115 antibody can be found in 19 citations, The manufacturer provides antibody testing data- https://www.biolegend.com/fr-ch/products/brilliant-violet-605-anti-mouse-cd115-csf-1r-antibody-9013
15) Anti CD117 antibody can be found in 3 citations, The manufacturer provides antibody testing data- https://www.biolegend.com/fr-fr/products/brilliant-violet-510-anti-mouse-cd117-c-kit-antibody-8482?GroupID=BLG8754
16) Anti CD11b antibody can be found in 14 citations, The manufacturer provides antibody testing data- https://www.bdbiosciences.com/en-de/products/reagents/flow-cytometry-reagents/research-reagents/single-color-antibodies-ruo/buv661-rat-anti-cd11b.612977?tab=product_details
17) Anti CD11c antibody can be found in 18 citations, The manufacturer provides antibody testing data- https://www.bdbiosciences.com/en-de/products/reagents/flow-cytometry-reagents/research-reagents/single-color-antibodies-ruo/buv395-hamster-anti-mouse-cd11c.564080?tab=product_details
18) Anti CD127 antibody can be found in 16 citations, The manufacturer provides antibody testing data- https://www.biolegend.com/de-at/products/percp-cyanine5-5-anti-mouse-cd127-il-7ralpha-antibody-6196
19) Anti CD135 antibody can be found in 47 citations, The manufacturer provides antibody testing data- https://www.biolegend.com/de-de/products/pe-anti-mouse-cd135-antibody-6173?GroupID=BLG7934
20) Anti CD16/32 antibody can be found in 11 citations, The manufacturer provides antibody testing data- https://www.biolegend.com/nl-be/products/brilliant-violet-421-anti-mouse-cd16-32-antibody-8598
21) Anti CD172a antibody can be found in 4 citations, The manufacturer provides antibody testing data- https://www.biolegend.com/de-de/products/pe-dazzle-594-anti-mouse-cd172a-sirpalpha-antibody-10804
22) Anti CD19 antibody can be found in 110 citations, The manufacturer provides antibody testing data- https://www.biolegend.com/en-ie/products/apc-cyanine7-anti-mouse-cd19-antibody-3903?GroupID=BLG2221
23) Anti CD34 antibody can be found in 4 citations, The manufacturer provides antibody testing data- https://www.biolegend.com/en-gb/products/pe-cyanine7-anti-mouse-cd34-antibody-14817
24) Anti CD3E antibody can be found in 73 citations, The manufacturer provides antibody testing data- https://www.biolegend.com/en-ie/products/apc-cyanine7-anti-mouse-cd3epsilon-antibody-6070?GroupID=BLG6746
25) Anti CD45 antibody can be found in 214 citations, The manufacturer provides antibody testing data- https://www.biolegend.com/de-de/products/alexa-fluor-700-anti-mouse-cd45-antibody-3407
26) Anti CD49b antibody can be found in 12 citations, The manufacturer provides antibody testing data- https://www.biolegend.com/en-gb/products/apc-cyanine7-anti-mouse-cd49b-pan-nk-cells-antibody-8057?GroupID=BLG4768
27) Anti F4/80 antibody can be found in 3 citations, The manufacturer provides antibody testing data- https://www.bdbiosciences.com/en-de/products/reagents/flow-cytometry-reagents/research-reagents/single-color-antibodies-ruo/buv563-rat-anti-mouse-f4-80.749284?tab=product_details
28) Anti Ly-6A/E (Sca-1) antibody can be found in 1 citation, The manufacturer provides antibody testing data- https://www.biolegend.com/de-at/products/pe-dazzle-594-anti-mouse-ly-6a-e-sca-1-antibody-10190
29) Anti MHCII antibody can be found in 76 citations, The manufacturer provides antibody testing data- https://www.biolegend.com/en-ie/products/percp-cyanine5-5-anti-mouse-i-a-i-e-antibody-4282
30) Anti NK1.1 antibody can be found in 55 citations, The manufacturer provides antibody testing data- https://www.biolegend.com/en-ie/products/apc-cyanine7-anti-mouse-nk-1-1-antibody-4002?GroupID=GROUP20
31) Anti Ter119 antibody can be found in 67 citations, The manufacturer provides antibody testing data- https://www.biolegend.com/en-gb/products/apc-cyanine7-anti-mouse-ter-119-erythroid-cells-antibody-3905
32) Anti TruStain FcX™ (anti-mouse CD16/32) antibody can be found in 649 citations, The manufacturer provides antibody testing

data- https://www.biolegend.com/de-de/products/trustain-fcx-anti-mouse-cd16-32-antibody-5683?GroupID=BLG9237

Eukaryotic cell lines

Policy information about cell lines and Sex and Gender in Research

Cell line source(s)	mBA cell ine was dervied in house from control and knockout Mus musculus, from two animals pooled from each gender. HEK 293FT cells were procured commercially from Thermo Fischer Scientific, Cat No. R70007 (https://www.thermofisher.com/order/catalog/product/de/en/R70007).
Authentication	The cell lines were authenticated for their genotypes using western blotting. For 293FT cells, authentication data can be obtained from the manufacturer at the link - https://www.thermofisher.com/order/catalog/product/de/en/R70007
Mycoplasma contamination	The cell lines were routinely tested and confirmed negative for mycoplasma contamination
Commonly misidentified lines (See ICLAC register)	No ICLAC cell lines were used in the study

Animals and other research organisms

Policy information about studies involving animals; ARRIVE guidelines recommended for reporting animal research, and Sex and Gender in Research

Laboratory animals	The age of the mice used in this study ranged from 4 weeks to 22 weeks old and is specified in the legend.
Wild animals	The study did not involve wild animals
Reporting on sex	The tissue transcriptomics and proteomics findings apply only to male mice. All other in vivo experimental findings were performed on both genders and the data has been presented in a sex segregated manner, as indicated in figure legends. Where there is no mention, the sexes were selected in a randomized manner.
Field-collected samples	The study did not involve filed collected animals
Ethics oversight	All animal procedures included in the study were conducted in accordance with European National and institutional guidelines and protocols were approved by local government authorities (Landemsamt für Natur, Umwelt and Verbraucherschutz Nordrhein-Westfalen, Germany).

Note that full information on the approval of the study protocol must also be provided in the manuscript.

Plants

Seed stocks	n/a
Novel plant genotypes	n/a
Authentication	n/a

ChIP-seq

Data deposition

☒ Confirm that both raw and final processed data have been deposited in a public database such as GEO.

☒ Confirm that you have deposited or provided access to graph files (e.g. BED files) for the called peaks.

Data access links<br>*May remain private before publication.*	https://www.ncbi.nlm.nih.gov/geo/query/acc.cgi?acc=GSE296541
Files in database submission	A006200376_218434_S7_L001_R1_001.fastq.gz A006200376_218434_S7_L001_R2_001.fastq.gz<br>A006200376_218437_S8_L001_R1_001.fastq.gz A006200376_218437_S8_L001_R2_001.fastq.gz<br>A006200376_218439_S9_L001_R1_001.fastq.gz A006200376_218439_S9_L001_R2_001.fastq.gz<br>A006200376_218442_S10_L001_R1_001.fastq.gz A006200376_218442_S10_L001_R2_001.fastq.gz<br>A006200376_218444_S11_L001_R1_001.fastq.gz A006200376_218444_S11_L001_R2_001.fastq.gz<br>A006200376_218446_S12_L001_R1_001.fastq.gz A006200376_218446_S12_L001_R2_001.fastq.gz

A006200376_218448_S13_L001_R1_001.fastq.gz A006200376_218448_S13_L001_R2_001.fastq.gz
A006200376_218450_S14_L001_R1_001.fastq.gz A006200376_218450_S14_L001_R2_001.fastq.gz
A006200376_218452_S15_L001_R1_001.fastq.gz A006200376_218452_S15_L001_R2_001.fastq.gz
A006200376_218454_S16_L001_R1_001.fastq.gz A006200376_218454_S16_L001_R2_001.fastq.gz
A006200376_218456_S17_L001_R1_001.fastq.gz A006200376_218456_S17_L001_R2_001.fastq.gz
A006200376_218458_S18_L001_R1_001.fastq.gz A006200376_218458_S18_L001_R2_001.fastq.gz
A006200376_218468_S23_L001_R1_001.fastq.gz A006200376_218468_S23_L001_R2_001.fastq.gz
A006200376_218470_S24_L001_R1_001.fastq.gz A006200376_218470_S24_L001_R2_001.fastq.gz
A006200376_218472_S25_L001_R1_001.fastq.gz A006200376_218472_S25_L001_R2_001.fastq.gz
WT-UT.narrowPeak
WT-HG.narrowPeak
KO-UT.narrowPeak

Genome browser session (e.g. UCSC)

no longer applicable

Methodology

Replicates

4 replicates, per condition WT-UT, WT-HG, KO-UT

Sequencing depth

25 million reads

Antibodies

H3K4me3 (Active Motif, cat no:39159)

Peak calling parameters

Genrich -t A006200376_218434_S7_L001.sorted.bam,A006200376_218439_S9_L001.sorted.bam,A006200376_218437_S8_L001.sorted.bam,A006200376_218442_S10_L001.sorted.bam -c A006200376_218468_S23_L001.sorted.bam,A006200376_218468_S23_L001.sorted.bam,A006200376_218468_S23_L001.sorted.bam,A006200376_218468_S23_L001.sorted.bam -o WT-UT.narrrowPeak.bed -f WT-UT.bedgraph.log -R WT-UT.duplicates -b WT-UT_reads_fragments_intervals.bed -k WT-UT_pileups.bdgr -r -x -q 0.05 -a 20.0 -e chrX,chrY,chrM,GL456210.1,GL456211.1,GL456212.1,GL456213.1,GL456216.1,GL456219.1,GL456221.1,GL456233.1,GL456239.1,GL456350.1,GL456354.1,GL456359.1,GL456360.1,GL456366.1,GL456367.1,GL456368.1,GL456370.1,GL456372.1,GL456378.1,GL456379.1,GL456381.1,GL456382.1,GL456383.1,GL456385.1,GL456387.1,GL456389.1,GL456390.1,GL456392.1,GL456393.1,GL456394.1,GL456396.1,JH584292.1,JH584293.1,JH584294.1,JH584295.1,JH584296.1,JH584297.1,JH584298.1,JH584299.1,JH584300.1,JH584301.1,JH584302.1,JH584303.1,JH584304.1,chr1_GL456210v1_random,chr1_GL456211v1_random,chr1_GL456212v1_random,chr1_GL456221v1_random,chr1_GL456239v1_random,chr1_MU069434v1_random,chr4_JH584295v1_random,chr5_GL456354v1_random,chr5_JH584296v1_random,chr5_JH584297v1_random,chr5_JH584298v1_random,chr5_JH584299v1_random,chr7_GL456219v1_random,chrM,chrUn_GL456359v1,chrUn_GL456360v1,chrUn_GL456366v1,chrUn_GL456367v1,chrUn_GL456368v1,chrUn_GL456370v1,chrUn_GL456372v1,chrUn_GL456378v1,chrUn_GL456379v1,chrUn_GL456381v1,chrUn_GL456382v1,chrUn_GL456383v1,chrUn_GL456385v1,chrUn_GL456387v1,chrUn_GL456389v1,chrUn_GL456390v1,chrUn_GL456392v1,chrUn_GL456394v1,chrUn_GL456396v1,chrUn_JH584304v1,chrUn_MU069435v1,chrX_GL456233v2_random,chrY_JH584300v1_random,chrY_JH584301v1_random,chrY_JH584302v1_random,chrY_JH584303v1_random -E /mnt/c/Users/trotos/Desktop/bwa/mm39.excluderanges.bed -v -z

Genrich -t A006200376_218444_S11_L001.sorted.bam,A006200376_218446_S12_L001.sorted.bam,A006200376_218448_S13_L001.sorted.bam,A006200376_218450_S14_L001.sorted.bam -c A006200376_218470_S24_L001.sorted.bam,A006200376_218470_S24_L001.sorted.bam,A006200376_218470_S24_L001.sorted.bam,A006200376_218470_S24_L001.sorted.bam -o WT-HG.narrrowPeak.bed -f WT-HG.bedgraph.log -R WT-HG.duplicates -b WT-HG_reads_fragments_intervals.bed -k WT-HG_pileups.bdgr -r -x -q 0.05 -a 20.0 -e chrX,chrY,chrM,GL456210.1,GL456211.1,GL456212.1,GL456213.1,GL456216.1,GL456219.1,GL456221.1,GL456233.1,GL456239.1,GL456350.1,GL456354.1,GL456359.1,GL456360.1,GL456366.1,GL456367.1,GL456368.1,GL456370.1,GL456372.1,GL456378.1,GL456379.1,GL456381.1,GL456382.1,GL456383.1,GL456385.1,GL456387.1,GL456389.1,GL456390.1,GL456392.1,GL456393.1,GL456394.1,GL456396.1,JH584292.1,JH584293.1,JH584294.1,JH584295.1,JH584296.1,JH584297.1,JH584298.1,JH584299.1,JH584300.1,JH584301.1,JH584302.1,JH584303.1,JH584304.1,chr1_GL456210v1_random,chr1_GL456211v1_random,chr1_GL456212v1_random,chr1_GL456221v1_random,chr1_GL456239v1_random,chr1_MU069434v1_random,chr4_JH584295v1_random,chr5_GL456354v1_random,chr5_JH584296v1_random,chr5_JH584297v1_random,chr5_JH584298v1_random,chr5_JH584299v1_random,chr7_GL456219v1_random,chrM,chrUn_GL456359v1,chrUn_GL456360v1,chrUn_GL456366v1,chrUn_GL456367v1,chrUn_GL456368v1,chrUn_GL456370v1,chrUn_GL456372v1,chrUn_GL456378v1,chrUn_GL456379v1,chrUn_GL456381v1,chrUn_GL456382v1,chrUn_GL456383v1,chrUn_GL456385v1,chrUn_GL456387v1,chrUn_GL456389v1,chrUn_GL456390v1,chrUn_GL456392v1,chrUn_GL456394v1,chrUn_GL456396v1,chrUn_JH584304v1,chrUn_MU069435v1,chrX_GL456233v2_random,chrY_JH584300v1_random,chrY_JH584301v1_random,chrY_JH584302v1_random,chrY_JH584303v1_random -E /mnt/c/Users/trotos/Desktop/bwa/mm39.excluderanges.bed -v -z

Genrich -t A006200376_218452_S15_L001.sorted.bam,A006200376_218454_S16_L001.sorted.bam,A006200376_218456_S17_L001.sorted.bam,A006200376_218458_S18_L001.sorted.bam -c A006200376_218472_S25_L001.sorted.bam,A006200376_218472_S25_L001.sorted.bam,A006200376_218472_S25_L001.sorted.bam,A006200376_218472_S25_L001.sorted.bam -o KO-UT.narrrowPeak.bed -f KO-UT.bedgraph.log -R KO-UT.duplicates -b KO-UT_reads_fragments_intervals.bed -k KO-UT_pileups.bdgr -r -x -q 0.05 -a 20.0 -e chrX,chrY,chrM,GL456210.1,GL456211.1,GL456212.1,GL456213.1,GL456216.1,GL456219.1,GL456221.1,GL456233.1,GL456239.1,GL456350.1,GL456354.1,GL456359.1,GL456360.1,GL456366.1,GL456367.1,GL456368.1,GL456370.1,GL456372.1,GL456378.1,GL456379.1,GL456381.1,GL456382.1,GL456383.1,GL456385.1,GL456387.1,GL456389.1,GL456390.1,GL456392.1,GL456393.1,GL456394.1,GL456396.1,JH584292.1,JH584293.1,JH584294.1,JH584295.1,JH584296.1,JH584297.1,JH584298.1,JH584299.1,JH584300.1,JH584301.1,JH584302.1,JH584303.1,JH584304.1,chr1_GL456210v1_random,chr1_GL456211v1_random,chr1_GL456212v1_random,chr1_GL456221v1_random,chr1_GL456239v1_random,chr1_MU069434v1_random,chr4_JH584295v1_random,chr5_GL456354v1_random,chr

5_JH584296v1_random,chr5_JH584297v1_random,chr5_JH584298v1_random,chr5_JH584299v1_random,chr7_GL456219v1_random,chrM,chrUn_GL456359v1,chrUn_GL456360v1,chrUn_GL456366v1,chrUn_GL456367v1,chrUn_GL456368v1,chrUn_GL456370v1,chrUn_GL456372v1,chrUn_GL456378v1,chrUn_GL456379v1,chrUn_GL456381v1,chrUn_GL456382v1,chrUn_GL456383v1,chrUn_GL456385v1,chrUn_GL456387v1,chrUn_GL456389v1,chrUn_GL456390v1,chrUn_GL456392v1,chrUn_GL456394v1,chrUn_GL456396v1,chrUn_JH584304v1,chrUn_MU069435v1,chrX_GL456233v2_random,chrY_JH584300v1_random,chrY_JH584301v1_random,chrY_JH584302v1_random,chrY_JH584303v1_random -E /mnt/c/Users/trotos/Desktop/bwa/mm39.excluderanges.bed -v -z

Data quality

Genrich called peaks for multiple replicates collectively. First, it analyzed the replicates separately, with p-values calculated for each. At each genomic position, the multiple replicates' p-values were then combined by Fisher's method. The combined p-values were converted to q-values, and peaks were called.
KO-UT peaks at FDR 0.05 15118 AUC 20
WT-HG peaks at FDR 0.05 14060 AUC 20
WT-UT peaks at FDR 0.05 12342 AUC 20

Software

We used the snakePipes pipeline the module for ChIP-seq. https://github.com/maxplanck-ie/snakepipes. Other softwares used during analysis were -
snakePipes version 2.8.1
deeptools version 3.5.4
samtools version 1.19.2
fastqc version 0.12.1
fastp version 0.23.4
genrich version 0.6.1
R version 4.4.2
bedtools version 2.31.0

Flow Cytometry

Plots

Confirm that:

☒ The axis labels state the marker and fluorochrome used (e.g. CD4-FITC).

☒ The axis scales are clearly visible. Include numbers along axes only for bottom left plot of group (a 'group' is an analysis of identical markers).

☐ All plots are contour plots with outliers or pseudocolor plots.

☒ A numerical value for number of cells or percentage (with statistics) is provided.

Methodology

Sample preparation

For single cell suspension, bone marrow was flashed with 10 ml of FACS buffer from the femur of adult mice using a 27 G needle and syringe tough a 40 um strainer. Brow adipose tissue was dissected and immediately transferred in ice-cold digesting solution. Tissues were cut in little pieces with scissors and incubated in digesting solution containing Collagenase IV (1 mg/ml), and DNase I (0.01 mg/ml) in RPMI for 40 min at 37°C in the shaker. Digestion was stopped by adding 10% FBS on ice. Tissue homogenates where smashed and washed with FACS buffer against a 70 um strainer using the back of a syringe. Pellets were resuspended in 1 ml red blood cell lysis buffer (Roche) and incubated for 10 min at RT for lysis of erythrocytes. Subsequently, 10 ml FACS buffer (5% FCS in PBS) was added and cells were centrifuged at 300 g for 5 min at 4C. The cells were pre-incubated with fix viability dye in PBS (1:1000) for 10 min at RT, for BAT a mix of anti-mouse FcγIII/II receptor (CD16/CD32)-blocking antibodies (1:500) was added. After wash with FACS buffer, cells were stained with the fluorochrome-conjugated antibodies (1:100 0.25– 1 μg; listed below). Only BAT cells were fixed and permeabilized with FoxP3 kit according to manufacturer instructions, and then incubated with iNOS and Arg-1 antibodies 20 min at RT for intracellular staining

Instrument

FACS Symphony ™ A3 flow cytometer (BD)

Software

Acquisition software: Diva software (BD) . Analysis software: FlowJo version 10.0.8, LLC.

Cell population abundance

For absolute quantification, 25μL of counting beads (at a concentration of 1,000 beads/μL) were added to 250μL of single-cell suspension. After gating, the absolute number of cells (cells/μL) was determined using the following equation:
Absolute count (cells/μL) = (Cell count × Counting beads volume) / (Counting bead count × Sample volume) × Counting bead concentration.
Each population was expressed in absolute numbers (cells/μL), and results were further normalized to the initial tissue weight (grams of BAT) for comparison across samples.

Gating strategy

For both brown adipose tissue (BAT) and bone marrow, the gating strategy begins with the selection of single cells by excluding doublets, ensuring that only individual cellular events are analyzed. Live immune cells are then identified by gating for CD45-positive cells that are negative for the viability dye, thereby excluding dead cells and non-immune populations from further analysis. To focus on progenitor populations, mature lymphoid and myeloid cells are excluded using a lineage (Lin) cocktail containing markers for erythrocytes, B cells, T cells, and NK cells. Specifically, the Lin cocktail includes Ter119 (erythrocytes), CD19 and B220 (B cells), CD49b and NK1.1 (NK cells and some T cell subsets), and CD3ε (T cells)
Subpopulation in BAT:
Myeloid cells are then selected by gating on CD172a-positive cells and Lin-negative. Subsequent gating steps use lineage-

specific markers to further resolve myeloid subpopulations: macrophages are identified as F4/80-positive, CD11b-positive cells, while dendritic cells are defined as F4/80-negative, MHCII-positive, CD11c-positive cells. Macrophage subsets are then distinguished based on Ly6C expression, with F4/80-positive, Ly6C^hi cells representing pro-inflammatory macrophages, and F4/80-positive, Ly6C^lo cells representing anti-inflammatory macrophages.
Subpopulation in Bone Marrow:

Lymphoid progenitors are identified as CD127-positive, Lin-negative cells, while myeloid progenitors are CD127-negative, Lin-negative. Within these populations, further markers are used to distinguish specific progenitor subsets:

• Common lymphoid progenitors (CLP): $CD127^+$, Lin^-, CD117^int/low, $Sca\text{-}1^+$
• Monocyte/dendritic cell progenitors (MDP): $CD127^-$, Lin^-, $CD115^+$, $CD117^+$, $CD135^+$
• Common monocyte progenitors (cMoP): $CD127^-$, Lin^-, $CD115^+$, $CD117^+$, $CD135^-$
• Monocytes: $CD127^-$, Lin^-, $CD115^+$, $CD117^-$, $CD135^-$
• Hematopoietic stem and progenitor cells (HSPC): $CD127^-$, Lin^-, $CD117^+$, $Sca\text{-}1^+$
• HSPC subsets:
  -Granulocyte-monocyte progenitors (GMP): $CD16/32^+$, $CD34^+$
  -Megakaryocyte-erythroid progenitors (MEP): $CD16/32^-$, $CD34^-$
  - Common myeloid progenitors (CMP): $CD16/32^-$, $CD34^+$

☒ Tick this box to confirm that a figure exemplifying the gating strategy is provided in the Supplementary Information.
