# Supplementary material for: 2-hydroxyglutarate mediates whitening of brown adipocytes coupled to nuclear softening upon mitochondrial dysfunction
Source: Nat Metab. 2025 Aug 1;7(8):1593–613. doi: 10.1038/s42255-025-01332-8 (PMC12373511; doi:10.1038/s42255-025-01332-8)

Raw data for Fig 1a

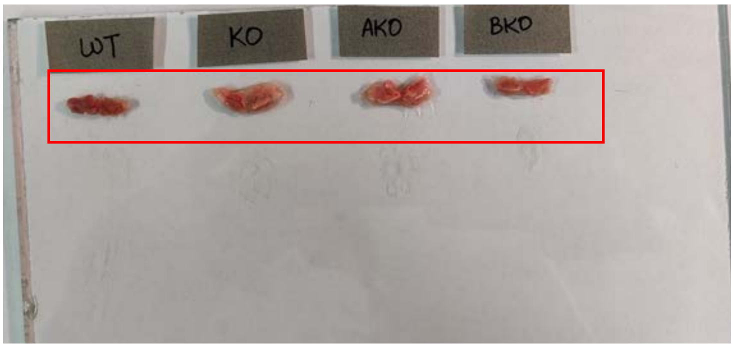

Raw data for Fig 1c

WT	KO
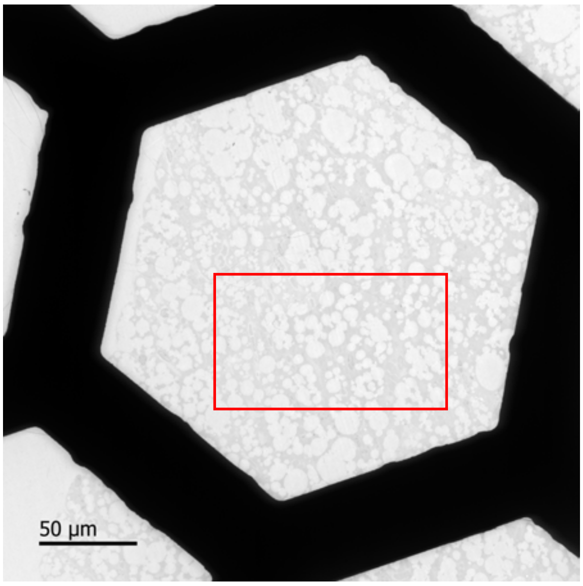	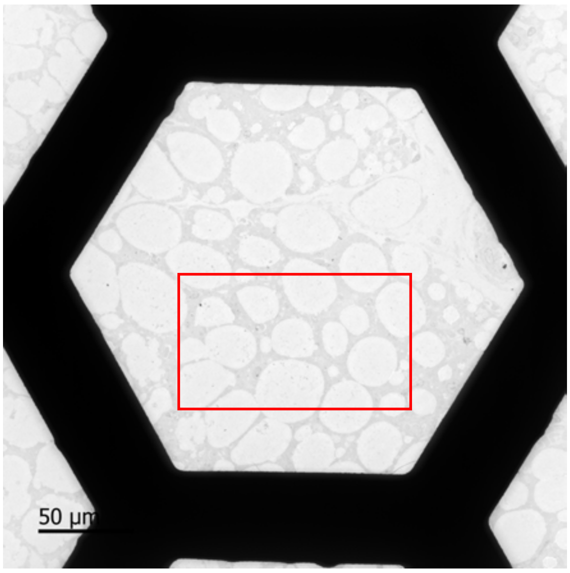

Raw data for Fig 1d

Order for loading –

1-3: WT, 4-6: KO, 7-9: AKO, 10-12: BKO

Complex I

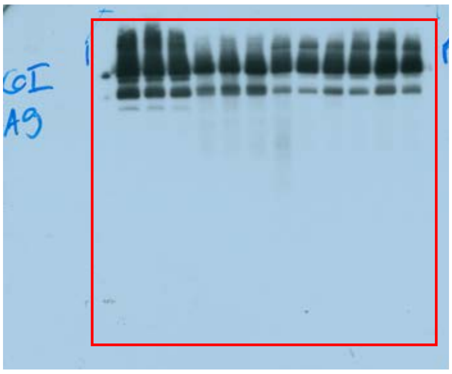

Complex V

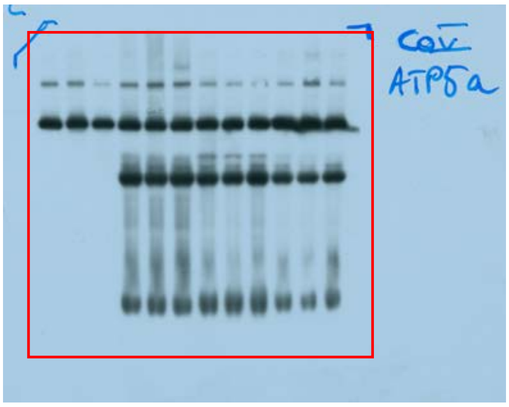

SDHA for Complex I

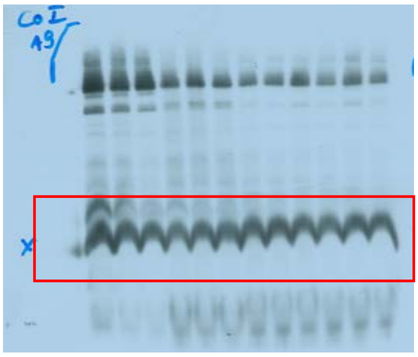

SDHA for Complex V

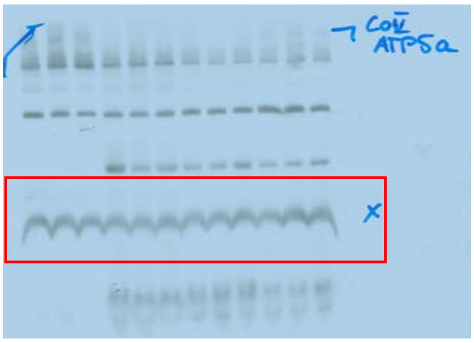

Supplement: Supplementary file 6 — Uncropped blot scans, low mag TEM image and tissue images [file 42255_2025_1332_MOESM6_ESM.pdf]
