# Supplementary figures and images for: 2-hydroxyglutarate mediates whitening of brown adipocytes coupled to nuclear softening upon mitochondrial dysfunction
Source: Nat Metab. 2025 Aug 1;7(8):1593–613. doi: 10.1038/s42255-025-01332-8 (PMC12373511; doi:10.1038/s42255-025-01332-8)

Raw data for Fig 3I

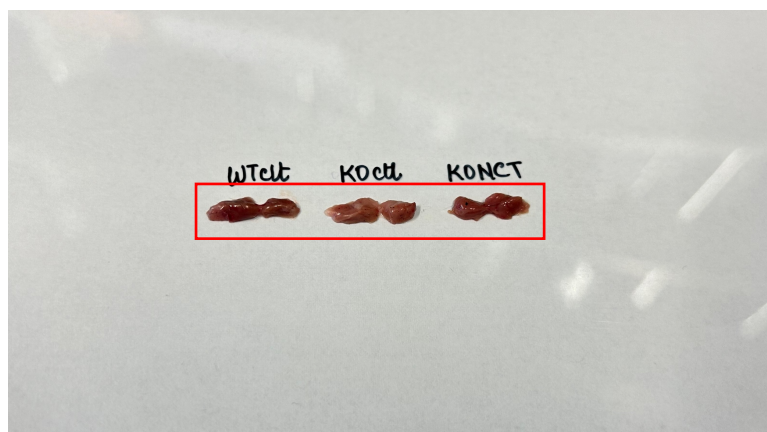

Supplement: Supplementary file 7 — Uncropped tissue images [file 42255_2025_1332_MOESM7_ESM.pdf]

Raw data for Fig 5a

WT

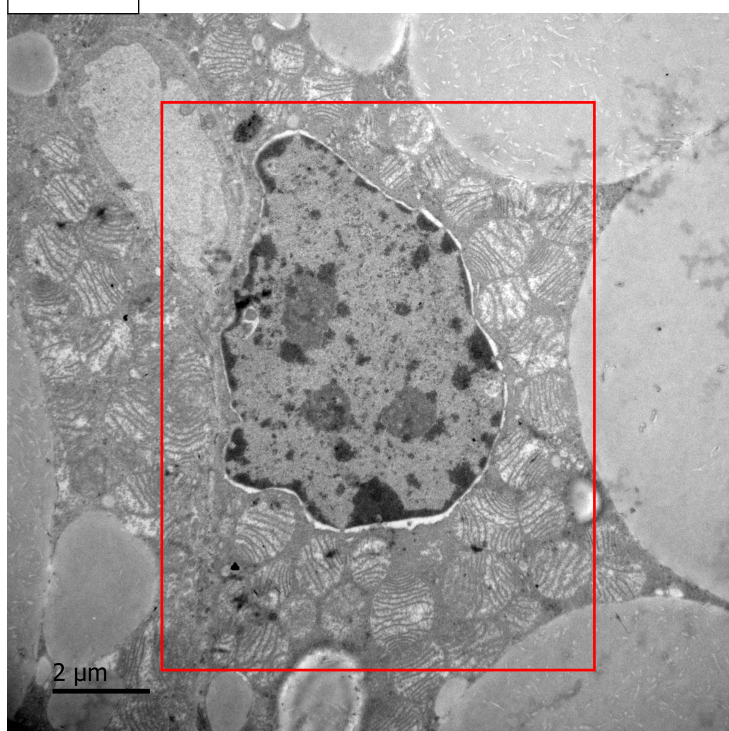

KO

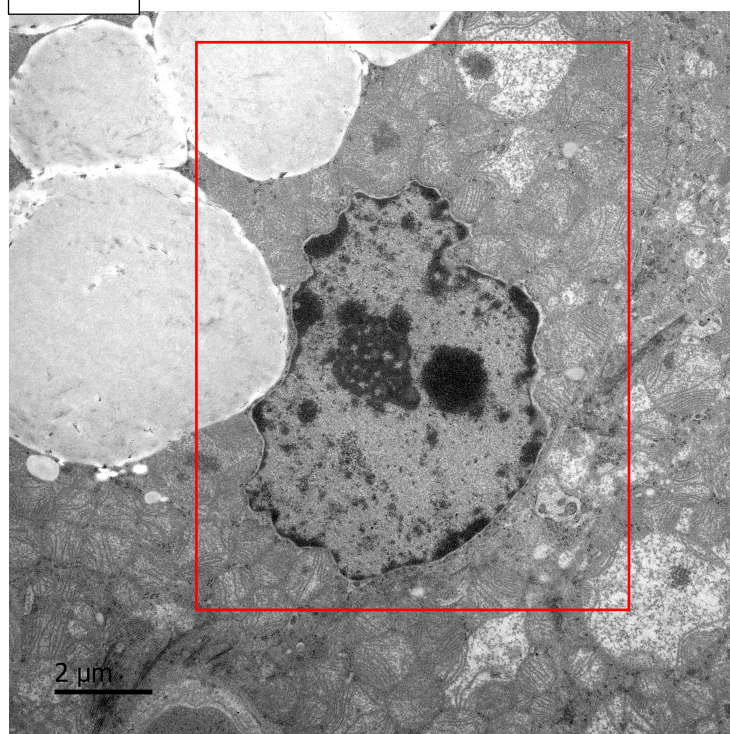

Supplement: Supplementary file 8 — Uncropped TEM image [file 42255_2025_1332_MOESM8_ESM.pdf]

### Raw data for ED Fig 3c

Order for loading –

1-3: WT-CM, 4-6: WT-PDM, 7-9: KO-CM, 10-12: KO-PDM

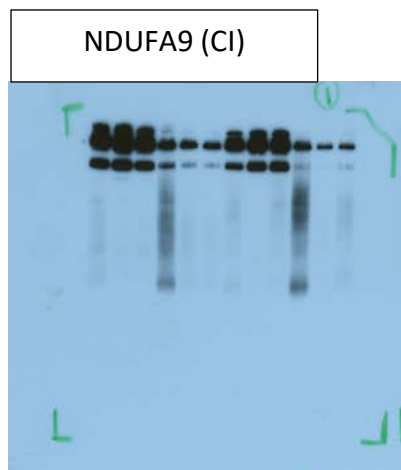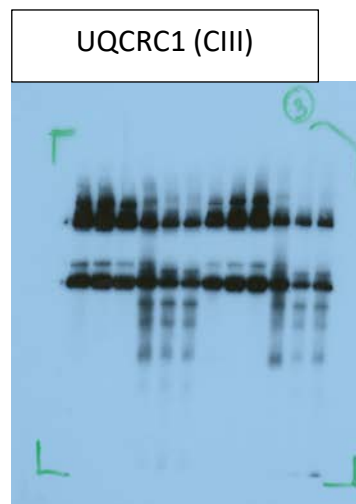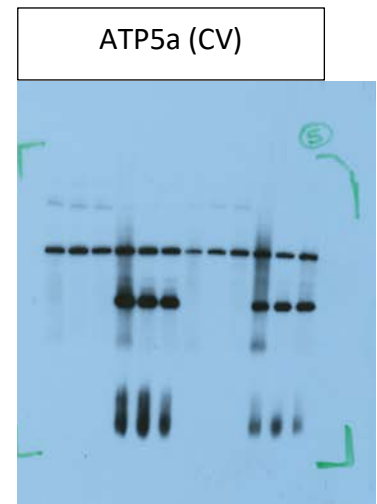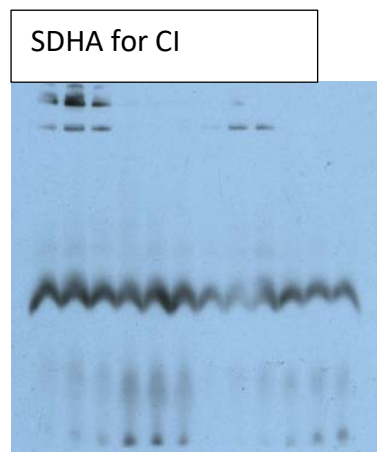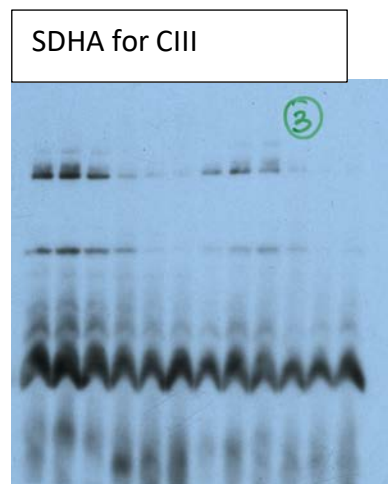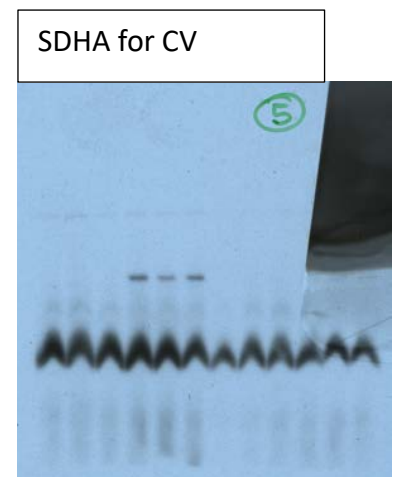

Supplement: Supplementary file 10 — Uncropped blot scans [file 42255_2025_1332_MOESM10_ESM.pdf]

Order for loading –

1-3: WT, 4-6: WT+HG, 7-9: KO, 10-12: NA

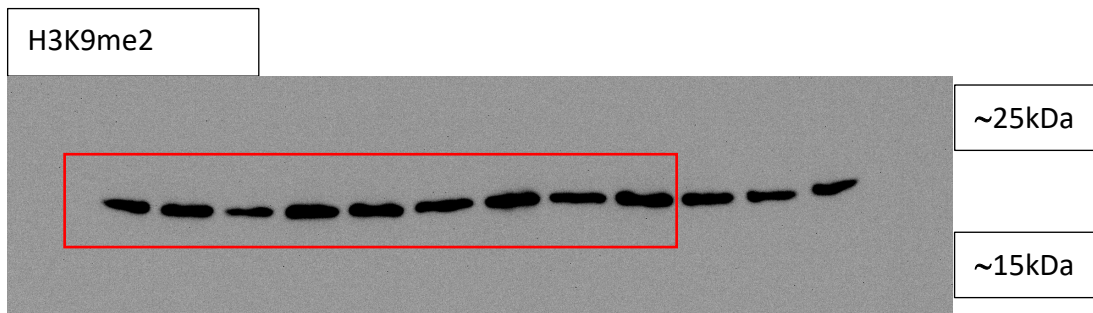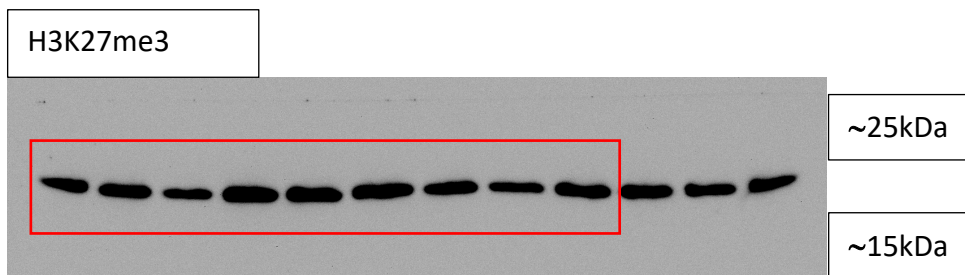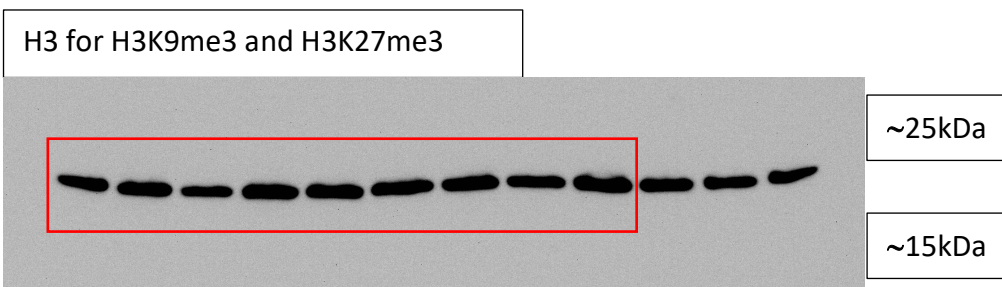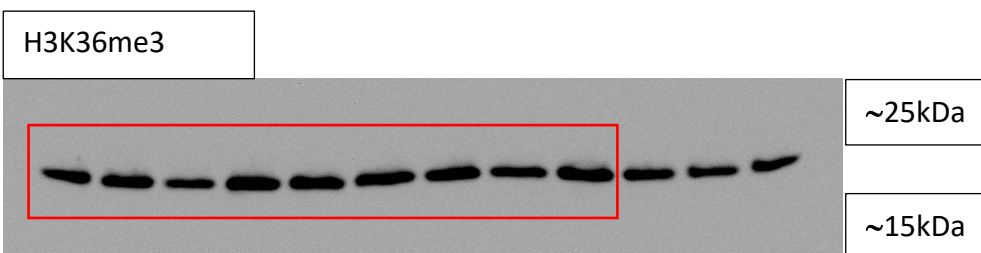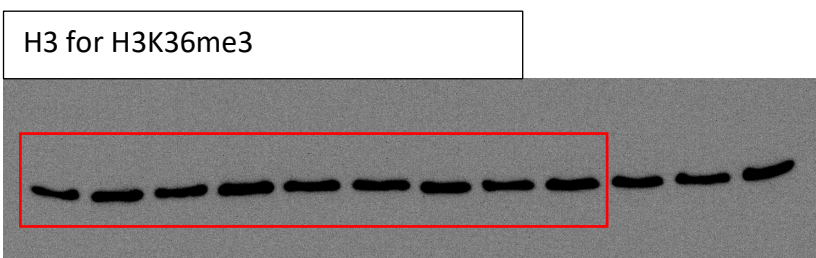

Supplement: Supplementary file 12 — Uncropped blot scans [file 42255_2025_1332_MOESM12_ESM.pdf]
