# Supplementary material for: 2-hydroxyglutarate mediates whitening of brown adipocytes coupled to nuclear softening upon mitochondrial dysfunction
Source: Nat Metab. 2025 Aug 1;7(8):1593–613. doi: 10.1038/s42255-025-01332-8 (PMC12373511; doi:10.1038/s42255-025-01332-8)

Raw data for ED Fig 1a

Order for loading –

1-3: WT, 4-6: KO, 7-9: AKO, 10-12: BKO

CLPP

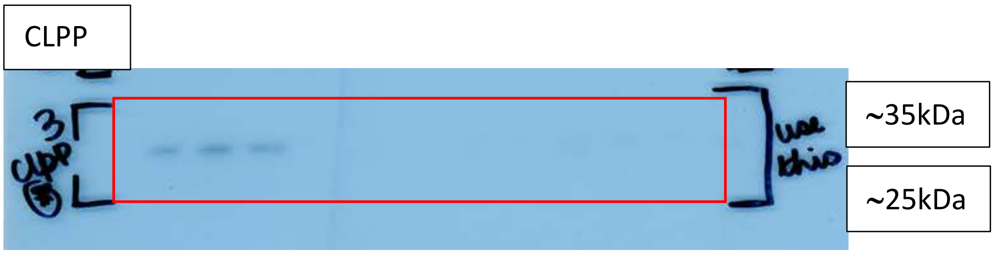

~35kDa

~25kDa

HSC70 for CLPP

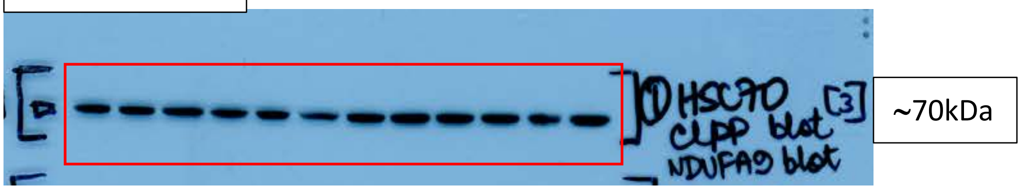

~70kDa

Raw data for ED Fig 1d

Order for loading –

1-3: WT, 4-6: KO, 7-9: AKO, 10-12: BKO

In gel activity for Complex I

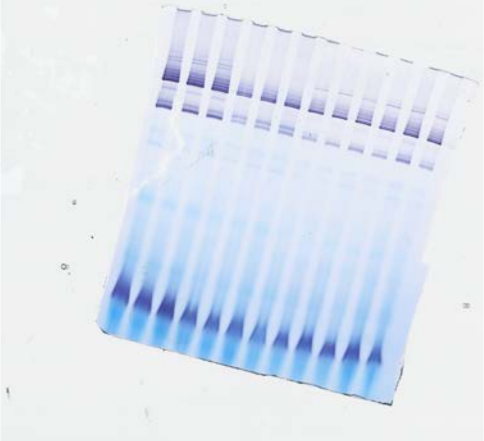

Coomassie for loading

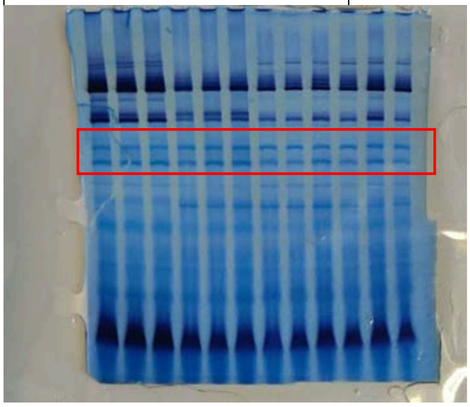

In gel activity for Complex V

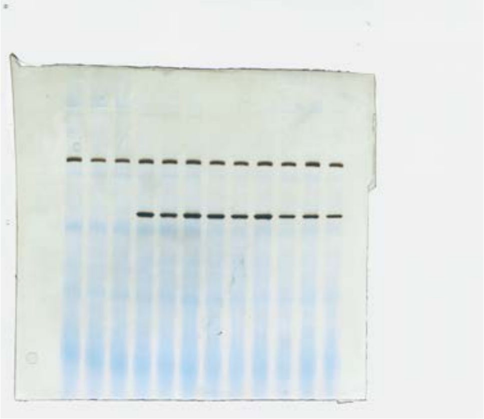

Coomassie for loading

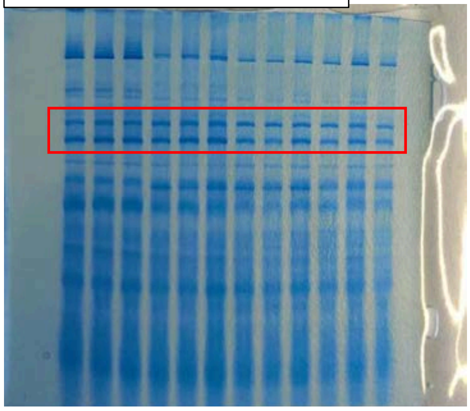

Supplement: Supplementary file 9 — Uncropped blots and gel scans [file 42255_2025_1332_MOESM9_ESM.pdf]
