# Supplementary material for: 2-hydroxyglutarate mediates whitening of brown adipocytes coupled to nuclear softening upon mitochondrial dysfunction
Source: Nat Metab. 2025 Aug 1;7(8):1593–613. doi: 10.1038/s42255-025-01332-8 (PMC12373511; doi:10.1038/s42255-025-01332-8)
Supplement: Supplementary file 11 — Uncropped blot scans [file 42255_2025_1332_MOESM11_ESM.pdf]

Raw data for ED Fig 4c

Order for loading –

1-3: NA, 4-6: WT, 7-9: KO, 10-12: NA

CLPP

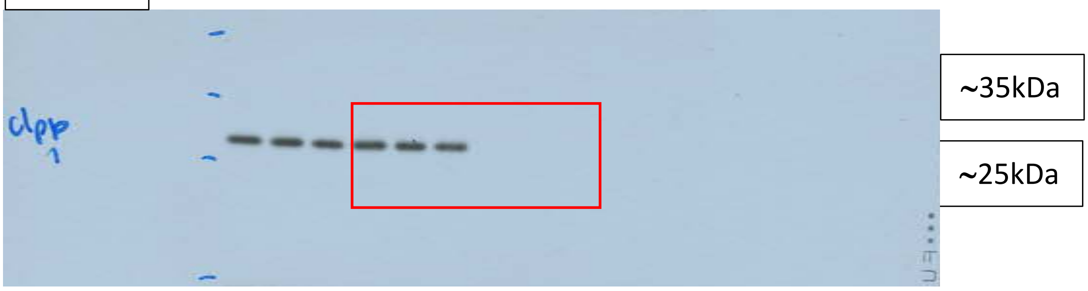

~35kDa

~25kDa

HSC70 for CLPP

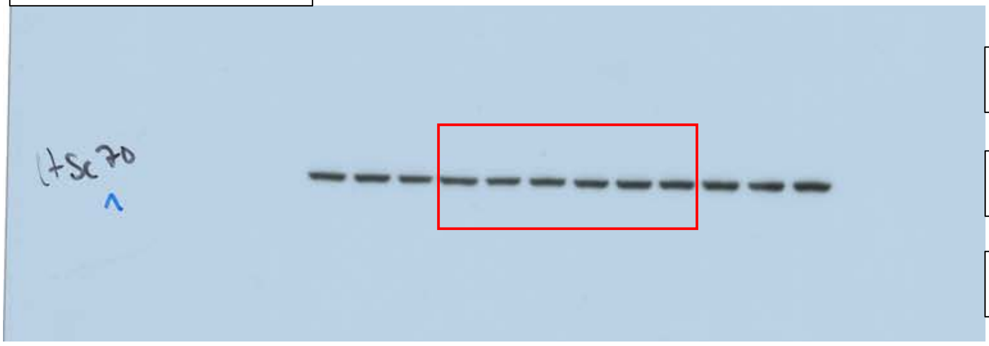

~100kDa

~70kDa

~55kDa

PHGDH

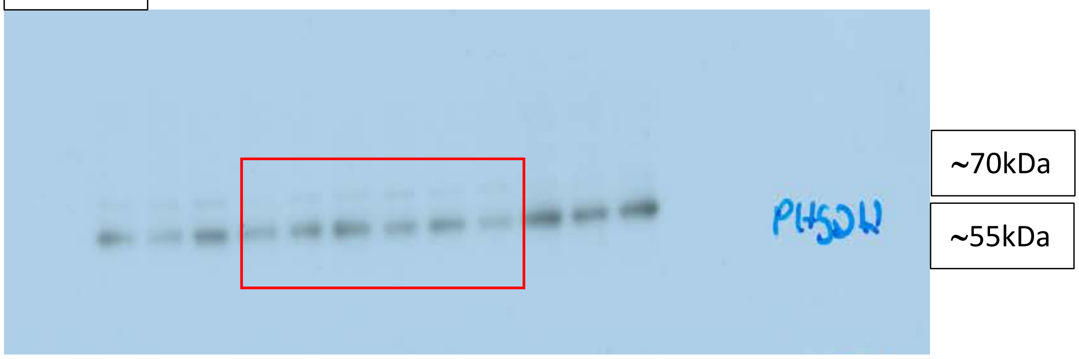

~70kDa

~55kDa

HSC70 for PHGDH

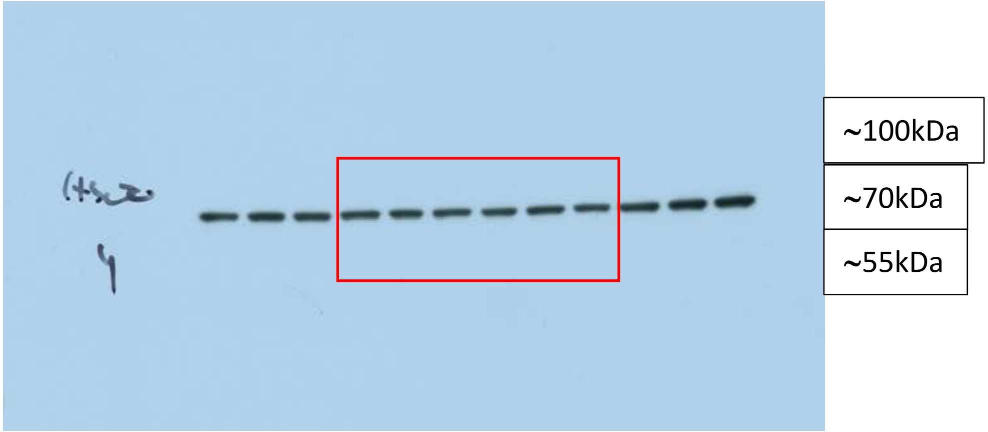

~100kDa

~70kDa

~55kDa

Raw data for ED Fig 4g

Order for loading –

1-3: WT, 4-6: KO, 7-9: WT+ISRIB, 10-12: KO+ISRIB

SHMT2 and MTHFD2

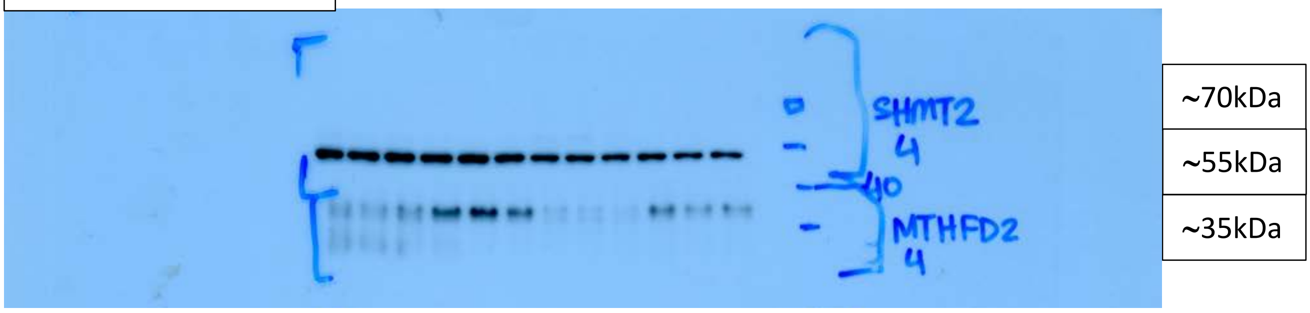

~70kDa

~55kDa

~35kDa

HSC70 for SHMT2 and MTHFD2

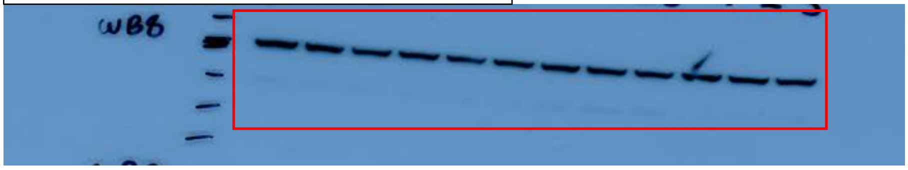

Order for loading –

1-3:KO+ISRIB, 4-6: WT+ISRIB, 7-9: KO, 10-12: WT

ALDH18A1

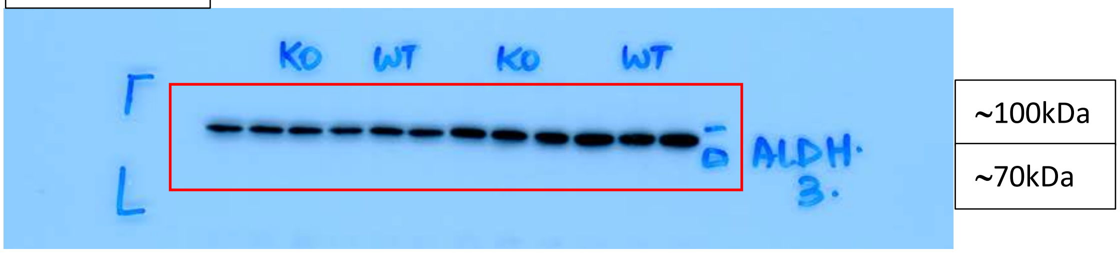

~100kDa

~70kDa

pEIF2a

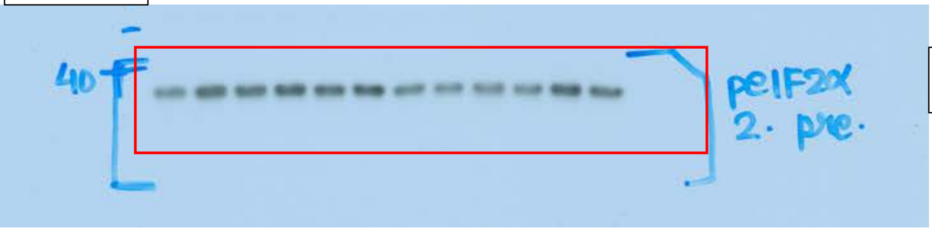

~40kDa

HSC70 for ALDH18A1 and pEIF2a

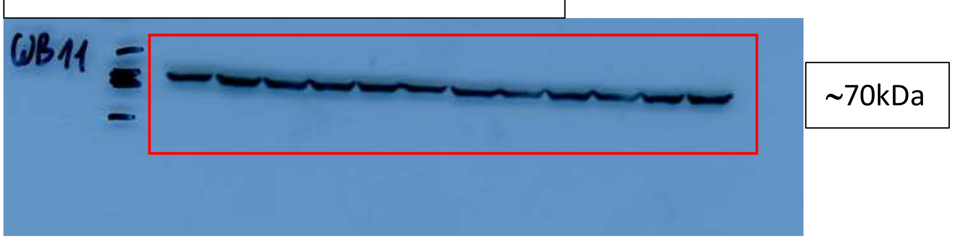

~70kDa

Order for loading –

1-3: WT, 4-6: KO, 7-9: WT+ISRIB, 10-12: KO+ISRIB

PHGDH

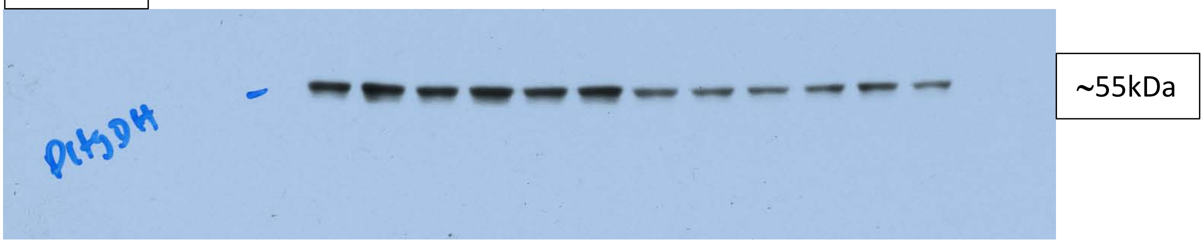

~55kDa

HSC70 for PHGDH

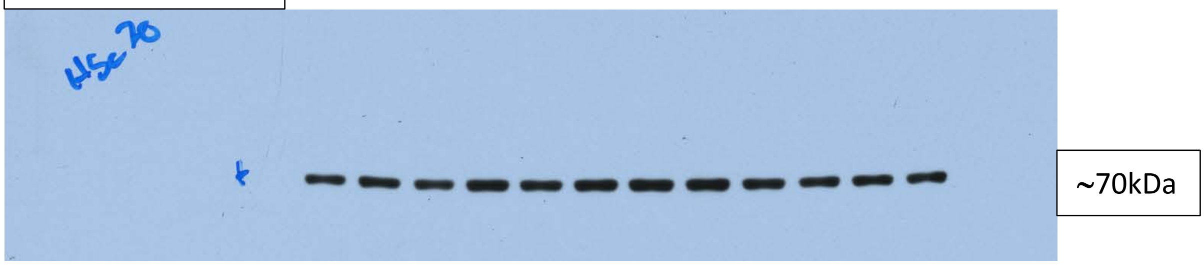

~70kDa
